# Structural Engineering and Optimization of Zwitterionic Salts for Expeditious Discovery of Thermoresponsive Materials

**DOI:** 10.3390/molecules27010257

**Published:** 2021-12-31

**Authors:** Yen-Ho Chu, Chien-Yuan Chen, Jin-Syuan Chen

**Affiliations:** Department of Chemistry and Biochemistry, National Chung Cheng University, Chiayi 62102, Taiwan, China; passercloud@gmail.com (C.-Y.C.); a0987321298@gmail.com (J.-S.C.)

**Keywords:** ionic liquid, zwitter-ionic liquids, UCST-type phase transitions, thermoresponsive material

## Abstract

This work reported the discovery of *N*-triflimide (NTf)-based zwitter-ionic liquids (ZILs) that exhibit UCST-type phase transitions in water, and their further structural optimization in fine-tuning polarity to ultimately afford newfangled thermosensitive materials carrying attractive and biocompatible *T_c_* values that clearly demonstrated the true value of the tunability of ZIL structure. This research established that with non-aromatic, acyclic ZILs as small-molecule thermoresponsive materials, their mixing and de-mixing with water triggered by temperatures are entirely reversible.

## 1. Introduction

Thermoresponsive ionic liquids (TILs) belong to the class of stimuli-responsive, smart materials [1,2] that change properties with temperatures in their environment, and can be chemically devised to respond reversibly to external temperature changes [3,4,5]. In a rigorous sense, these TILs display a miscibility gap in their temperature-composition phase diagram and, depending on whether the gap is found at high or low temperatures, an upper or lower critical solution temperature occurs (UCST or LCST), respectively.

Both UCST and LCST systems are two typical phase behaviors of thermoresponsive materials with solvents [5]. In UCST systems, the solubility of TIL in a solvent increases with increasing temperature, and a homogeneous system is formed above a certain point (critical temperature, *T_c_*), namely the components are miscible at all concentrations above the UCST. Conversely, in LCST systems, two immiscible solutions homogeneously mix upon cooling. These TIL-water systems, however, have the potential to detract their properties as a result of unwanted ion pair formation through ion exchange after being mixed with other ions. It is therefore envisioned that designing zwitter-ionic liquid (ZIL)-water systems showing thermoresponsive behavior should be highly attractive for applications involved in extraction and separation, since the ion pairs of ZILs are fixed covalently even after adding other charged components [3,4].

In this work we are reporting the synthesis of ‘choline-like’ ZILs, experimental screening of their ability to perform UCST-type phase transition in water, and further structural fine-engineering and optimization of candidate ZILs to afford bio-attractive TILs as small-molecule thermoresponsive materials for a preliminary application in biomolecular recognition study. These ZILs exhibit distinct properties from common ionic liquids (ILs) and conventional molecular solvents (e.g., prevention of undesired ion exchange and negligible vapor pressure, respectively) and should be of valuable media for extraction and separation of target biomaterials with high selectivity and efficiency.

## 2. Results and Discussion

Nockemann and coworkers were first to report that choline bis(trifluoromethylsulfonyl)imide, [choline][NTf_2_] (**IL 1**), a supercooled ionic liquid at room temperature (mp, 30 °C), exhibited UCST phase transition with water (*T_c_* = 72 °C at 1:1 mass ratio) [6]. Crystal analysis of **IL 1** revealed that the hydrogen bonding between the hydroxyl group on choline cation and one sulfonyl oxygen on NTf_2_ anion was responsible for this temperature-dependent UCST behavior; that is, **IL 1** is immiscible with water at room temperature, but forms homogeneous solution at temperatures above 72 °C [6].

**Figure 1:**
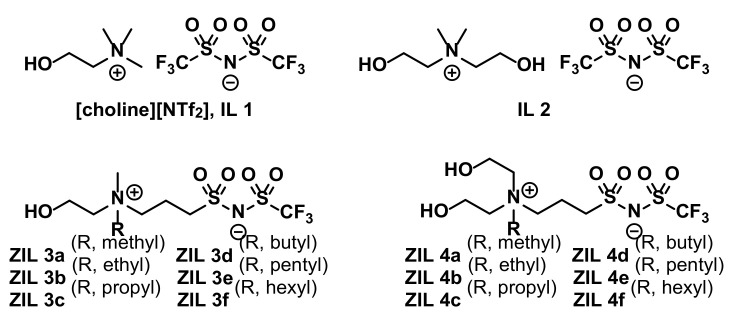
Structures of [NTf_2_]-based ionic liquids (**IL 1** and **IL 2**) and NTf-based zwitter-ionic liquids (**ZIL 3a**–**f** and **ZIL 4a**–**f**).

Since the *T_c_* value of **IL 1** was high and totally incompatible for use in studies involving proteins and other biomolecules, we accordingly argued that a structurally more hydrophilic, ‘choline-like’ **IL 2** should exhibit lower *T_c_* value, if present (Figure 1). In addition, we were greatly intrigued by the recent advances of ZIL materials developed by Ohno and coworkers [3,4,7,8] and therefore envisaged that, based on structures of **IL 1** and **IL 2**, *N*-triflimide (NTf)-based ionic liquids **ZIL 3a–f** and **ZIL 4a–f** are structurally tunable, zwitterionic, and non-volatile, and should be new candidate TILs for the structure-and-thermoresponsiveness (SAT) relationship study (Figure 1). Moreover, it is known from literature that natural and synthetic zwitterionic surfaces (e.g., mammalian cell-surface zwitterionic phosphorylcholine lipids and zwitterionic self-assembled monolayers, respectively) significantly reduce, if not totally eliminate, nonspecific protein adsorption [9,10,11,12] and the ZIL materials developed in this work will likely minimize partitioning interactions with proteins. For exactly these reasons, in this work we proceeded to prepare **IL 2** and set out to develop a synthesis of **ZIL 3a–f** and **ZIL 4a–f** with aims to discover new zwitterionic TILs exhibiting temperature-switchable phase separation with water and apply, as a proof-of-concept, for biomolecular interaction analysis.

### 2.1. Synthesis of **IL 2** and Zwitter-Ionic Liquids **ZIL 3a–f** and **ZIL 4a–f**

**Figure 2:**
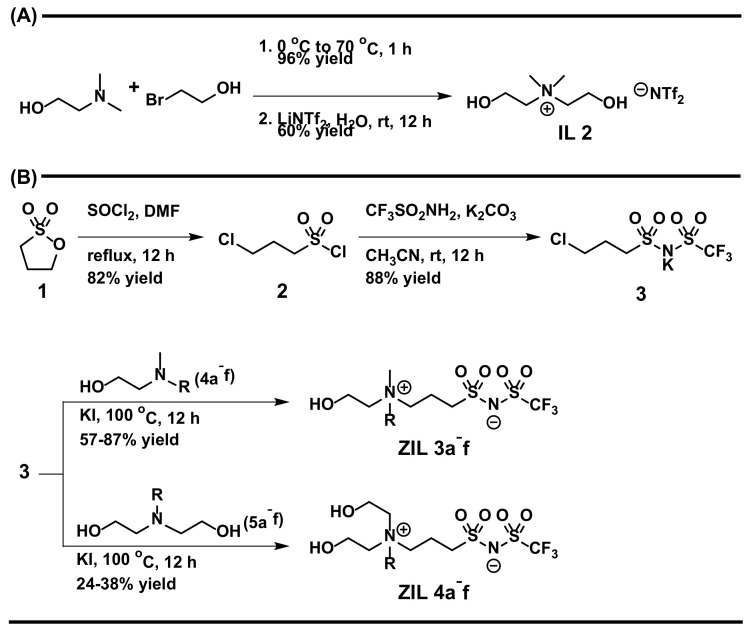
Synthesis of (**A**) **IL 2** and (**B**) **ZIL 3a–f** and **ZIL 4a–f** ionic liquids.

Figure 2A outlines a straightforward preparation of **IL 2** starting from commercial *N,N*-dimethylethanolamine. Figure 2B illustrates our synthesis of **ZIL 3a–f** and **ZIL 4a–f**, of which the key element **3** [13,14] could be readily prepared by the activation of an inexpensive 1,3-propanesultone **1** using thionyl chloride and DMF, leading to the formation of the sulfonyl chloride **2**, followed by its nucleophilic acyl substitution reaction with trifluoromethanesulfonamide under basic condition. This key intermediate **3** was afforded in grams scale with a high 72% isolated yield in two steps. We then finalized our synthesis of ZILs by *N*-alkylation of **3** with a series of tertiary amines (**4a–f** and **5a–f**), which could be readily prepared from reactions of commercial secondary amines (*N*-methylethanolamine for **4a–f** and diethanolamine for **5a–f**) with corresponding alkyl halides, to finally achieve the desired **ZIL 3a–f** and **ZIL 4a–f** ionic liquids. The overall yields, in our hands, for these 3-step syntheses of **ZIL 3a–f** and **ZIL 4a–f** were acceptable: 41–63% and 17–27%, respectively (Figure 2B). The overall low-yielding preparation of **ZIL 4a–f** was primarily due to the unwanted, but seemingly unavoidable, reaction participation of sidechain hydroxy groups on **5a**–**f** with **3** in the last step, resulting in reaction sluggishness and, therefore, tedious chromatographic separation and purification. Detailed ^1^H and ^13^C NMR, and high-resolution mass spectrometry (HRMS) spectra of all 14 ionic liquids (**IL 1**, **IL 2**, **ZIL 3a–f**, and **ZIL 4a–f**) are summarized in the Supporting Information (ESI).

### 2.2. Characterization of **IL 2**

**Figure 3:**
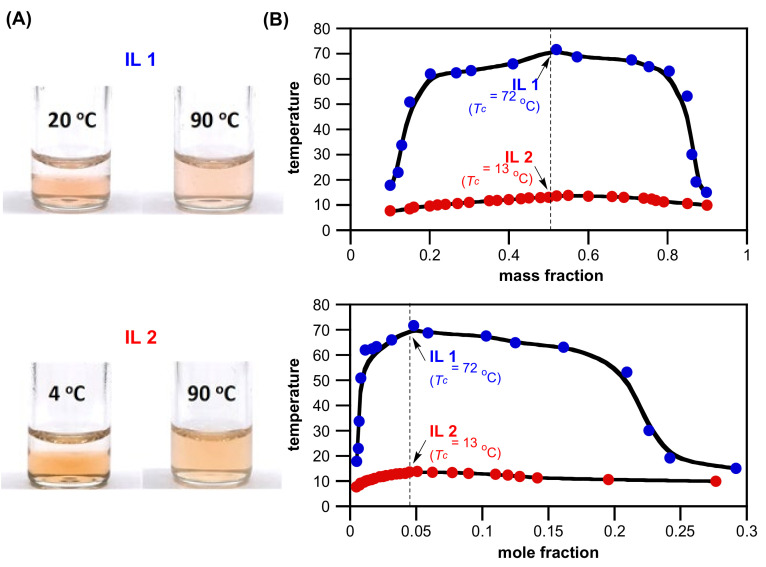
(**A**) Temperature-dependent phase transitions of binary mixtures (1:1, *w/w*) of water with **IL 1** (*T*_c_ = 72 °C) at 20 °C and 90 °C, and **IL 2** (*T*_c_ = 13 °C) at 4 °C and 90 °C, respectively. The Congo red dye (0.001 wt% in water) was added to make the phase boundary more noticeable. (**B**) Phase diagrams (both *T_c_* vs. mass fraction and *T_c_* vs. mole fraction) of a mixture of water with **IL 1** and **IL 2**, respectively. Solid lines are the guide for the eye.

Many ZILs reported in literature are solid at room temperature, primarily because of strong inter-electrostatic attractions among ZILs as well as hydrophobic interactions between sidechains on ZILs [3,4,6,7]. With thirteen final ionic products (**IL 2**, **ZIL 3a–f** and **ZIL 4a–f**) synthesized in this work, only **ZIL 3a** is an ionic salt with m.p. at 100 °C; other twelve are supercooled ionic liquids at room temperature [15,16] and viscous colorless-to-pale yellow liquid when obtained. As unambiguously demonstrated in Figure 3, we were pleased that **IL 2** not only was found as a room-temperature ionic liquid exhibiting UCST phase transition in water but also carried much lower *T_c_* value than that of **IL 1**: 13 °C and 72 °C at 1:1 mass ratio, respectively. **IL 2** forms a two-phase solution with water at 4 °C with ionic liquid in the bottom layer and, upon heating at or above room temperature, one-phase system is formed. This temperature-switchable phase separation is photographically illustrated in Figure 3A. For UCST systems, the phase transition temperatures highly depend on the mass (as well as mole) fraction of ionic liquids in water. Figure 3B shows phase diagrams of mixtures of water with **IL 1** (blue) and **IL 2** (red), respectively. As expected, both phase diagrams are of arch-shaped, convex curves with the highest critical temperatures near its mass ratio of 1:1 and mole ratio of 1:20 (**IL**/H_2_O), respectively.

### 2.3. Library Screening of **ZIL 3a–f** and **ZIL 4a–f**

**Figure 4:**
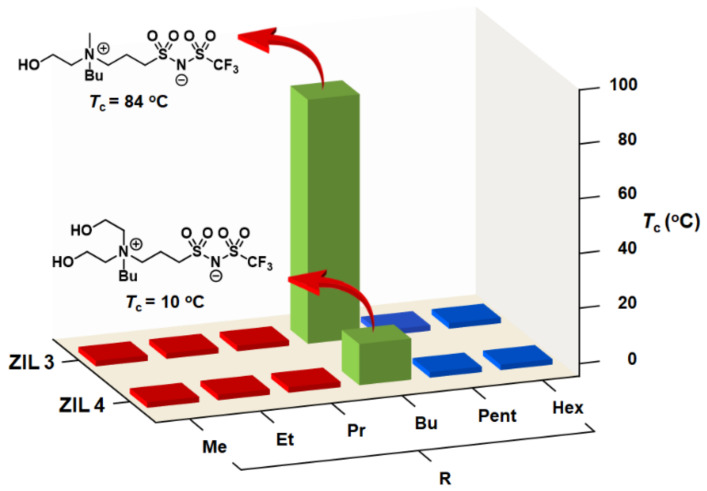
Phase transitions of a small library of 12 zwitterionic salts (**ZIL 3a–f** and **ZIL 4a–f**) upon mixing with water (1:1, *w/w*) at temperatures between 0 °C and 90 °C. Phase transition results shown in red and blue indicate an entirely homogeneous (one-phase) solution and a heterogeneous (two-phase) mixture, respectively, between 0 °C and 90 °C. In the library, two room-temperature ionic liquids show phase transitions: **ZIL 3d** (*T_c_* = 84 °C) and **ZIL 4d** (*T_c_* = 10 °C).

Figure 4 shows a small library of 12 NTf-based zwitterionic salts (**ZIL 3a–f** and **ZIL 4a–f**) and their phase behaviors toward temperature changes with water. To experimentally discover TILs, each zwitterionic salt was mixed with water in a mass ratio of 1:1 (*w/w*) and the mixture was then placed in a hot bath (90 °C) followed by gradual cooling until it reached 0 °C. The phase transition temperature (*T_c_*) for UCST was measured at a temperature point when the aqueous solution turned cloudy during cooling as observed by the naked eye. We were gratified that, among the 12 zwitterionic salts screened and tested, two ZILs (labeled in green) were found to exhibit UCST phase transitions: **ZIL 3d** and **ZIL 4d** with *T_c_* value of 84 °C and 10 °C at 1:1 mass ratio, respectively (Figure 4). The phase transition results from those zwitterionic salts labeled in red and blue (Figure 4) indicate an entirely homogeneous (one-phase) solution and a heterogeneous (two-phase) mixture between 0 °C and 90 °C, respectively.

Not surprisingly, the structurally more hydrophilic **ZIL 4d** exhibits lower *T_c_* value than that of **ZIL 3d** (Figure 4). Furthermore, it has been reported in literature [4,17,18] that the phase property of a TIL is a fine balance between hydrophobicity and hydrophilicity of ionic salts investigated, and our results concurred that, as shown in Figure 3, **ZIL 3d** and **ZIL 4d** were identified to situate on the rim between being totally hydrophilic (red) and totally hydrophobic (blue).

### 2.4. Characterization of **ZIL 3d** and **ZIL 4d**

**Figure 5:**
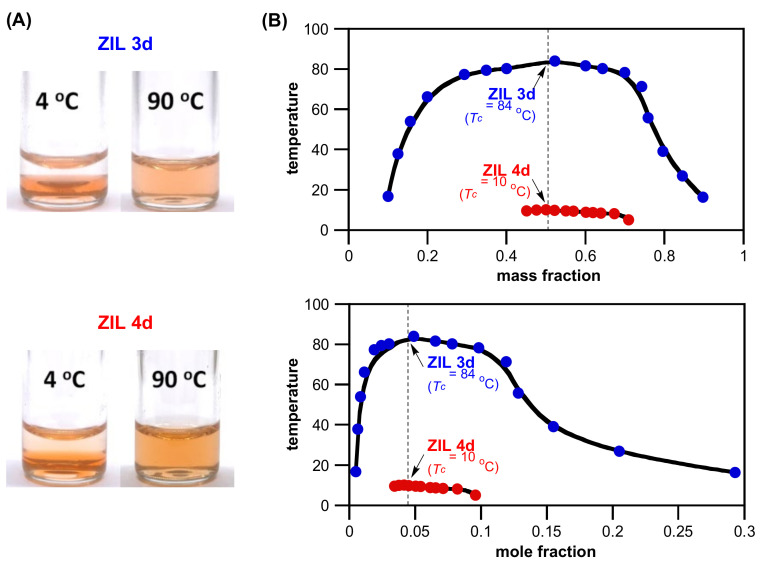
(**A**) Temperature-dependent phase transitions of binary mixtures (1:1, *w/w*) of water with **ZIL 3d** (*T*_c_ = 84 °C) and **ZIL 4d** (*T*_c_ = 10 °C) at 4 °C and 90 °C, respectively. The Congo red dye (0.001 wt% in water) was added to make the phase boundary more noticeable. (**B**) Phase diagrams (both *T_c_* vs. mass fraction and *T_c_* vs. mole fraction) of a mixture of water with **ZIL 3d** and **ZIL 4d**, respectively. Solid lines are the guide for the eye.

Figure 5A shows photos of phase behavior for **ZIL 3d** and **ZIL 4d**. This result clearly demonstrated that the replacement of an ammonium sidechain from the methyl group in **ZIL 3d** to a glycol group in **ZIL 4d** sharply increased the ZIL hydrophilicity and apparently lowered their phase transition temperatures from 84 °C to 10 °C. This abrupt decrease in *T_c_* value is evidently in line with the change of *T_c_* from 72 °C for **IL 1** to 13 °C for a more hydrophilic **IL 2** (Figure S1, Supplementary Materials). The phase diagrams of **ZIL 3d** and **ZIL 4d** are shown in Figure 5B, in which the reversible UCST of **ZIL 4d** is only experimentally observed at mass fraction between 41% and 71% in water. Unlike the **ZIL 3d** system, the observation of a complete arch-shaped UCST curve for **ZIL 4d** was limited experimentally due to the freezing temperature of water. Both phase diagrams are with the highest critical temperatures near its mass ratio of 1:1 and mole ratio of 1:21 (**IL**/H_2_O), respectively. This successful development of **ZIL 3d** and **ZIL 4d** as TILs clearly highlighted the real value of structural tunability of ionic liquids, and both **ZIL 3d** and **ZIL 4d** were two fruitful TIL examples using the SAT-based discovery platform developed in this work.

### 2.5. Discovery of a TIL from Two Non-Thermoresponsive **ZIL** Pairs

We investigated further to test whether a mixture of a hydrophilic ZIL and a neighboring hydrophobic ZIL as shown in Figure 4 might change their overall phase behavior in water [19]. As illustrated in Figure 4, the **ZIL 3c** is hydrophilic and totally homogeneous with water, but the **ZIL 3e** is hydrophobic and water immiscible, at temperatures between 4 °C and 90 °C; that is, both are not thermoresponsive toward temperature changes. Here, we clearly showed that a simple mixing of **ZIL 3c** and **ZIL 3e** with water is a convenient method to control total hydrophobicity toward phase transition and, with this example, an equal mass mixture of **ZIL 3c** and **ZIL 3e** in water readily formed a phase separation with a *T_c_* at 81 °C (Figure S2, Supplementary Materials). In addition, this aqueous mixture returned to a two-phase solution when cooled to 23 °C, confirming the successful development of a three-component UCST system. This simple platform opens the possibility for use with a wide range of zwitterionic salts for the discovery of ZIL pairs as TIL.

### 2.6. Rational Structural Engineering and Optimization of **ZIL 3d** and **ZIL 4d**

**Figure 6:**
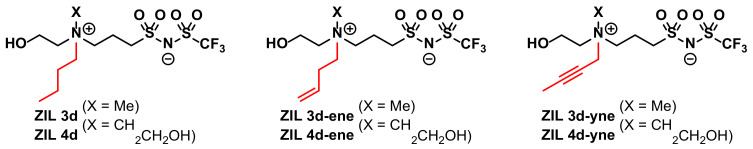
Structures of sidechain-engineered zwitter-ionic liquids: **ZIL 3d-ene**, **ZIL 3d-yne**, **ZIL 4d-ene**, and **ZIL 4d-yne**.

Because *T_c_* values of **ZIL 3d** (*T*_c_ = 84 °C) and **ZIL 4d** (*T*_c_ = 10 °C) obtained are distant from body temperature (37 °C), both ZILs are incompatible for direct biochemical studies involving biomolecules such as proteins. We foresaw that further structural fine-tuning, by replacing the less polar, sp^3^-hybridized butyl (Bu) group with a slightly more polar sp^2^-hybridized 1-butenyl or sp-hybridized 2-butynyl substituents carrying both greater electronegative s-characters and more polarizable π-bond electrons, resulting in the incorporation of larger permanent dipole moments (for example, 0.00–0.08, 0.30–0.50, 0.70–0.86 D for alkyl, alkenyl, and alkynyl groups, respectively [20,21]), should exhibit lower *T_c_* value with water, when present (Figure 6). Accordingly, we proceeded to synthesize **ZIL 3d-ene**, **ZIL 3d-yne**, **ZIL 4d-ene**, and **ZIL 4d-yne** with the hope of discovering new zwitterionic TILs carrying biocompatible *T_c_* values. All four newly engineered ZILs were prepared using the same synthetic route as illustrated in Figure 2. These **ZIL 3d-ene**, **ZIL 3d-yne**, **ZIL 4d-ene**, and **ZIL 4d-yne** ultimately afforded acceptable isolated yields: 53%, 58%, 25%, and 16%, respectively. Gratifyingly, these four new ZILs are all liquidous once obtained at room temperature.

### 2.7. Characterization of **ZIL 3d-ene** and **ZIL 3d-yne**

**Figure 7:**
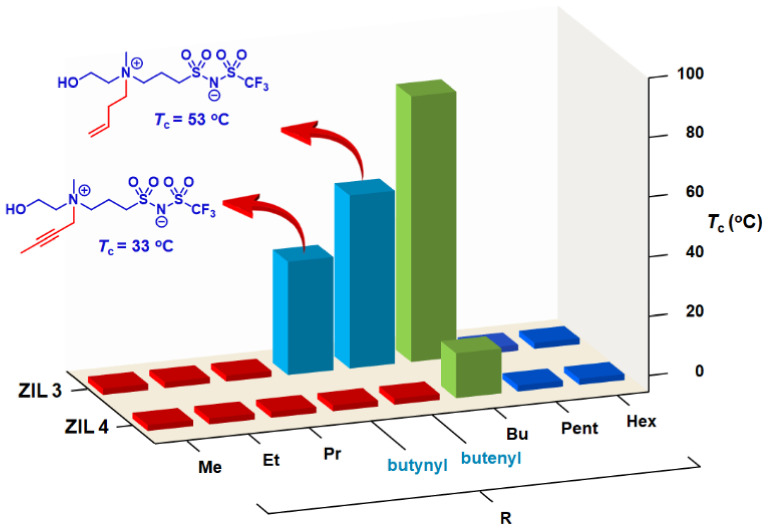
Phase transitions of **ZIL 3d-ene**, **ZIL 3d-yne**, **ZIL 4d-ene** and **ZIL 4d-yne**, along with **ZIL 3a**–**f** and **ZIL 4a–f**, upon mixing with water (1:1, *w/w*) at temperatures between 0 °C and 90 °C. Phase transition results shown in red and blue indicate an entirely homogeneous (one-phase) solution and a heterogeneous (two-phase) mixture, respectively, between 0 °C and 90 °C. Two additional room-temperature ZILs (labeled in sky blue) show phase transitions: **ZIL 3d-ene** (*T_c_* = 53 °C) and **ZIL 3d-yne** (*T_c_* = 33 °C).

We were pleased that, among four new ZILs tested, two were discovered having UCST phase transition with lower *T*_c_ values: **ZIL 3d-ene** and **ZIL 3d-yne** (Figure 7). Due to its already low *T*_c_ value inherent in **ZIL 4d** (*T*_c_ = 10 °C), both **ZIL 4d-ene** and **ZIL 4d-yne** were more hydrophilic than that of **ZIL 4d** and, therefore, completely miscible with water at temperatures between 0 °C and 90 °C. Satisfyingly, both **ZIL 3d-ene** and **ZIL 3d-yne**, labeled in sky blue (Figure 7), were found to carry *T_c_* values exhibiting lower *T*_c_ values than that of **ZIL 3d** (*T*_c_ = 84 °C). It is worth highlighting that the *T*_c_ value of **ZIL 3d-yne** (33 °C) is below body temperature and should then be our candidate ZIL for biomolecular recognition study with proteins.

### 2.8. A Proof-of-Concept Application of **ZIL 3d-yne** Used for Biomolecular Interaction Analysis

**Figure 8:**
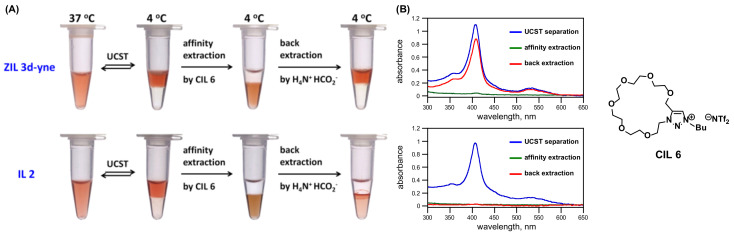
(**A**) Optical photographic images and (**B**) UV-vis spectra of thermoresponsive phase separation were exploited for demonstrating affinity extraction of protein by **CIL 6** (163 mM) from its aqueous mixtures (1:1, *w*/*w*) of **ZIL 3d-yne** with a solution of equine heart cytochrome c (0.3 mM). After affinity extraction, competitive extraction of cytochrome c in bottom ionic liquid layer into upper aqueous phase could be readily achieved using ammonium formate (0.5 M). Efficiencies of extractions of cytochrome c were quantitatively measured in upper aqueous layers using the Soret band at 410 nm.

As a proof-of-concept application, we next turned our attention to undertake its use for a preliminary biomolecular recognition study with the aim that, upon temperature-triggered phase separation, proteins would preferentially partition in aqueous layer, which would ultimately make biomolecular interaction analysis possible after adding an affinity ionic liquid that selectively extracts the target protein favorably into the ionic liquid phase. In this preliminary work, we selected cytochrome c from equine heart for its ease of visualization by naked eyes and straightforward quantitative measurements by UV-vis spectrophotometer to test affinity extraction in both zwitterionic **ZIL 3d-yne** and non-zwitterionic **IL 2** TIL systems using the crowned ionic liquid **CIL 6** previously developed in this laboratory [22]. This **CIL 6** is capable of binding with arginine- and lysine-containing peptides and proteins [22]. We were pleased that, as shown in Figure 8, cytochrome c having 19 Lys and 2 Arg residues in its sequence [23] was indeed found predominantly partitioned in the upper aqueous layer and could then be readily affinity extracted into the bottom **ZIL 3d-yne** and also **IL 2** phase after introducing **CIL 6** in the system. This cytochrome c in the **ZIL 3d-yne** ionic liquid layer was smoothly and competitively extracted back to the upper aqueous phase upon the treatment of 0.5 M ammonium formate (Figure 8A). However, back extraction of cytochrome c in the bottom **IL 2** layer into the aqueous phase by ammonium formate, in our hands, was completely unsuccessful and found notably concentrated at the interface between two layers (Figure 8A). This preliminary result observed in **IL 2** system was seemingly due to the occurrence of undesired ion exchanges in cytochrome c, leading to its immiscibility in aqueous phase. The results shown in Figure 8A clearly highlighted the importance of the use of ZILs for protein interaction analysis. From spectra shown in Figure 8B, upon thermoresponsive phase separation, the upper aqueous phases (blue spectra for both **ZIL 3d-yne** and **IL 2**) contained essentially all of cytochrome c and, when **CIL 6** was introduced, most, if not all, of protein was affinity extracted into ionic liquid-rich bottom phase (green spectra). In **ZIL 3d-yne** system, the recovery yield of cytochrome c by ammonium formate was high: 81% (red spectrum). A similar result of the recognition-based extraction using NTf- and oligoglyme-conjugated ZILs as small-molecule TILs that exhibit LCST, instead of UCST, phase transition in water was developed most recently in our laboratory showing promising application in affinity separation of biomolecules [24].

## 3. Materials and Methods

### 3.1. General Information

The ^1^H-NMR, ^13^C-NMR and ^19^F-NMR spectra were recorded at 400 MHz, 100 MHz and 376 MHz, respectively, on a Bruker AVANCEIII HD 400 NMR spectrometer (Bruker BioSpin GmbH, Rheinstetten, Germany) in deuterated solvents (D_2_O and DMSO-d_6_). The chemical shift (δ) for ^1^H-NMR, ^13^C-NMR and ^19^F-NMR are given in ppm relative to the residual signal of the solvent. Coupling constants are given in Hz. The following abbreviations are used to indicate the multiplicity: s, singlet; d, doublet; t, triplet; q, quartet; quin, quintet; m, multiplet; dd, doublet of doublets; td, triplet of doublets; dt, doublet of triplets; ddd, doublet of doublet of doublets; bs, broad signal. The reactions were monitored using TLC (thin-layer chromatography) silica gel 60 F254 (Merck KGaA, Darmstadt, Germany). Evaporation of solvents was performed under reduced pressure. Melting points were measured and recorded by the OptiMelt MPA-100 apparatus (Standford Research Systems, Sunnyvale, CA, USA) and uncorrected.

### 3.2. Synthesis and Characterization of Ionic Liquids

#### 3.2.1. Synthesis of **IL 1**

To a solution of choline chloride (2 g, 14.39 mmol) in deionized water (2 mL) was added LiNTf_2_ (4.57 g, 15.83 mmol). The solution was stirred and allowed to undergo ion exchange at room temperature for 12 h. After this time, the mixture solution was extracted three times with CH_2_Cl_2_. The combined organic layers were dried with anhydrous sodium sulfate, filtered, and concentrated to afford colorless ionic liquid **IL 1**, [choline][NTf_2_] (5.53 g, 100% yield) [6].

#### 3.2.2. Synthesis of **IL 2**

To a round bottle containing 2-(dimethylamino)ethanol (1 g, 11.21 mmol) was added dropwise 2-bromoethanol (12.3 mmol) under ice bath. Accordingly, the alkylation reaction was carried out at 70 °C for 1 h. After the completion of reaction, reaction mixture became a wax solid. The residue was smashed and washed with ether, then the solvent was removed under reduced pressure to obtain the product, [N_1,1,(EtOH)2_][Br], as white solid (2.317, 96% yield).

To a solution of [N_1,1,(EtOH)2_][Br] (0.75 g, 3.48 mmol) in deionized water (2 mL) was added LiNTf_2_ (1.169 g, 4.07 mmol). The solution was stirred and allowed to undergo ion exchange at room temperature for 12 h. After this time, the mixture solution was extracted three times with CH_2_Cl_2_. The combined organic layers were dried with anhydrous sodium sulfate, filtered, and concentrated to afford colorless ionic liquid **IL 2**, [N_1,1,(EtOH)2_][NTf_2_] (0.88 g, 60% yield).

^1^H NMR (400 MHz, D_2_O) δ 2.23 (s, 2 × N^+^C*H*_3_, 6H), 3.56–3.63 (m, 2 × HOCH_2_C*H*_2_N^+^, 2H), 4.02–4.13 (m, 2 × HOC*H*_2_CH_2_N^+^, 2H); ^13^C NMR (100 MHz, DMSO-*d*_6_) δ 51.62, 55.01, 65.85, 129.50 (q, *J*_CF_ = 320 Hz); ESI-HRMS *m*/*z* [M]^+^ calculated for C_6_H_16_NO_2_ 134.1176, found 134.1179.

### 3.3. Synthesis of ZILs

#### 3.3.1. Synthesis of 3-Chloropropane-1-Sulfonyl Chloride (**2**)

To a round-bottom flask containing 1,3-propanesultone **1** (3 g, 24.7 mmol) was added thionyl chloride (3.6 mL, 49.5 mmol) and DMF (0.2 mL). The mixture was refluxed for 12 h. The excess thionyl chloride was removed by vacuum to obtain sulfonyl chloride product as a pale yellow liquid **2** (3.58 g, 82% yield).

#### 3.3.2. Synthesis of Potassium ((3-Chloropropyl)Sulfonyl)((Trifluoromethyl)Sulfonyl)Amide (**3**)

To a bottle containing trifluoromethanesulfonimide (1.0 g, 6.78 mmol) and K_2_CO_3_ (1.39 g, 10.18 mmol) was added acetonitrile (28 mL) and stirred at room temperature. Sulfonyl chloride product **2** (1.40 g, 7.5 mmol) was dissolved in acetonitrile (5 mL) and added dropwise to the reaction bottle. After 12 h, the solid salt was filtered off. The filtrate was concentrated under reduced pressure to obtain yellow solid crude product. Then, the crude product was washed with mixture solvent of EA and DCM (1/2) to afford white solid product **3** (1.95 g, 88% yield).

#### 3.3.3. Synthesis of **ZIL 3a**

To a screw-cap septum vial containing potassium sulfonimide **3** (400 mg, 1.22 mmol) was added 2-(dimethylamino)ethanol **4a** (5 equiv). The mixture was heated at 100 °C for 12 h. After completion of the reaction, acetonitrile was added to the vial for precipitation of potassium chloride produced. The solid salt was filtered off, and the filtrate was concentrated under reduced pressure to obtain yellow liquid crude product. Next the excess amine of crude product was washed out with sonication in ether. Then, the mixture solution of EA and MeOH (5/1, *v*/*v*) was added to the bottle containing crude product. White solid will precipitate in solvent. After collecting the white solid and washing it with EA several times, residual solvent was removed in vacuo to obtain **ZIL 3a** as white solid (295 mg, 70% yield).

White solid, m.p. 100 °C; ^1^H NMR (400 MHz, DMSO-*d*_6_) δ 2.05–2.16 (m, NCH_2_C*H*_2_, 2H), 3.05 (t, *J* = 7.2 Hz, CH_2_C*H*_2_S, 2H), 3.07 (s, 2 × N^+^CH_3_, 6H), 3.92 (t, *J* = 5.2 Hz, N^+^C*H*_2_CH_2_, 2H), 3.42–3.50 (m, HOCH_2_C*H*_2_, 2H), 3.78–3.86 (m, HOC*H*_2_CH_2_, 2H), 3.32 (bs, *H*OCH_2_CH_2_, H); ^13^C NMR (100 MHz, DMSO-*d*_6_) δ 17.69, 50.96, 51.22, 54.85, 62.31, 64.72; ^19^F NMR (376 MHz, DMSO-*d*_6_) δ −76.53 (CF_3_, 3F); ESI-HRMS *m*/*z* [M + H]^+^ calculated for C_8_H_18_F_3_N_2_O_5_S_2_ 343.0604, found 343.0594 ([M + H]^+^), 365.0415 ([M + Na]^+^), 381.0155 ([M + K]^+^).

#### 3.3.4. Synthesis of **ZIL 3b–f**, **3d-ene**, and **3d-yne**

To a screw cap septum vial containing potassium sulfonimide product **3** (200 mg, 0.613 mmol) and potassium iodide (20 mg, 0.2 equiv) was added tertiary amine **4b**–**f** (for **ZIL 3b–f**), *N*-(but-3-enyl)-*N*-methylaminoethan-1-ol (for **ZIL 3d-ene**), or *N*-(but-2-ynyl)-N-methylaminoethan-1-ol (for **ZIL 3d-yne**) (3 equiv). The mixture was heated at 100 °C for 12 h. After completion of reaction, acetonitrile was added to the vial for precipitation of potassium chloride. The solid salt was filtered off, and filtrate was concentrated under reduced pressure to obtain yellow liquid crude product. Next the excess amine of crude product was washed out with sonication in ether. The crude product was dissolved in a mixture solution of EA and MeOH (5/1, 30 mL), then was poured into a bottle containing ether (150 mL). The mixture solution will become white turbid solution. After centrifugation of the white turbid solution, pale yellow ZIL will precipitate in the solvent. After collecting the precipitate and washing it with ether several times, residual was purified by silica gel column chromatography (ethyl acetate/methanol = 5/1) to afford pale-yellow liquid.

**ZIL 3b** pale yellow liquid (87% yield); ^1^H NMR (400 MHz, DMSO-*d*_6_) δ 1.24 (t, *J* = 7.0 Hz, N^+^CH_2_C*H*_3_, 3H), 2.02–2.16 (m, N^+^CH_2_C*H*_2_CH_2_S, 2H), 3.02 (s, N^+^CH_3_, 3H), 3.07 (t, *J* = 7.2 Hz, CH_2_C*H*_2_S, 2H), 3.32–3.50 (m, N^+^C*H*_2_CH_2_ + N^+^C*H*_2_CH_3_ + HOCH_2_C*H*_2_, 6H), 3.76–3.87 (m, HOC*H*_2_CH_2_, 2H), 5.28 (t, *J* = 4.8 Hz, *H*OCH_2_CH_2_, 1H); ^13^C NMR (100 MHz, DMSO-*d*_6_) δ 7.53, 17.35, 47.87, 51.17, 54.68, 57.29, 59.02, 62.06, 120.06 (q, *J*_CF_ = 322 Hz); ^19^F NMR (376 MHz, DMSO-*d*_6_) δ −76.56 (CF_3_, 3F); ESI-HRMS m/z [M + H]^+^ calculated for C_9_H_20_F_3_N_2_O_5_S_2_ 357.0760, found 357.0757 ([M + H]^+^), 379.0581 ([M + Na]^+^).

**ZIL 3c** pale yellow liquid (68% yield); ^1^H NMR (400 MHz, DMSO-*d*_6_) δ 0.88 (t, *J* = 7.2 Hz, N^+^CH_2_ CH_2_C*H*_3_, 3H), 1.62–1.73 (m, N^+^CH_2_C*H*_2_CH_3_, 2H), 2.03–2.16 (m, N^+^CH_2_C*H*_2_CH_2_S, 2H), 3.03 (s, N^+^CH_3_, 3H), 3.06 (t, *J* = 7.2 Hz, CH_2_C*H*_2_S, 2H), 3.23–3.32 (m, N^+^C*H*_2_CH_2_CH_3_, 2H), 3.35–3.41 (m, N^+^C*H*_2_CH_2_CH_2_S, 2H), 3.40–3.48 (m, HOCH_2_C*H*_2_, 2H), 3.77–3.86 (m, HOC*H*_2_CH_2_, 2H), 5.35 (bs, *H*OCH_2_CH_2_, 1H); ^13^C NMR (100 MHz, DMSO-*d*_6_) δ 10.40, 15.04, 17.39, 48.36, 51.12, 54.62, 59.62, 62.62, 62.99, 120.03 (q, *J*_CF_ = 322 Hz); ^19^F NMR (376 MHz, DMSO-*d*_6_) δ −76.57 (CF_3_, 3F); ESI-HRMS *m*/*z* [M + H]^+^ calculated for C_10_H_22_F_3_N_2_O_5_S_2_ 371.0917, found 371.0918 ([M + H]^+^), 393.0739 ([M + Na]^+^), 409.0479 ([M + K]^+^).

**ZIL 3d** pale yellow liquid (64% yield); ^1^H NMR (400 MHz, DMSO-*d*_6_) δ 0.92 (t, *J* = 7.2 Hz, N^+^CH_2_CH_2_CH_2_C*H*_3_, 3H), 1.21–1.37 (m, N^+^CH_2_CH_2_C*H*_2_CH_3_, 2H), 1.57–1.72 (m, N^+^CH_2_C*H*_2_CH_2_CH_3_, 2H), 2.02–2.16 (m, N^+^CH_2_C*H*_2_CH_2_S, 2H), 3.03 (s, N^+^CH_3_, 3H), 3.06 (t, *J* = 7.2 Hz, CH_2_C*H*_2_S, 2H), 3.26–3.34 (m, N^+^C*H*_2_CH_2_CH_2_CH_3_, 2H), 3.34–3.40 (m, N^+^C*H*_2_CH_2_CH_2_S, 2H), 3.40–3.48 (m, HOCH_2_C*H*_2_, 2H), 3.77–3.85 (m, HOC*H*_2_CH_2_, 2H), 5.26 (t, *J* = 5.2 Hz, *H*OCH_2_CH_2_, 1H); ^13^C NMR (100 MHz, DMSO-*d*_6_) δ 13.43, 17.42, 19.16, 23.34, 48.40, 51.13, 54.68, 59.55, 61.48, 62.62, 120.04 (q, *J*_CF_ = 323 Hz); ^19^F NMR (376 MHz, DMSO-*d*_6_) δ −76.59 (CF_3_, 3F); ESI-HRMS *m*/*z* [M + H]^+^ calculated for C_11_H_24_F_3_N_2_O_5_S_2_ 385.1073, found 385.1075 ([M + H]^+^), 407.0895 ([M + Na]^+^).

**ZIL 3d-ene** pale yellow liquid (73% yield); ^1^H NMR (400 MHz, DMSO-*d*_6_) δ 2.03–2.19 (m, N^+^CH_2_C*H*_2_CH_2_S, 2H), 2.42–2.59 (m, N^+^CH_2_C*H*_2_CH = CH_2_, 2H), 3.02–3.12 (m, CH_2_C*H*_2_S, 2H), 3.06 (s, N^+^CH_3_, 3H), 3.26–3.34 (m, N^+^C*H*_2_CH_2_CH = CH_2_ + N^+^C*H*_2_CH_2_CH_2_S, 2H + HOCH_2_C*H*_2_, 6H), 3.77–3.88 (m, HOC*H*_2_CH_2_, 2H), 5.10–5.27 (m, N^+^CH_2_CH_2_CH = C*H*_2_, 2H), 5.30 (t, *J* = 4.4 Hz, *H*OCH_2_CH_2_, 1H), 5.66–5.81 (m, N^+^CH_2_CH_2_C*H* = CH_2_, 1H); ^13^C NMR (100 MHz, DMSO-*d*_6_) δ 17.41, 26.08, 48.42, 51.08, 54.68, 59.60, 60.42, 62.72, 118.40, 120.02 (q, *J*_CF_ = 323 Hz); ^19^F NMR (376 MHz, DMSO-*d*_6_) δ −76.54 (CF_3_, 3F); ESI-HRMS *m*/*z* [M + H]^+^ calculated for C_11_H_22_F_3_N_2_O_5_S_2_ 383.0917, found 383.0911 ([M + H]^+^), 405.0729 ([M + Na]^+^).

**ZIL 3d-yne** pale yellow liquid (80% yield); ^1^H NMR (400 MHz, DMSO-*d*_6_) δ 1.93 (s, N^+^CH_2_C ≡ CC*H*_3_, 3H), 2.06–2.19 (m, N^+^CH_2_C*H*_2_CH_2_S, 2H), 3.03–3.15 (m, CH_2_C*H*_2_S, 2H), 3.08 (s, N^+^CH_3_, 3H), 3.41–3.48 (m, N^+^C*H*_2_CH_2_CH_2_S, 2H), 3.48–3.60 (m, HOCH_2_C*H*_2_, 2H), 3.78–3.89 (m, HOC*H*_2_CH_2_, 2H), 4.30–4.39 (m, N^+^C*H*_2_C ≡ CCH_3_, 2H), 5.33 (t, *J* = 4.8 Hz, *H*OCH_2_CH_2_, 1H); ^13^C NMR (100 MHz, DMSO-*d*_6_) δ 3.34, 17.55, 48.23, 51.19, 53.11, 54.69, 59.75, 62.29, 67.46, 88.77, 120.04 (q, *J*_CF_ = 322 Hz); ^19^F NMR (376 MHz, DMSO-*d*_6_) δ −76.52 (CF_3_, 3F); ESI-HRMS *m*/*z* [M + H]^+^ calculated for C_11_H_20_F_3_N_2_O_5_S_2_ 381.0760, found 381.0765 ([M + H]^+^), 403.0581 ([M + Na]^+^).

**ZIL 3e** pale yellow liquid (57% yield); ^1^H NMR (400 MHz, DMSO-*d*_6_) δ 0.89 (t, *J* = 7.0 Hz, N^+^CH_2_CH_2_(CH_2_)_2_C*H*_3_, 3H), 1.18–1.40 (m, N^+^CH_2_CH_2_(C*H*_2_)_2_CH_3_, 4H), 1.59–1.74 (m, N^+^CH_2_C*H*_2_(CH_2_)_2_CH_3_, 2H), 2.02–2.16 (m, N^+^CH_2_C*H*_2_CH_2_S, 2H), 3.03 (s, N^+^CH_3_, 3H), 3.06 (t, *J* = 6.8 Hz, CH_2_C*H*_2_S, 2H), 3.25–3.33 (m, N^+^C*H*_2_CH_2_(CH_2_)_2_CH_3_, 2H), 3.33–3.50 (m, N^+^C*H*_2_CH_2_CH_2_S + HOCH_2_C*H*_2_, 4H), 3.76–3.86 (m, HOC*H*_2_CH_2_, 2H), 5.27 (t, *J* = 4.8 Hz, *H*OCH_2_CH_2_, 1H); ^13^C NMR (100 MHz, DMSO-*d*_6_) δ 13.71, 17.42, 21.07, 21.61, 27.88, 48.37, 51.15, 54.69, 59.57, 61.70, 62.61, 120.05 (q, *J*_CF_ = 322 Hz); ^19^F NMR (376 MHz, DMSO-*d*_6_) δ −76.57 (CF_3_, 3F); ESI-HRMS *m*/*z* [M + Na]^+^ calculated for C_12_H_25_F_3_N_2_NaO_5_S_2_ 421.1049, found 421.1044.

**ZIL 3f** pale yellow liquid (58% yield); ^1^H NMR (400 MHz, DMSO-*d*_6_) δ 0.87 (t, *J* = 6.8 Hz, N^+^CH_2_CH_2_(CH_2_)_3_C*H*_3_, 3H), 1.17–1.37 (m, N^+^CH_2_CH_2_(C*H*_2_)_3_CH_3_, 4H), 1.55–1.73 (m, N^+^CH_2_C*H*_2_(CH_2_)_3_CH_3_, 2H), 1.96–2.17 (m, N^+^CH_2_C*H*_2_CH_2_S, 2H), 3.03 (s, N^+^CH_3_, 3H), 3.06 (t, *J* = 6.8 Hz, CH_2_C*H*_2_S, 2H), 3.25–3.33 (m, N^+^C*H*_2_CH_2_(CH_2_)_3_CH_3_, 2H), 3.33–3.49 (m, N^+^C*H*_2_CH_2_CH_2_S + HOCH_2_C*H*_2_, 4H), 3.75–3.87 (m, HOC*H*_2_CH_2_, 2H), 5.26 (t, *J* = 4.8 Hz, *H*OCH_2_CH_2_, 1H); ^13^C NMR (100 MHz, DMSO-*d*_6_) δ 13.84, 17.46, 21.36, 21.92, 25.46, 30.66, 48.41, 51.18, 54.73, 59.59, 61.74, 62.64, 120.09 (q, *J*_CF_ = 322 Hz); ^19^F NMR (376 MHz, DMSO-*d*_6_) δ −76.58 (CF_3_, 3F); ESI-HRMS *m*/*z* [M + H]^+^ calculated for C_13_H_28_F_3_N_2_O_5_S_2_ 413.1386, found 413.1386 ([M + H]^+^), 435.1208 ([M + Na]^+^), 451.0948 ([M + K]^+^).

#### 3.3.5. Synthesis of **ZIL 4a**

To a screw cap septum vial containing potassium sulfonimide product **3** (200 mg, 0.613 mmol) and potassium iodide (20 mg, 0.2 equiv) was added tertiary amine **5a** (3 equiv). The mixture was heated at 100 °C for 12 h. After completion of the reaction, acetonitrile was added to the vial for precipitation of potassium chloride. The solid salt was filtered off, and filtrate was concentrated under reduced pressure to obtain yellow liquid crude product. Next the excess amine of crude product was washed out with sonication in ether. Then, the mixture solution of EA and MeOH (5/1) was added to bottle containing crude product. The mixture solution will become white turbid solution. After centrifugation of the white turbid solution, pale-yellow ZIL will precipitate in the solvent. After collecting the precipitate and washing it with ether several times, residual solvent was removed in vacuo to obtain **ZIL 4a** as pale-yellow liquid (80 mg, 35% yield).

Pale-yellow liquid; ^1^H NMR (400 MHz, DMSO-*d*_6_) δ 2.05–2.18 (m, NCH_2_C*H*_2_, 2H), 3.05 (t, *J* = 7.2 Hz, CH_2_C*H*_2_S, 2H), 3.10 (s, N^+^CH_3_, 3H), 3.41–3.55 (m, NC*H*_2_CH_2_ + 2 × HOCH_2_C*H*_2_, 6H), 3.77–3.87 (m, 2 × HOC*H*_2_CH_2_, 4H), 3.32 (t, *J* = 4.8 Hz, 2 × *H*OCH_2_CH_2_, 2H); ^13^C NMR (100 MHz, DMSO-*d*_6_) δ 17.48, 49.17, 51.23, 54.71, 60.49, 63.41, 120.05 (q, *J*_CF_ = 322 Hz); ^19^F NMR (376 MHz, DMSO-*d*_6_) δ −76.52 (CF_3_, 3F); ESI-HRMS *m*/*z* [M + H]^+^ calculated for C_9_H_20_F_3_N_2_O_6_S_2_ 373.0709, found 373.0707 ([M + H]^+^), 395.0532 ([M + Na]^+^).

#### 3.3.6. Synthesis of **ZIL 4b–f**, **4d-ene**, and **4d-yne**

To a screw cap septum vial containing potassium sulfonimide product **3** (200 mg, 0.613 mmol) and potassium iodide (20 mg, 0.2 equiv) was added tertiary amine **5b–f** (for **ZIL 4b–f**), *N*-(but-3-enyl)-*N*-methylaminoethan-1-ol (for **ZIL 4d-ene**), or *N*-(but-2-ynyl)-*N*-methylaminoethan-1-ol (for **ZIL 4d-yne**) (3 equiv). The mixture was heated at 100 °C for 12 h. After completion of reaction, acetonitrile was added to the vial for precipitation of potassium chloride. The solid salt was filtered off, and filtrate was concentrated under reduced pressure to obtain yellow liquid crude product. Next the excess amine of crude product was washed out with sonication in ether. The crude product was dissolved in a mixture solution of EA and MeOH (5/1, 30 mL), then was poured into a bottle containing ether (150 mL). The mixture solution will become white turbid solution. After centrifugation of the white turbid solution, pale-yellow ZIL will precipitate in the solvent. After collecting the precipitate and washing it with ether several times, the crude ZIL product was purified by silica gel column chromatography (ethyl acetate/methanol = 5/1) to afford pale yellow liquid.

**ZIL 4b** pale yellow liquid (32% yield); ^1^H NMR (400 MHz, DMSO-*d*_6_) δ 1.22 (t, *J* = 6.4 Hz, N^+^CH_2_C*H*_3_, 3H), 2.00–2.15 (m, N^+^CH_2_C*H*_2_CH_2_S, 2H), 3.06 (t, *J* = 6.8 Hz, CH_2_C*H*_2_S, 2H), 3.38-3.51 (m, N^+^C*H*_2_CH_3_ + N^+^C*H*_2_CH_2_CH_2_S + 2 × HOCH_2_C*H*_2_, 8H), 3.75–3.87 (m, 2 × HOC*H*_2_CH_2_, 4H), 5.26 (t, *J* = 4.8 Hz, *H*OCH_2_CH_2_, 1H); ^13^C NMR (100 MHz, DMSO-*d*_6_) δ 7.40, 17.09, 51.14, 54.54, 55.02, 56.90, 59.66, 61.70, 62.61, 120.08 (q, *J*_CF_ = 322 Hz); ^19^F NMR (376 MHz, DMSO-*d*_6_) δ −76.54 (CF_3_, 3F); ESI-HRMS *m*/*z* [M + H]^+^ calculated for C_10_H_22_F_3_N_2_O_6_S_2_ 387.0866, found 387.0860 ([M + H]^+^), 409.0678 ([M + Na]^+^).

**ZIL 4c** pale yellow liquid (24% yield); ^1^H NMR (400 MHz, DMSO-*d*_6_) δ 0.87 (t, *J* = 7.2 Hz, N^+^CH_2_CH_2_C*H*_3_, 3H), 1.60–1.73 (m, N^+^CH_2_C*H*_2_CH_3_, 2H), 2.02–2.14 (m, N^+^CH_2_C*H*_2_CH_2_S, 2H), 3.06 (t, *J* = 7.2 Hz, CH_2_C*H*_2_S, 2H), 3.27–3.38 (m, N^+^C*H*_2_CH_2_CH_3_, 2H), 3.38–3.55 (m, N^+^C*H*_2_CH_2_CH_2_S + 2 × HOCH_2_C*H*_2_, 6H), 3.74–3.86 (m, 2 × HOC*H*_2_CH_2_, 4H), 5.25 (t, *J* = 4.8 Hz, *H*OCH_2_CH_2_, 1H); ^13^C NMR (100 MHz, DMSO-*d*_6_) δ 10.79, 15.28, 17.59, 51.57, 54.98, 57.86, 60.68, 61.26, 120.51 (q, *J*_CF_ = 322 Hz); ^19^F NMR (376 MHz, DMSO-*d*_6_) δ −76.57 (CF_3_, 3F); ESI-HRMS *m*/*z* [M + H]^+^ calculated for C_11_H_24_F_3_N_2_O_6_S_2_ 401.0122, found 401.1017 ([M + H]^+^), 423.0835 ([M + Na]^+^).

**ZIL 4d** pale yellow liquid (34% yield); ^1^H NMR (400 MHz, DMSO-*d*_6_) δ 0.92 (t, *J* = 7.2 Hz, N^+^CH_2_CH_2_CH_2_C*H*_3_, 3H), 1.21–1.37 (m, N^+^CH_2_CH_2_C*H*_2_CH_3_, 2H), 1.58–1.72 (m, N^+^CH_2_C*H*_2_CH_2_CH_3_, 2H), 2.02–2.16 (m, N^+^CH_2_C*H*_2_CH_2_S, 2H), 3.06 (t, *J* = 6.4 Hz, CH_2_C*H*_2_S, 2H), 3.30–3.40 (m, N^+^C*H*_2_CH_2_CH_2_CH_3_, 2H), 3.40–3.53 (m, N^+^C*H*_2_CH_2_CH_2_S + 2 × HOCH_2_C*H*_2_, 6H), 3.75–3.87 (m, 2 × HOC*H*_2_CH_2_, 4H), 5.24 (t, *J* = 4.8 Hz, *H*OCH_2_CH_2_, 1H); ^13^C NMR (100 MHz, DMSO-*d*_6_) δ 13.45, 17.16, 19.13, 23.09, 51.11, 54.56, 57.39, 59.29, 60.19, 120.06 (q, *J*_CF_ = 322 Hz); ^19^F NMR (376 MHz, DMSO-*d*_6_) δ −76.56 (CF_3_, 3F); ESI-HRMS *m*/*z* [M + H]^+^ calculated for C_12_H_26_F_3_N_2_O_6_S_2_ 415.1179, found 415.1183 ([M + H]^+^), 437.0991 ([M + Na]^+^).

**ZIL 4d-ene** pale yellow liquid (25% yield); ^1^H NMR (400 MHz, DMSO-*d*_6_) δ 2.02–2.17 (m, N^+^CH_2_C*H*_2_CH_2_S, 2H), 2.43–2.55 (m, N^+^CH_2_C*H*_2_CH = CH_2_), 3.06 (t, *J* = 6.8 Hz, CH_2_C*H*_2_S, 2H), 3.39–3.60 (m, N^+^C*H*_2_CH_2_CH = CH_2_ + N^+^C*H*_2_CH_2_CH_2_S + 2 × HOCH_2_C*H*_2_, 8H), 3.74–3.93 (m, 2 × HOC*H*_2_CH_2_, 4H), 5.08–5.27 (m, N^+^CH_2_CH_2_CH = C*H*_2_, 2H), 5.32 (t, *J* = 4.8 Hz, *H*OCH_2_CH_2_, 1H), 5.65-5.78 (m, N^+^CH_2_CH_2_C*H*=CH_2_, 1H); ^13^C NMR (100 MHz, DMSO-*d*_6_) δ 17.18, 25.93, 51.07, 54.59, 60.29, 62.61, 118.52, 120.06 (q, *J*_CF_ = 322 Hz), 132.73; ^19^F NMR (376 MHz, DMSO-*d*_6_) δ −76.53 (CF_3_, 3F); ESI-HRMS *m*/*z* [M + H]^+^ calculated for C_12_H_24_F_3_N_2_O_6_S_2_ 413.1022, found 413.1025 ([M + H]^+^), 435.0845 ([M + Na]^+^).

**ZIL 4d-yne** pale yellow liquid (22% yield); ^1^H NMR (400 MHz, DMSO-*d*_6_) δ 1.93 (s, N^+^CH_2_C ≡ CC*H*_3_, 3H), 2.02–2.14 (m, N^+^CH_2_C*H*_2_CH_2_S, 2H), 3.07 (t, *J* = 6.8 Hz, CH_2_C*H*_2_S, 2H), 3.44–3.61 (m, N^+^C*H*_2_CH_2_CH_2_S + 2 × HOCH_2_C*H*_2_, 6H), 3.77–3.88 (m, 2 × HOC*H*_2_CH_2_, 4H), 4.37–4.44 (m, N^+^C*H*_2_C ≡ CCH_3_, 2H), 5.33 (t, *J* = 4.8 Hz, *H*OCH_2_CH_2_, 1H); ^13^C NMR (100 MHz, DMSO-*d*_6_) δ 3.40, 17.40, 51.22, 51.29, 54.61, 58.10, 60.56, 67.40, 88.79, 120.06 (q, *J*_CF_ = 322 Hz); ^19^F NMR (376 MHz, DMSO-*d*_6_) δ −76.52 (CF_3_, 3F); ESI-HRMS *m*/*z* [M + H]^+^ calculated for C_12_H_22_F_3_N_2_O_6_S_2_ 411.0866, found 411.0871 ([M + H]^+^), 433.0690 ([M + Na]^+^).

**ZIL 4e** pale yellow liquid (36% yield); ^1^H NMR (400 MHz, DMSO-*d*_6_) δ 0.88 (t, *J* = 7.2 Hz, N^+^CH_2_CH_2_(CH_2_)_2_C*H*_3_, 3H), 1.16–1.41 (m, N^+^CH_2_CH_2_(C*H*_2_)_2_CH_3_, 2H), 1.59–1.75 (m, N^+^CH_2_C*H*_2_(CH_2_)_2_CH_3_, 4H), 2.01–2.16 (m, N^+^CH_2_C*H*_2_CH_2_S, 2H), 3.06 (t, *J* = 6.8 Hz, CH_2_C*H*_2_S, 2H), 3.29–3.38 (m, N^+^C*H*_2_CH_2_(CH_2_)_2_CH_3_, 2H), 3.39–3.55 (m, N^+^C*H*_2_CH_2_CH_2_S + 2 × HOCH_2_C*H*_2_, 6H), 3.72–3.90 (m, 2 × HOC*H*_2_CH_2_, 4H), 5.25 (t, *J* = 4.8 Hz, *H*OCH_2_CH_2_, 1H); ^13^C NMR (100 MHz, DMSO-*d*_6_) δ 13.75, 17.13, 20.80, 21.61, 27.85, 51.08, 54.52, 57.33, 59.45, 60.13, 120.04 (q, *J*_CF_ = 322 Hz); ^19^F NMR (376 MHz, DMSO-*d*_6_) δ −76.57 (CF_3_, 3F); ESI-HRMS *m*/*z* [M + H]^+^ calculated for C_13_H_28_F_3_N_2_O_6_S_2_ 429.1335, found 429.1337 ([M + H]^+^), 451.1157 ([M + Na]^+^).

**ZIL 4f** pale yellow liquid (38% yield); ^1^H NMR (400 MHz, DMSO-*d*_6_) δ 0.87 (t, *J* = 6.8 Hz, N^+^CH_2_CH_2_(CH_2_)_3_C*H*_3_, 3H), 1.19–1.36 (m, N^+^CH_2_CH_2_(C*H*_2_)_3_CH_3_, 6H), 1.60–1.72 (m, N^+^CH_2_C*H*_2_(CH_2_)_3_CH_3_, 2H), 2.02–2.14 (m, N^+^CH_2_C*H*_2_CH_2_S, 2H), 3.06 (t, *J* = 7.2 Hz, CH_2_C*H*_2_S, 2H), 3.28–3.40 (m, N^+^C*H*_2_CH_2_(CH_2_)_3_CH_3_, 2H), 3.40–3.55 (m, N^+^C*H*_2_CH_2_CH_2_S + 2 × HOCH_2_C*H*_2_, 6H), 3.73–3.86 (m, 2 × HOC*H*_2_CH_2_, 4H), 5.24 (t, *J* = 4.8 Hz, *H*OCH_2_CH_2_, 1H); ^13^C NMR (100 MHz, DMSO-*d*_6_) δ 13.78, 17.12, 21.03, 21.86, 25.34, 30.58, 51.07, 54.51, 57.34, 59.47, 60.13, 120.03 (q, *J*_CF_ = 322 Hz); ^19^F NMR (376 MHz, DMSO-*d*_6_) δ −76.57 (CF_3_, 3F); ESI-HRMS *m*/*z* [M + H]^+^ calculated for C_14_H_30_F_3_N_2_O_6_S_2_ 443.1492, found 443.1497 ([M + H]^+^), 465.1309 ([M + Na]^+^).

## 4. Conclusions

The ionic liquid [choline][NTf_2_] (**IL 1**) is established as an important hydrogen bonding between the hydroxyl group of the choline cation and one sulfonyl oxygen on NTf_2_ anion,^6^ resulting in its immiscibility with water at ambient temperature but becoming water soluble above its *T*_c_ (72 °C), and accordingly displayed an UCST phase transition in water. As shown in Figure 1, our molecular engineering of **IL 2** as well as the ZILs (**ZIL 3a**–**f** and **ZIL 4a**–**f**) is composed of such potential hydrogen bonding and they should exhibit temperature-dependent UCST behavior, if TILs are discovered experimentally. Additionally, because phase behavior of a TIL is a delicate and sensitive balance between total hydrophobicity and hydrophilicity of the ionic liquid investigated, a TIL if identified should reside on the rim between being totally hydrophilic and totally hydrophobic in ZILs. Ultimately, the successful combinatory synthesis and screening from libraries of small-molecule ZILs should be practical to establish the structure-property relationship (SAT).

We reported in this work on the synthesis of a new **IL 2** and a small library of 16 ZILs, experimentally observed **IL 2** and 4 ZILs (**ZIL 3d**, **ZIL 3d-ene**, **ZIL 3d-yne**, and **ZIL 4d**) exhibiting UCST property in water, and highlighted preliminarily the important role of ZIL played in biomolecular recognition study. Also in this research, we unambiguously demonstrated the structural tunability of ZILs and their close relationship with thermoresponsiveness (SAT); that is, based on the structure-and-miscibility gap study, the successful engineering of **ZIL 3d** (*T_c_* = 84 °C), **ZIL 3d-ene** (*T_c_* = 53 °C) and **ZIL 3d-yne** (*T_c_* = 33 °C) perfectly exemplified the true value of the fine tunability of ZIL structures. This work is not only the combinatorial discovery of non-aromatic and acyclic NTf-based ZILs that exhibit UCST phase transitions with water, but also a rational approach of structural fine-tuning in polarity to ultimately afford ZILs with biocompatible *T_c_* values. The results presented in this work hold a compelling possibility for potential use of ZILs as small-molecule thermoresponsive materials for biomolecular recognition study.

## Data Availability

Not applicable.

## Supplementary Materials

The following are available online, Figure S1: *T_c_* values of **IL 1** (72 °C), **IL 2** (13 °C), **ZIL 3d** (84 °C), and **ZIL 4d** (10 °C), Figure S2: Temperature dependence of phase behavior of mixtures (1:1, *w/w*) of water with **ZIL 3c**, **ZIL 3e**, and a binary mixture (1:1, *w/w*) of **ZIL 3c** and **ZIL 3e** exhibiting *T_c_* = 81 °C (labeled in red), Detailed ^1^H and ^13^C NMR, and high-resolution mass spectrometry (HRMS) spectra of all 14 ionic liquids (**IL 1**, **IL 2**, **ZIL 3a–f**, and **ZIL 4a–f**).

## Sample Availability

Samples of the compounds studied are unavailable from the authors for distribution.
