# Supplementary material for: Structural Engineering and Optimization of Zwitterionic Salts for Expeditious Discovery of Thermoresponsive Materials
Source: Molecules. 2021 Dec 31;27(1):257. doi: 10.3390/molecules27010257 (PMC8746428; doi:10.3390/molecules27010257)
Supplement: Supplementary file 1 [file molecules-27-00257-s001.zip › molecules-1510208-supplementary.pdf]

(Supporting Information)

Structural Engineering and Optimization of Zwitterionic Salts for Expeditious Discovery of Thermoresponsive Materials

Yen-Ho Chu*, Chien-Yuan Chen, and Jin-Syuan Chen

Department of Chemistry and Biochemistry, National Chung Cheng University

Chiayi 62102, Taiwan, Republic of China

* Corresponding author. Tel: 886 52729139; fax: 886 52721040; e-mail: cheyhc@ccu.edu.tw

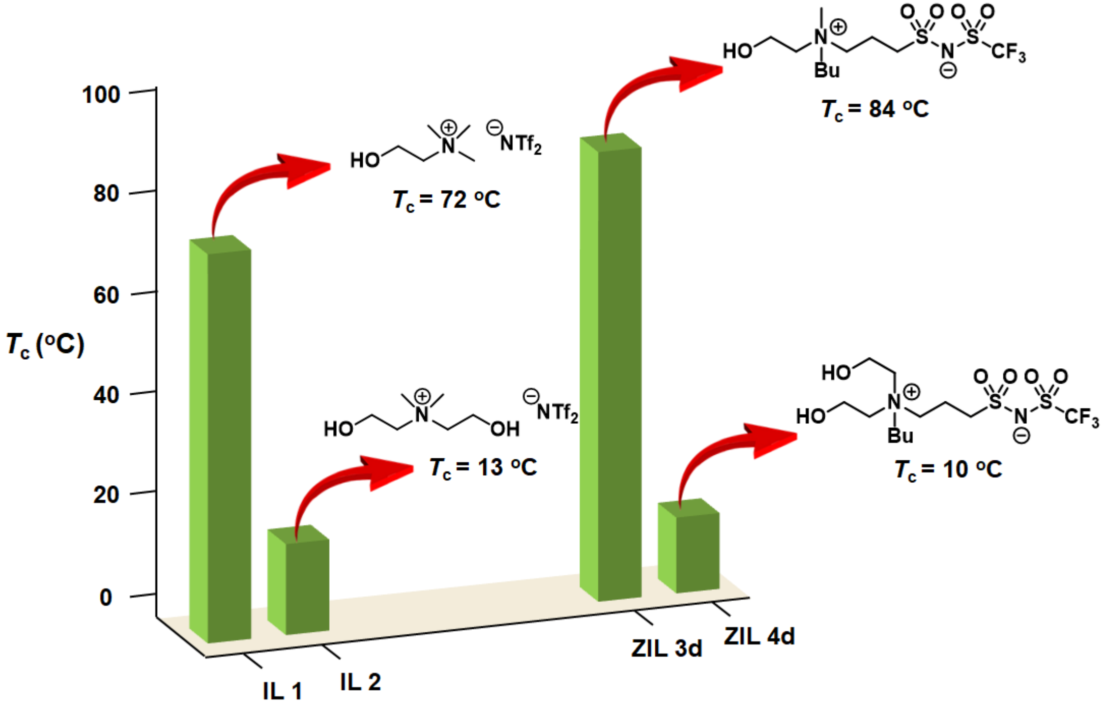

Figure S1 T_c values of **IL 1** (72 ºC), **IL 2** (13 ºC), **ZIL 3d** (84 ºC), and **ZIL 4d** (10 ºC).

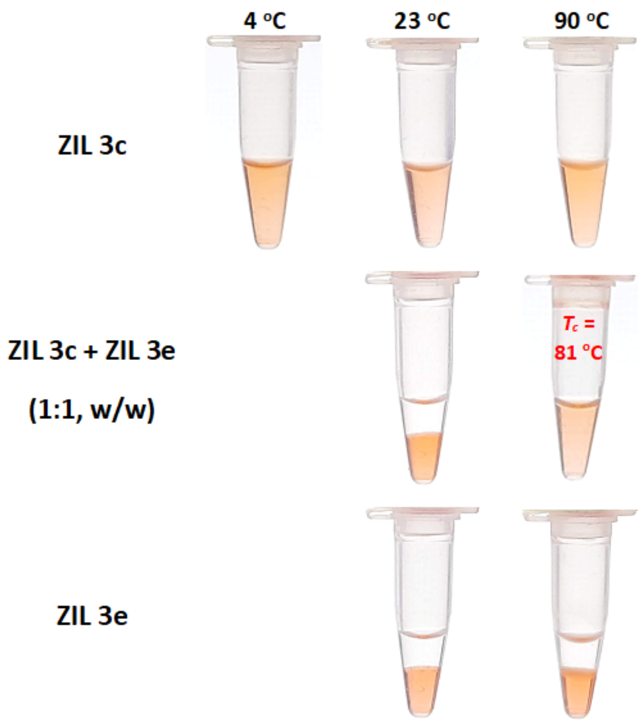

Figure S2 Temperature dependence of phase behavior of mixtures (1:1, w/w) of water with **ZIL 3c**, **ZIL 3e**, and a binary mixture (1:1, w/w) of **ZIL 3c** and **ZIL 3e** exhibiting T_c = 81 °C (labeled in red).

^{1}H NMR spectrum of **IL 2**

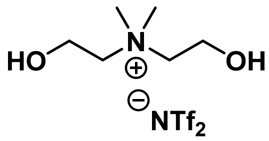

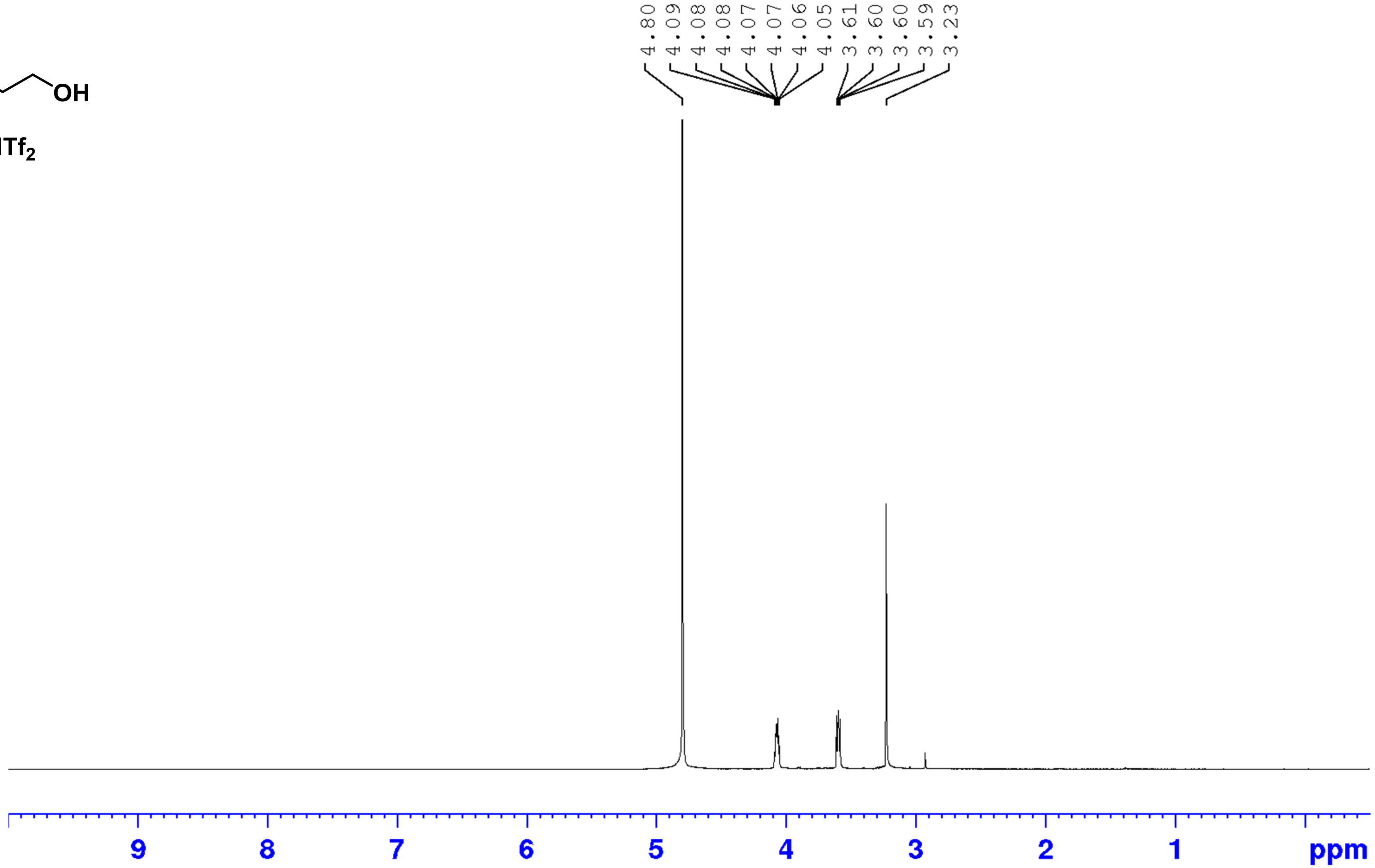

^{13}C NMR spectrum of **IL 2**

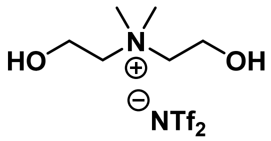

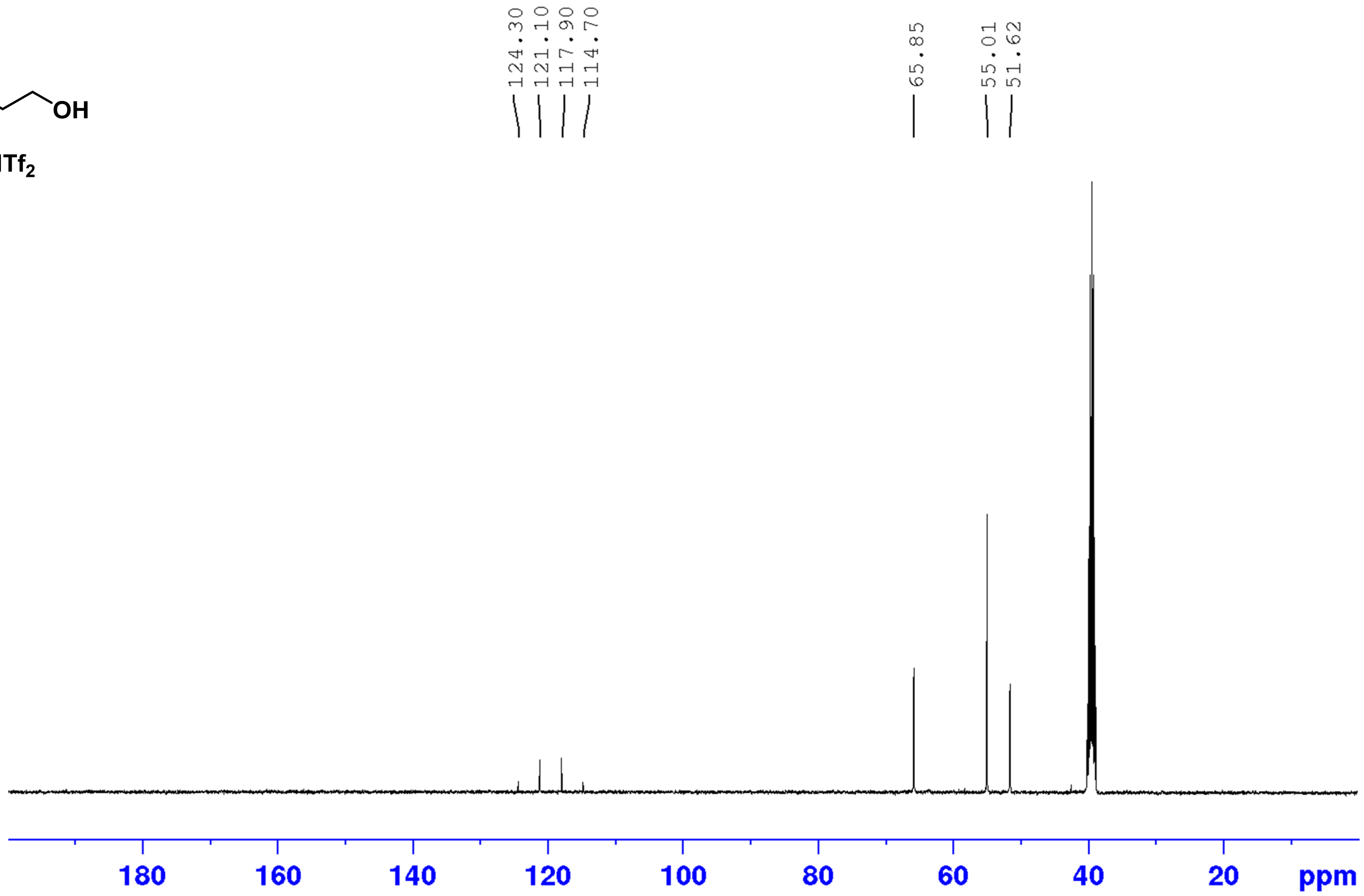

Mass spectrum of **IL 2**

Spectrum

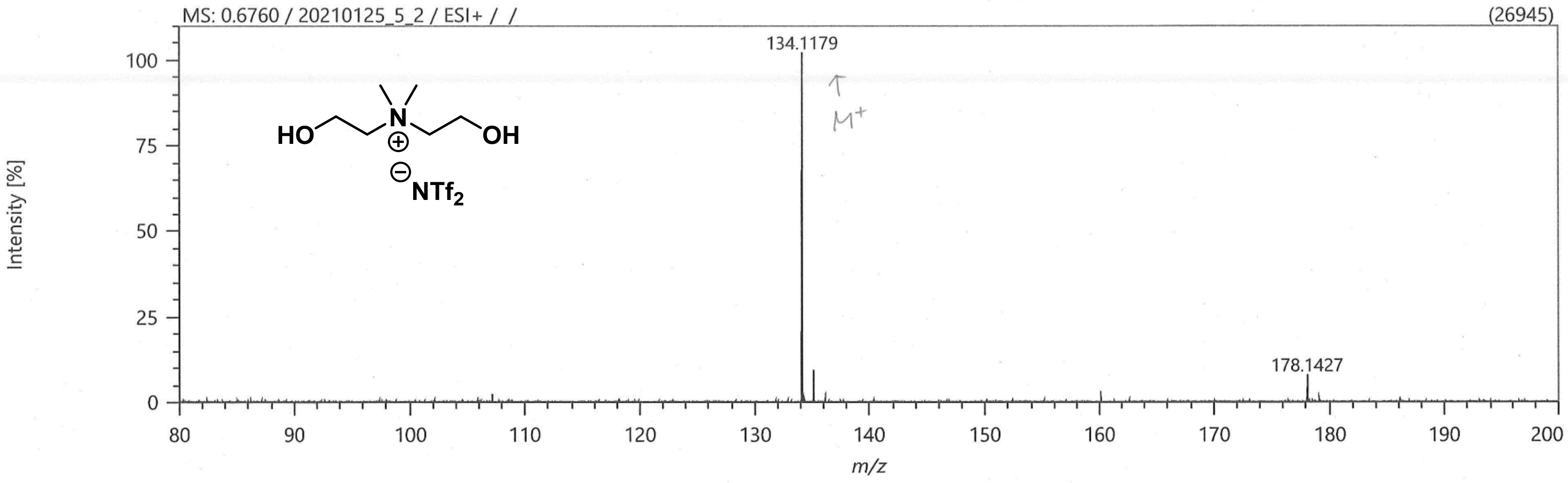

Elemental Composition

Parameters		Elements Set 1:				
Tolerance:	±10.00 ppm	Symbol	C	H	N	O
Electron:	Odd/Even	Min	0	0	1	2
Charge:	+1	Max	400	1000	1	2
DBE:	-99.0 - 999.0					

Results

Mass	Formula	Calculated Mass	Mass Difference [mDa]	Mass Difference [ppm]	DBE
134.11790	C6 H16 N O2	134.11756	0.34	2.54	-0.5

^{1}H NMR spectrum of 3-chloropropane-1-sulfonyl chloride (**2**)

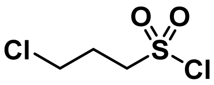

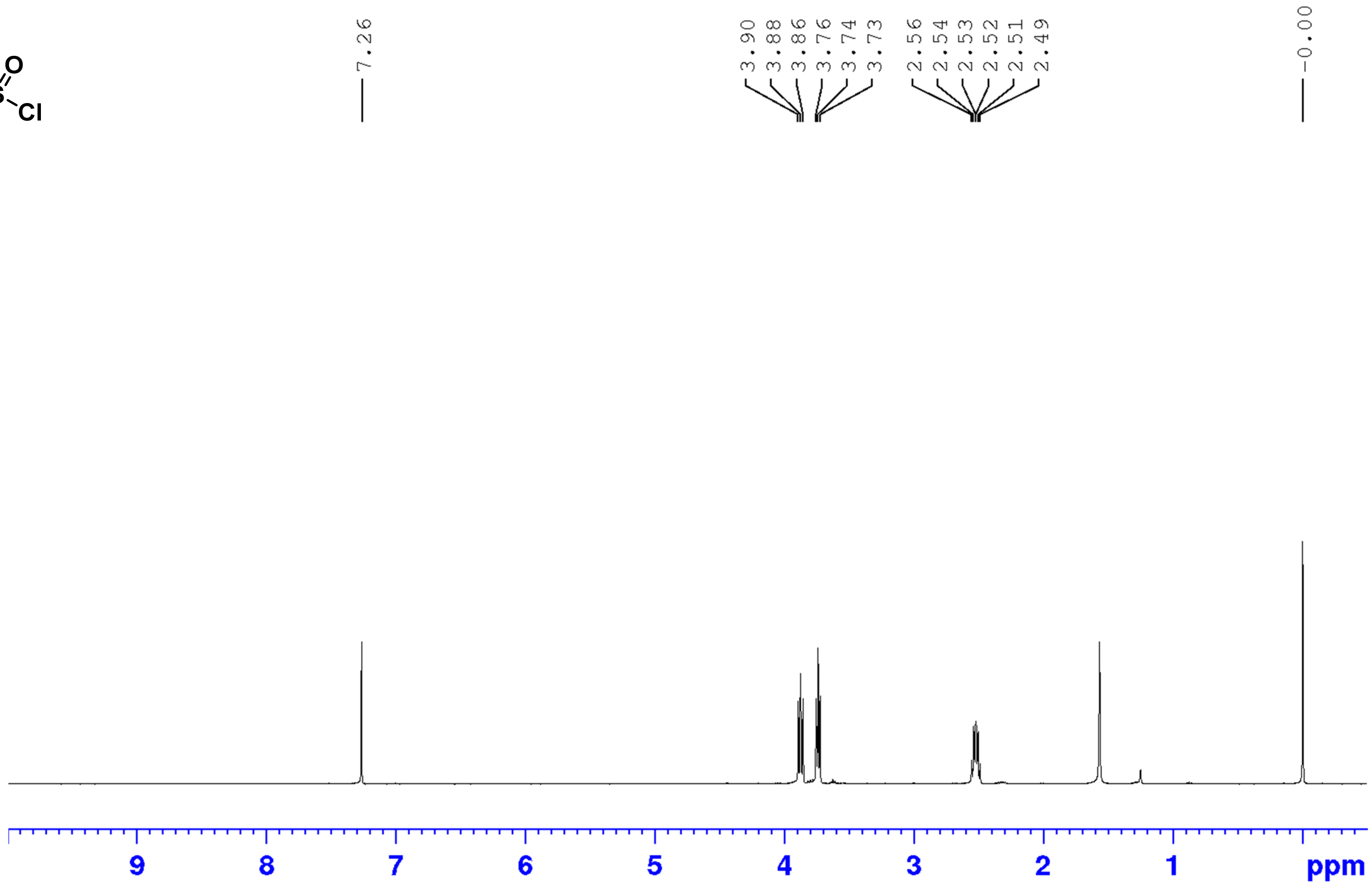

^{1}H NMR spectrum of potassium ((3-chloropropyl)sulfonyl)((trifluoromethyl)sulfonyl)amide (**3**)

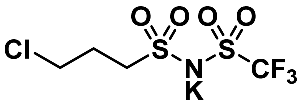

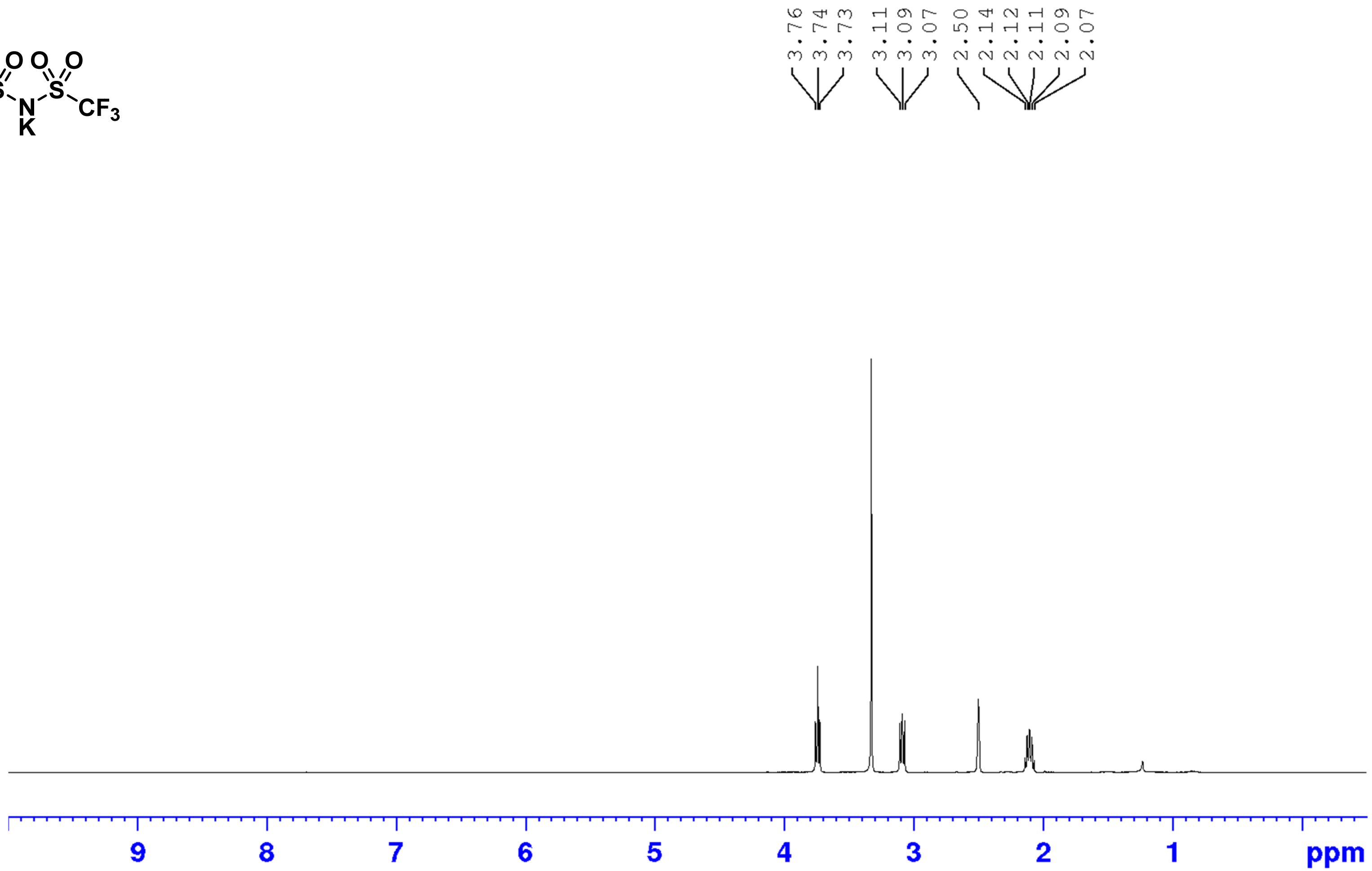

^{19}F NMR spectrum of potassium ((3-chloropropyl)sulfonyl)((trifluoromethyl)sulfonyl)amide (**3**)

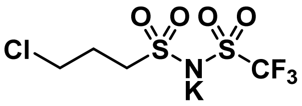

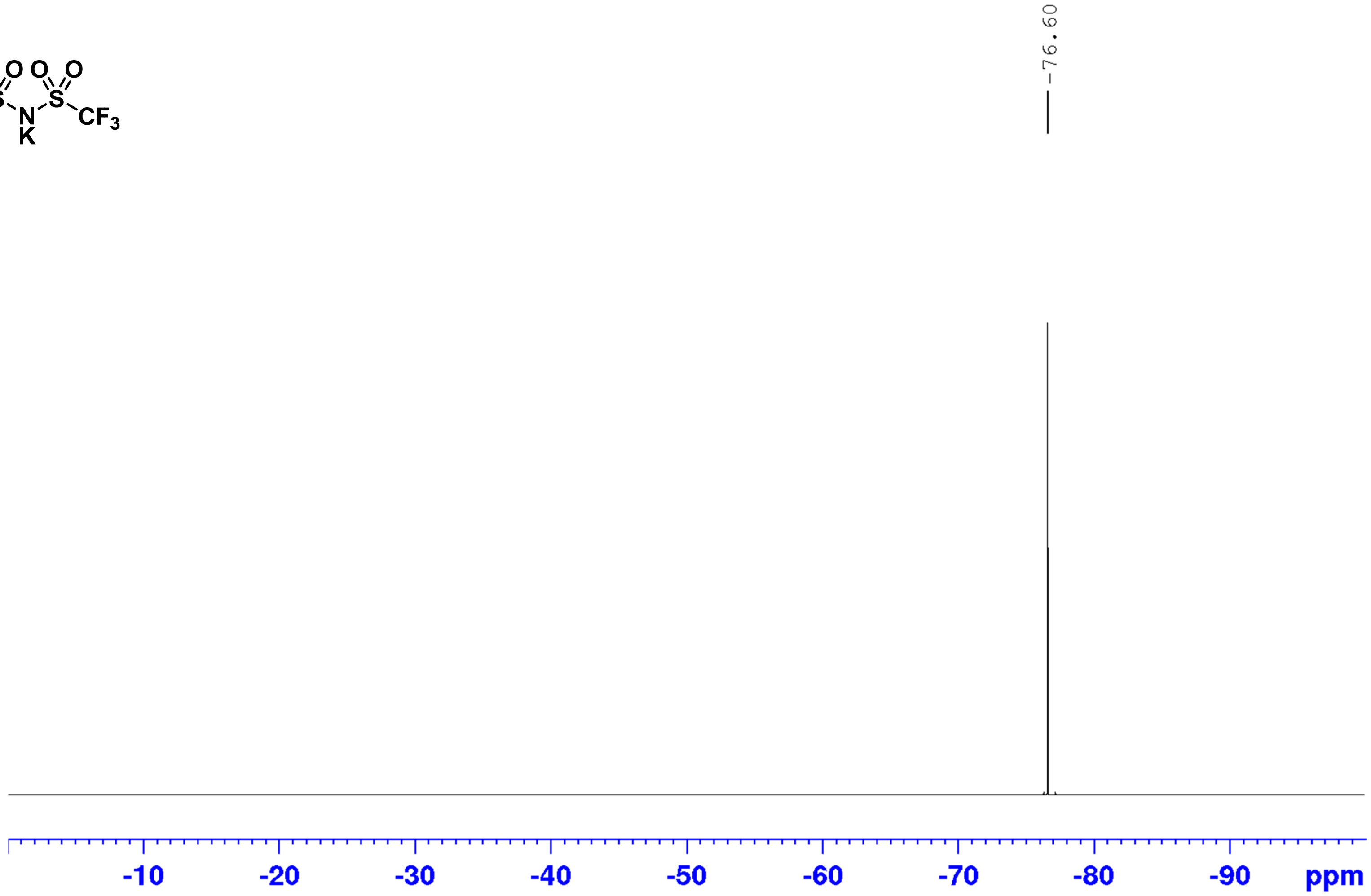

^{1}H NMR spectrum of **ZIL 3a**

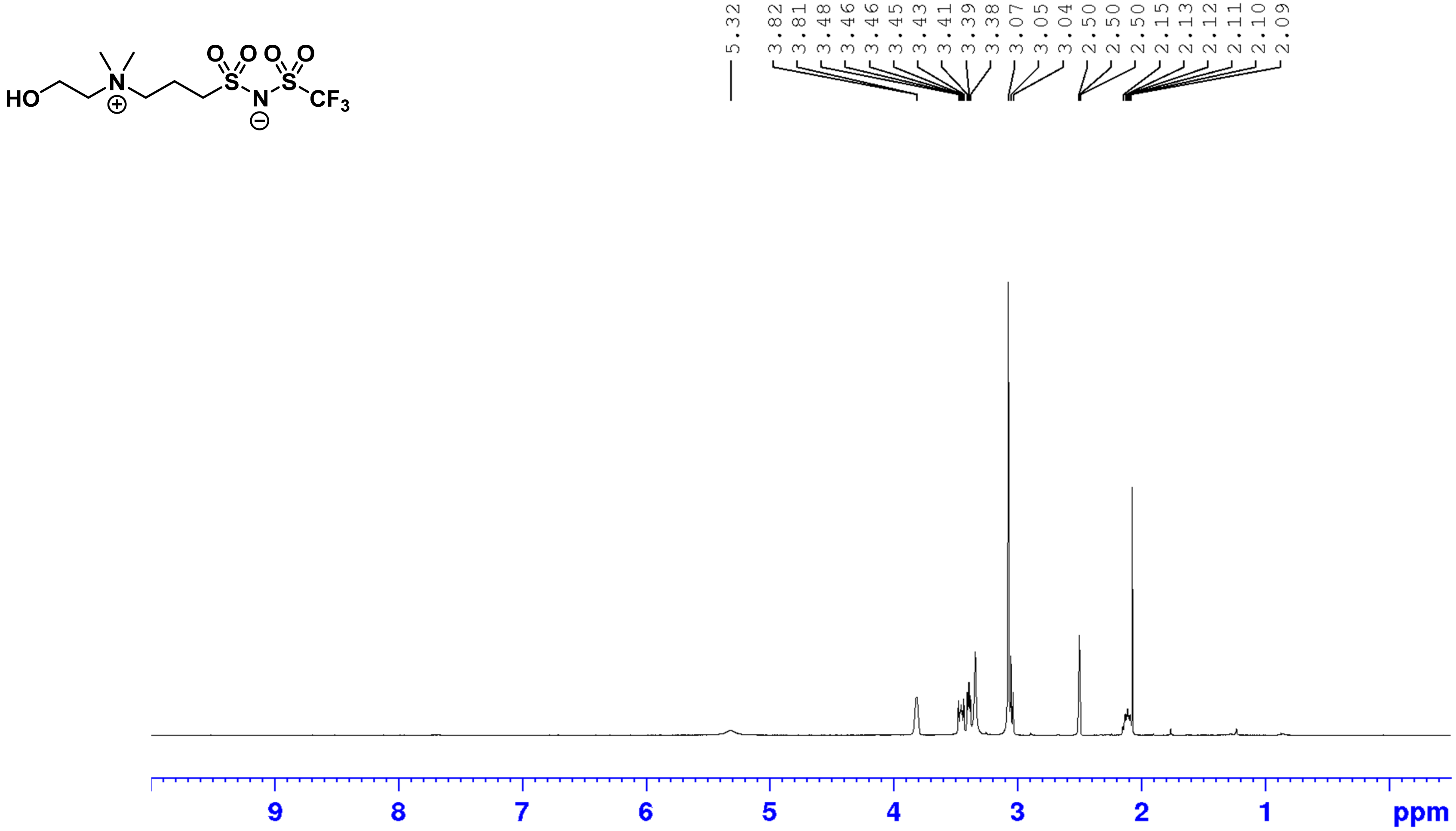

^{19}F NMR spectrum of **ZIL 3a**

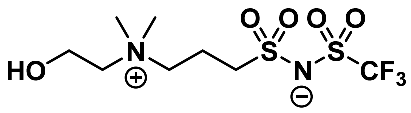

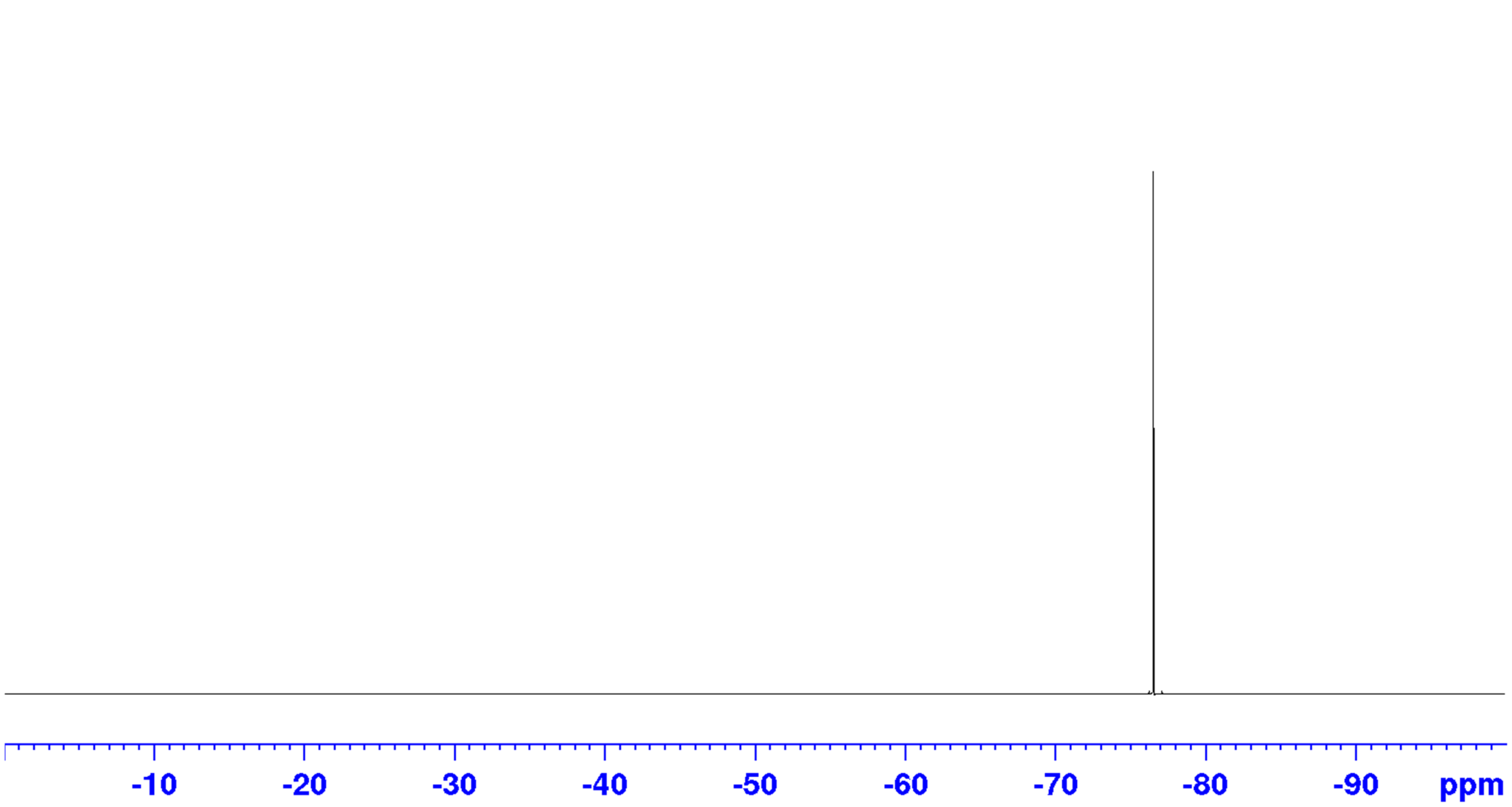

^{13}C NMR spectrum of **ZIL 3a**

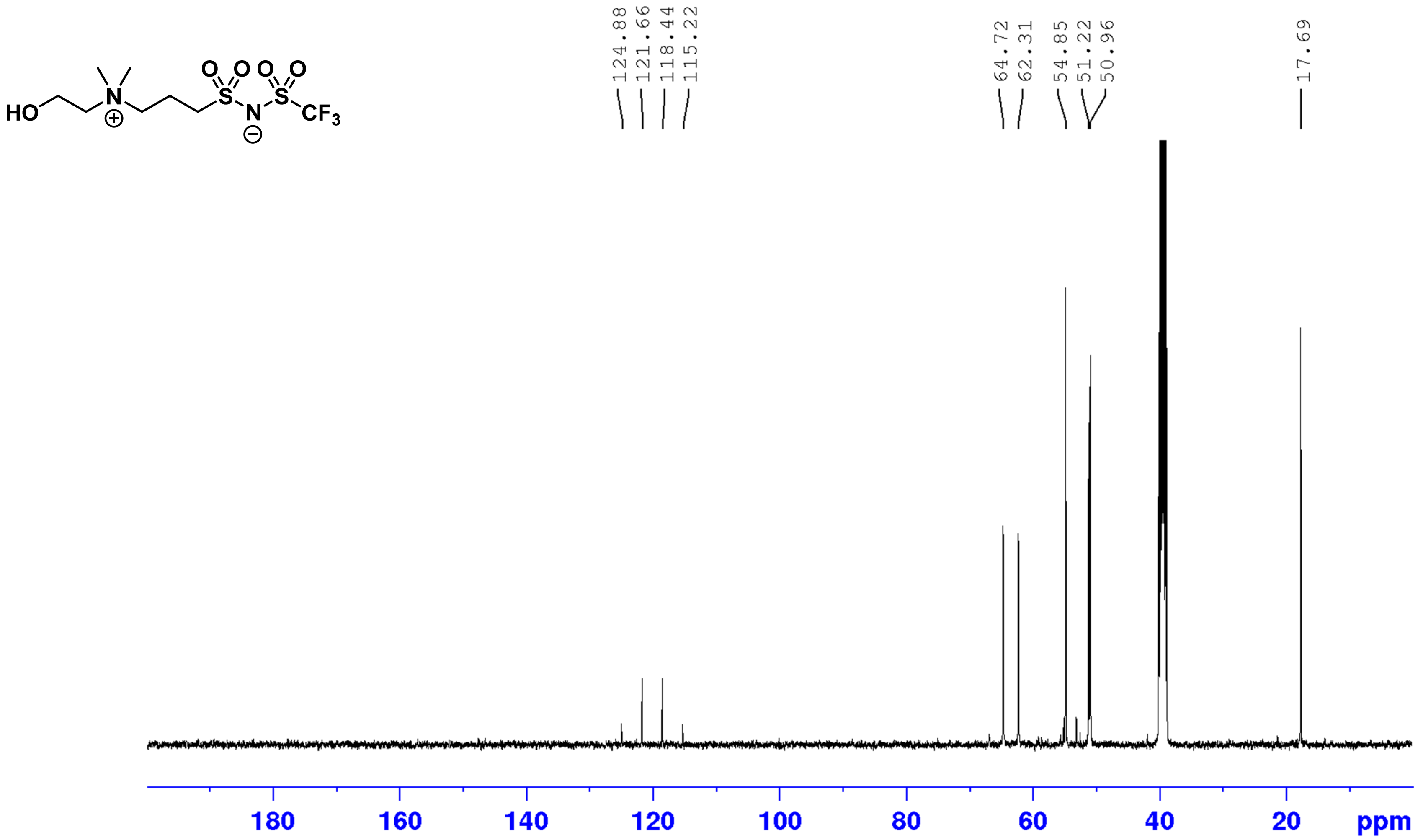

Mass spectrum of **ZIL 3a**

Spectrum

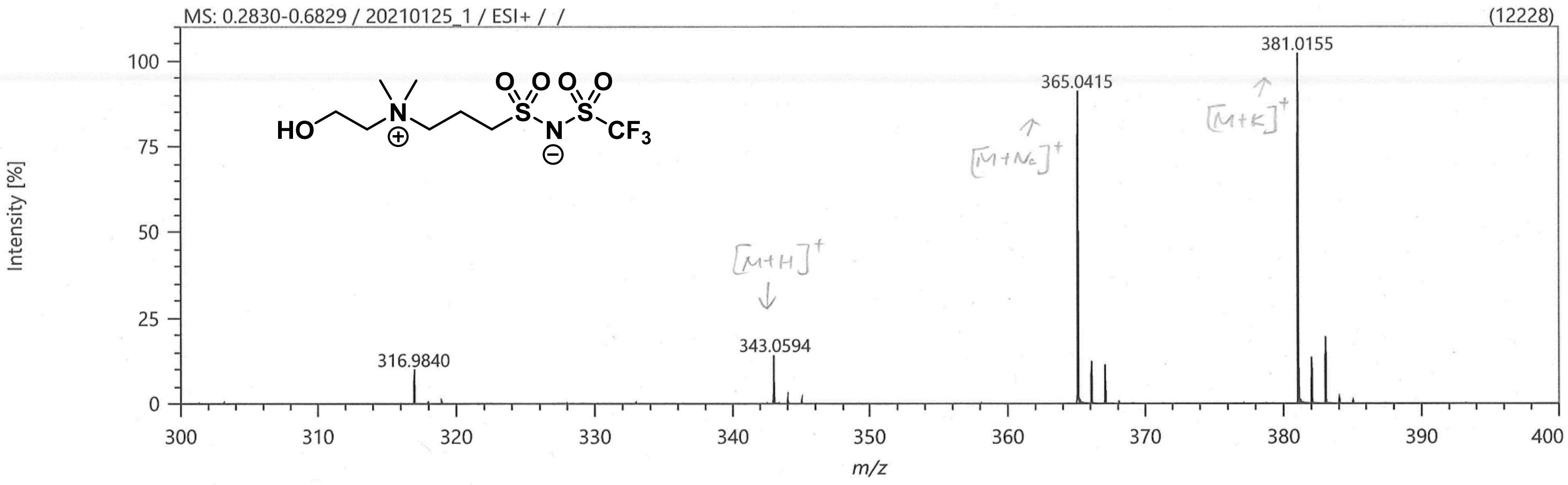

Elemental Composition

Parameters		Elements Set 1:								
Tolerance:	±3.00 ppm	Symbol	C	H	N	O	S	F	Na	K
Electron:	Odd/Even	Min	0	0	2	5	2	3	0	0
Charge:	+1	Max	400	1000	2	5	2	3	1	1
DBE:	-99.0 - 999.0									

Results

Mass	Formula	Calculated Mass	Mass Difference [mDa]	Mass Difference [ppm]	DBE
343.05943	C8 H18 N2 O5 F3 S2	343.06037	-0.94	-2.74	-0.5
365.04147	C8 H17 N2 O5 F3 Na S2	365.04232	-0.85	-2.32	-0.5
381.01550	C8 H17 N2 O5 F3 S2 K	381.01626	-0.75	-1.97	-0.5

^{1}H NMR spectrum of **ZIL 3b**

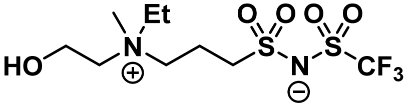

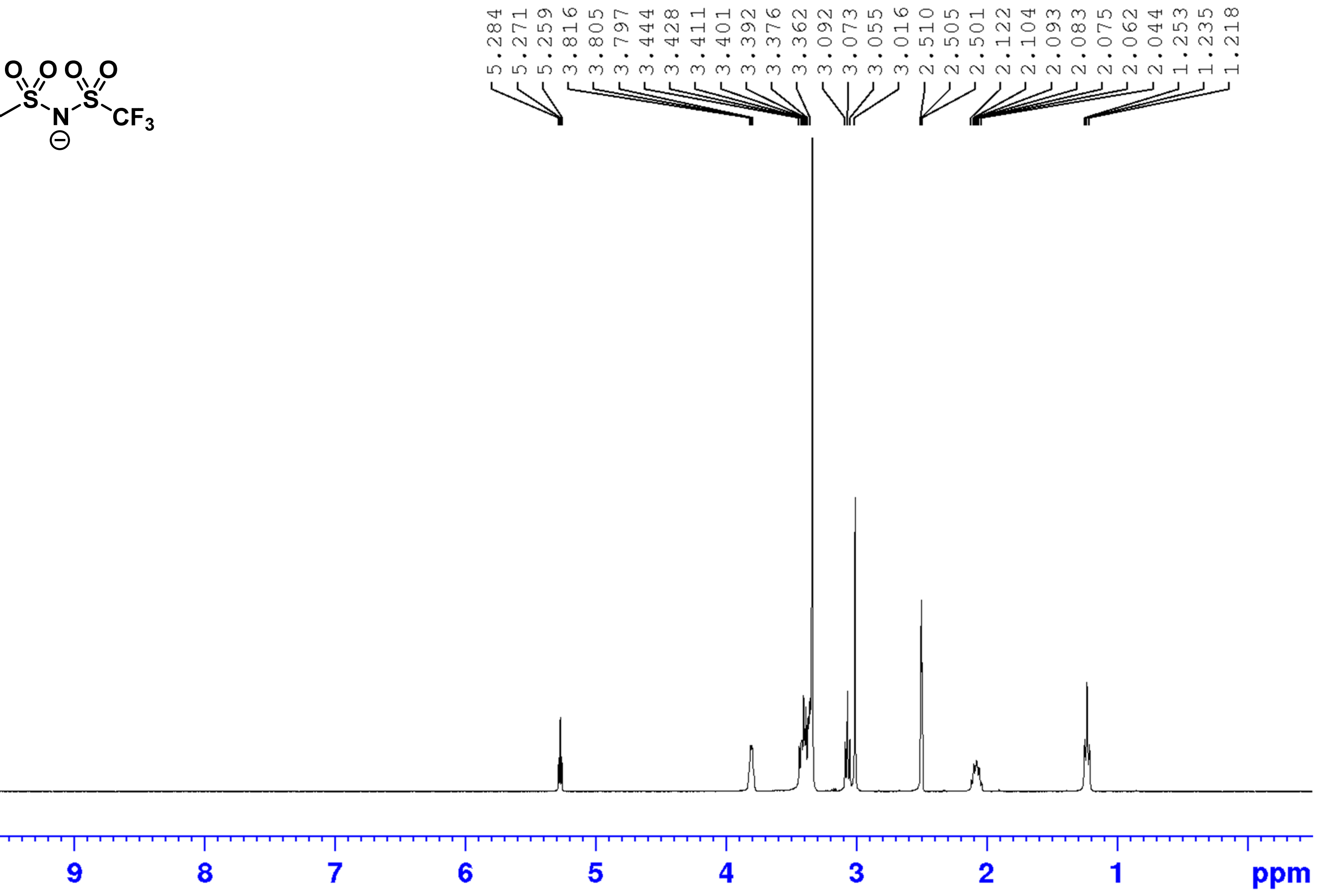

^{19}F NMR spectrum of **ZIL 3b**

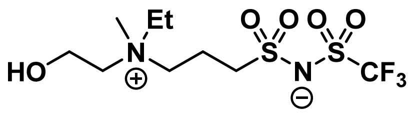

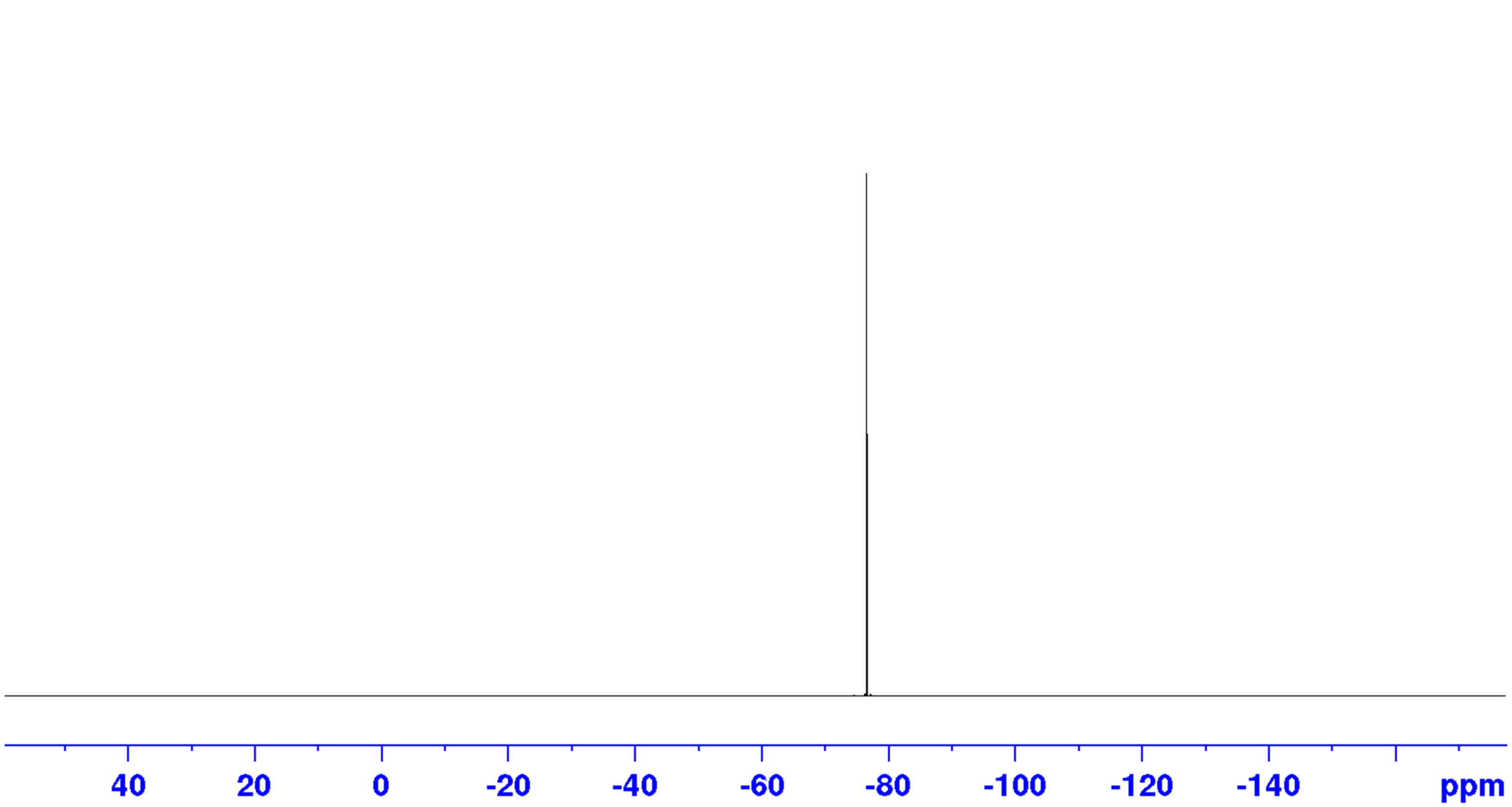

^{13}C NMR spectrum of **ZIL 3b**

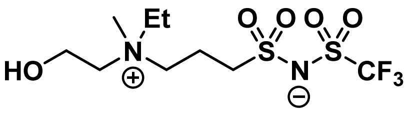

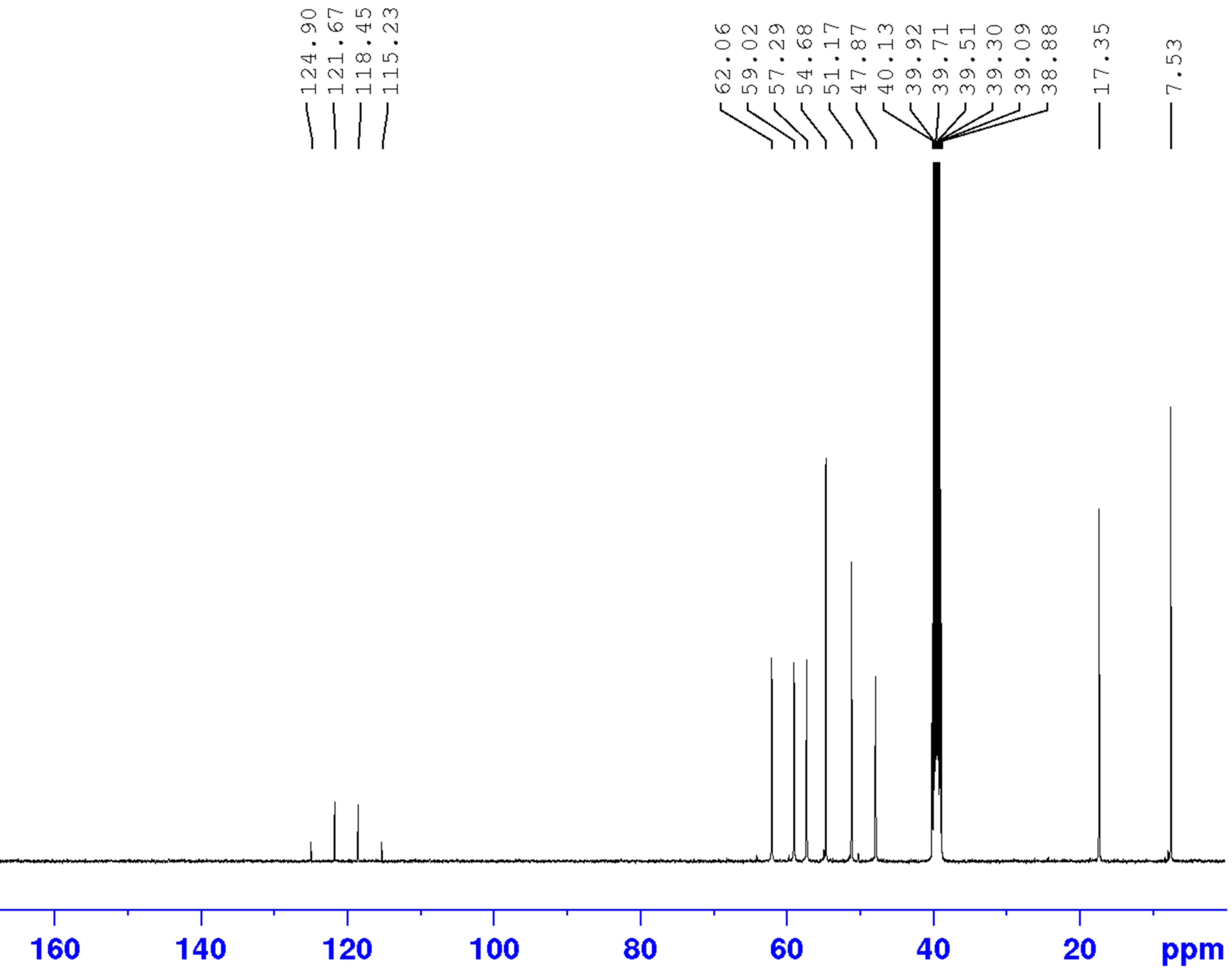

Mass spectrum of **ZIL 3b**

Spectrum

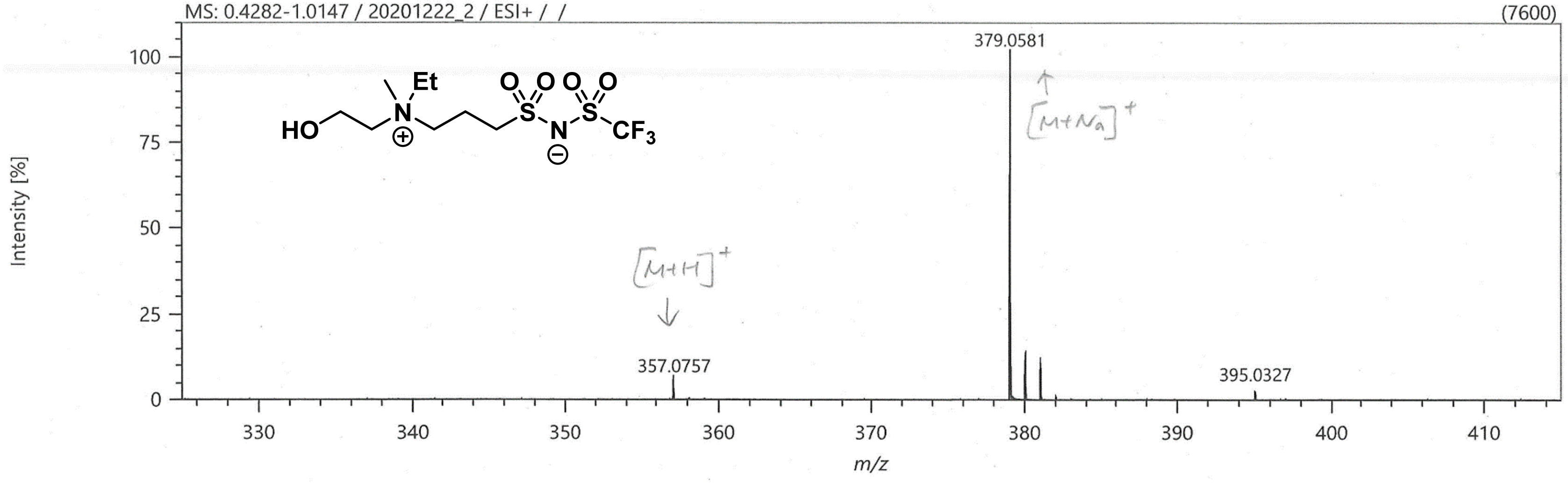

Elemental Composition

Parameters		Elements Set 1:							
Tolerance:	±2.00 ppm	Symbol	C	H	F	N	O	S	Na
Electron:	Odd/Even	Min	0	0	3	2	5	2	0
Charge:	+1	Max	400	1000	3	2	5	2	1
DBE:	-99.0 - 999.0								

Results

Mass	Formula	Calculated Mass	Mass Difference [mDa]	Mass Difference [ppm]	DBE
357.07565	C9 H20 N2 O5 F3 S2	357.07602	-0.37	-1.04	-0.5
379.05809	C9 H19 N2 O5 F3 Na S2	379.05797	0.12	0.33	-0.5

^{1}H NMR spectrum of **ZIL 3c**

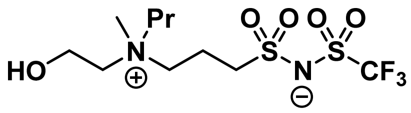

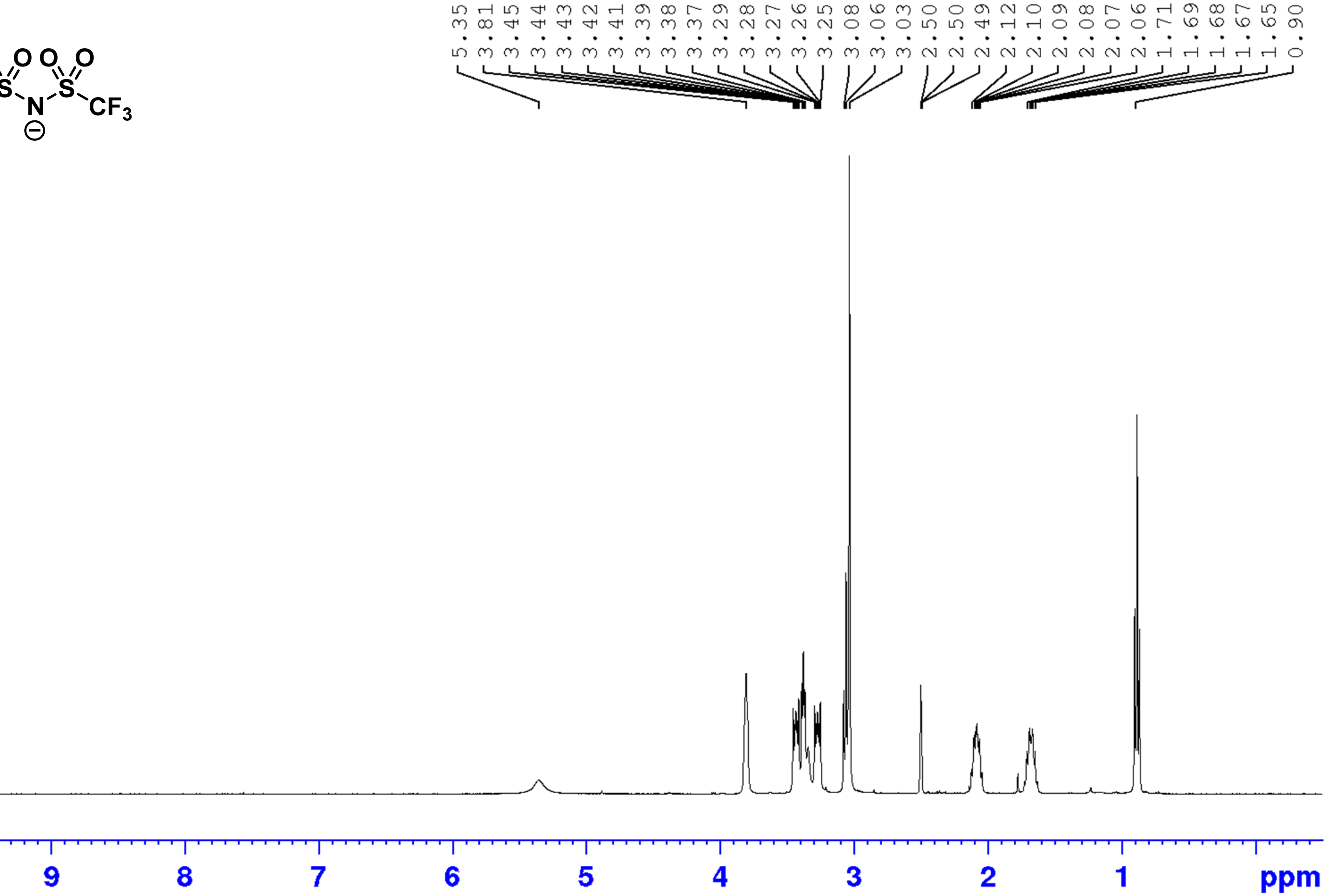

^{19}F NMR spectrum of **ZIL 3c**

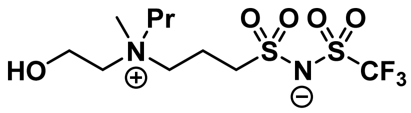

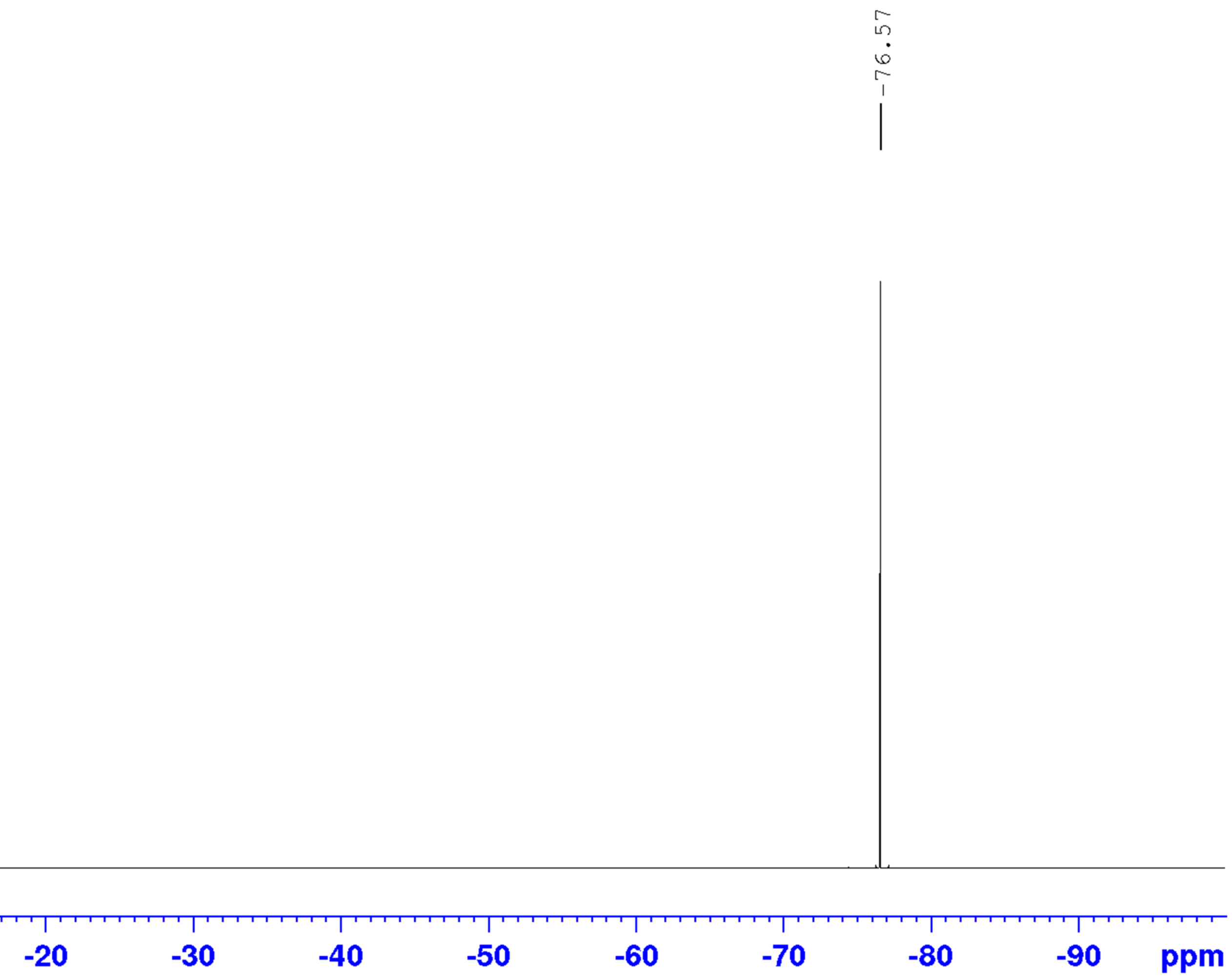

^{13}C NMR spectrum of **ZIL 3c**

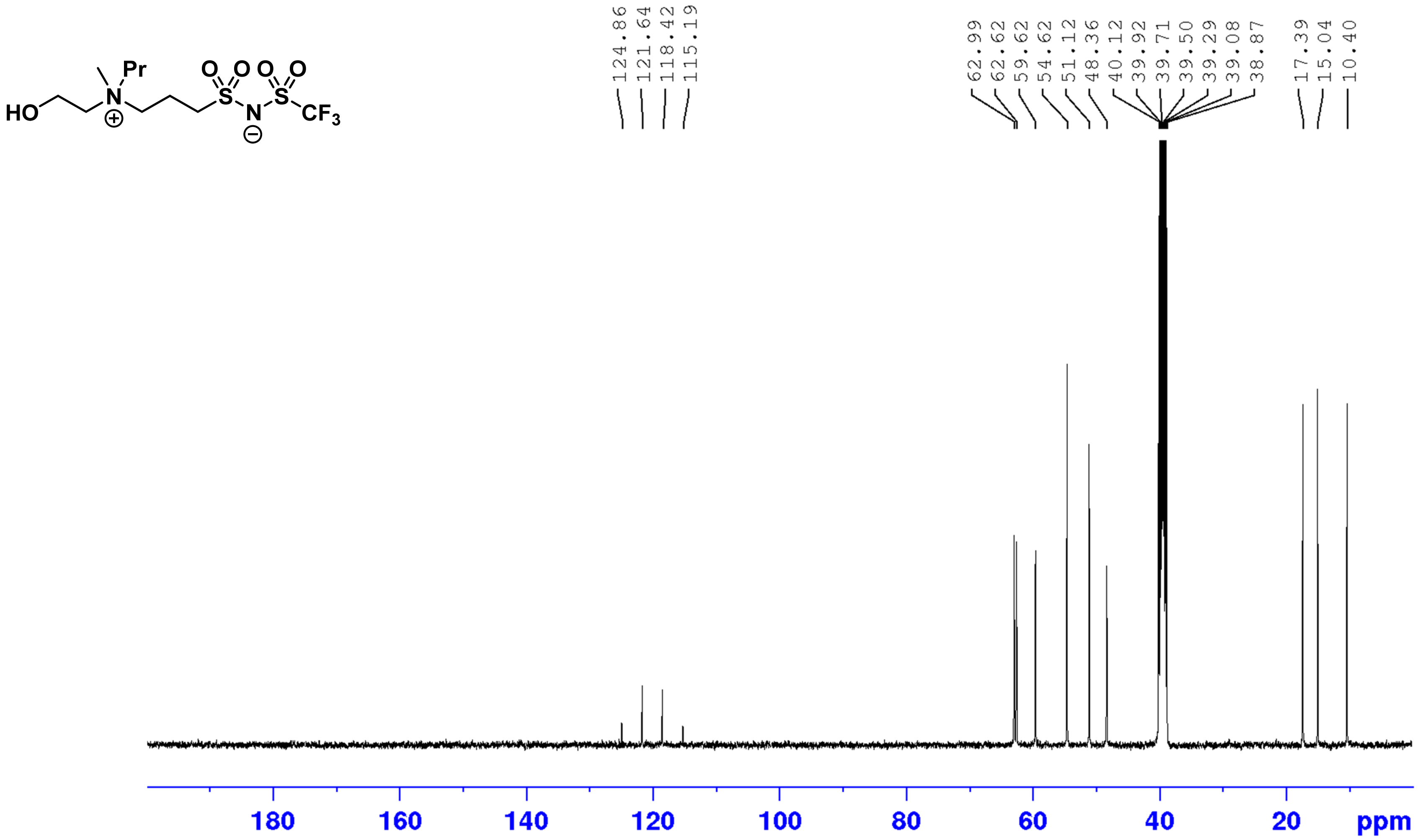

Mass spectrum of **ZIL 3c**

Spectrum

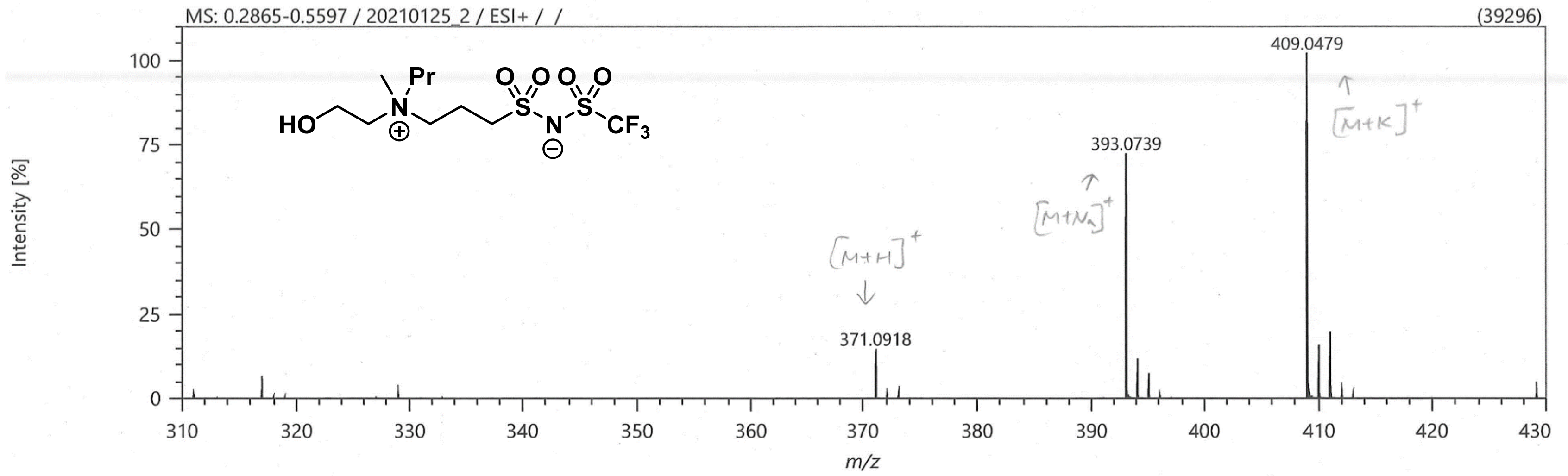

Elemental Composition

Parameters

Tolerance:	±3.00 ppm
Electron:	Odd/Even
Charge:	+1
DBE:	-99.0 - 999.0

Elements Set 1:

Symbol	C	H	N	O	S	F	Na	K
Min	0	0	2	5	2	3	0	0
Max	400	1000	2	5	2	3	1	1

Results

Mass	Formula	Calculated Mass	Mass Difference [mDa]	Mass Difference [ppm]	DBE
371.09184	C10 H22 N2 O5 F3 S2	371.09167	0.16	0.44	-0.5
393.07385	C10 H21 N2 O5 F3 Na S2	393.07362	0.23	0.59	-0.5
409.04788	C10 H21 N2 O5 F3 S2 K	409.04756	0.33	0.80	-0.5

^{1}H NMR spectrum of **ZIL 3d**

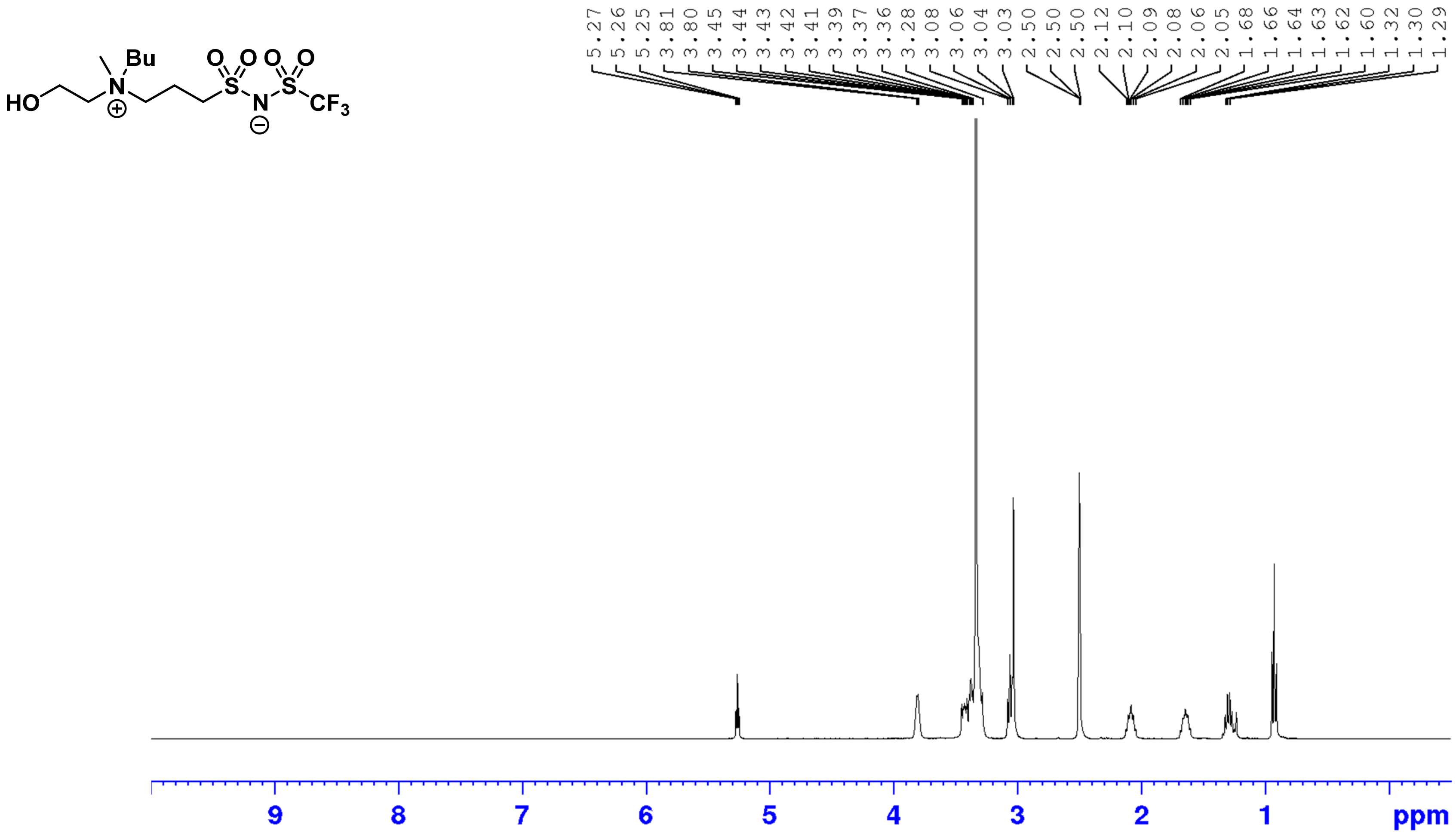

^{19}F NMR spectrum of **ZIL 3d**

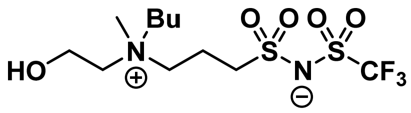

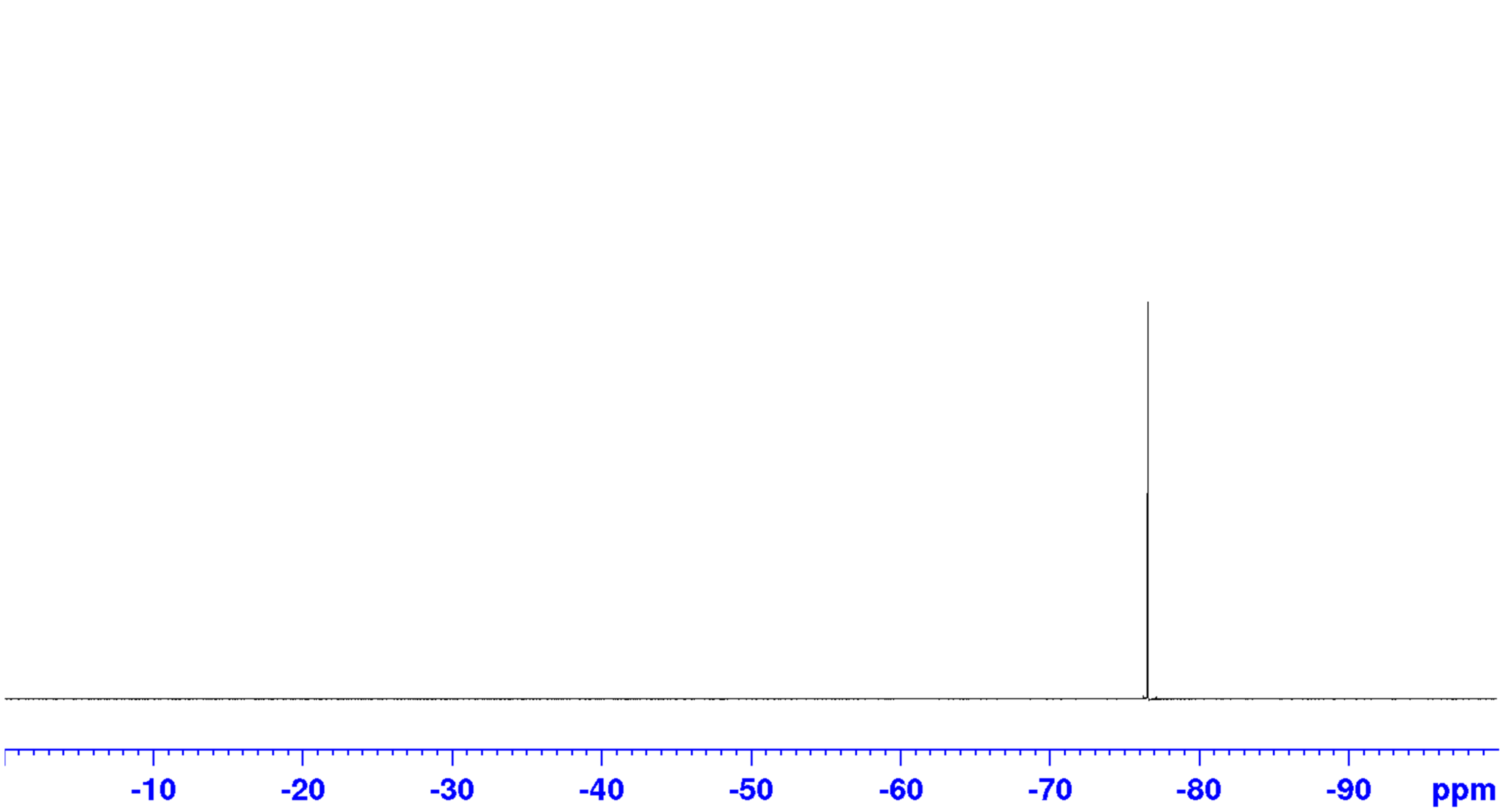

^{13}C NMR spectrum of **ZIL 3d**

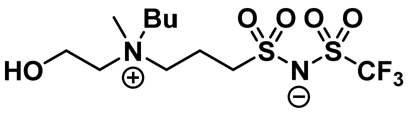

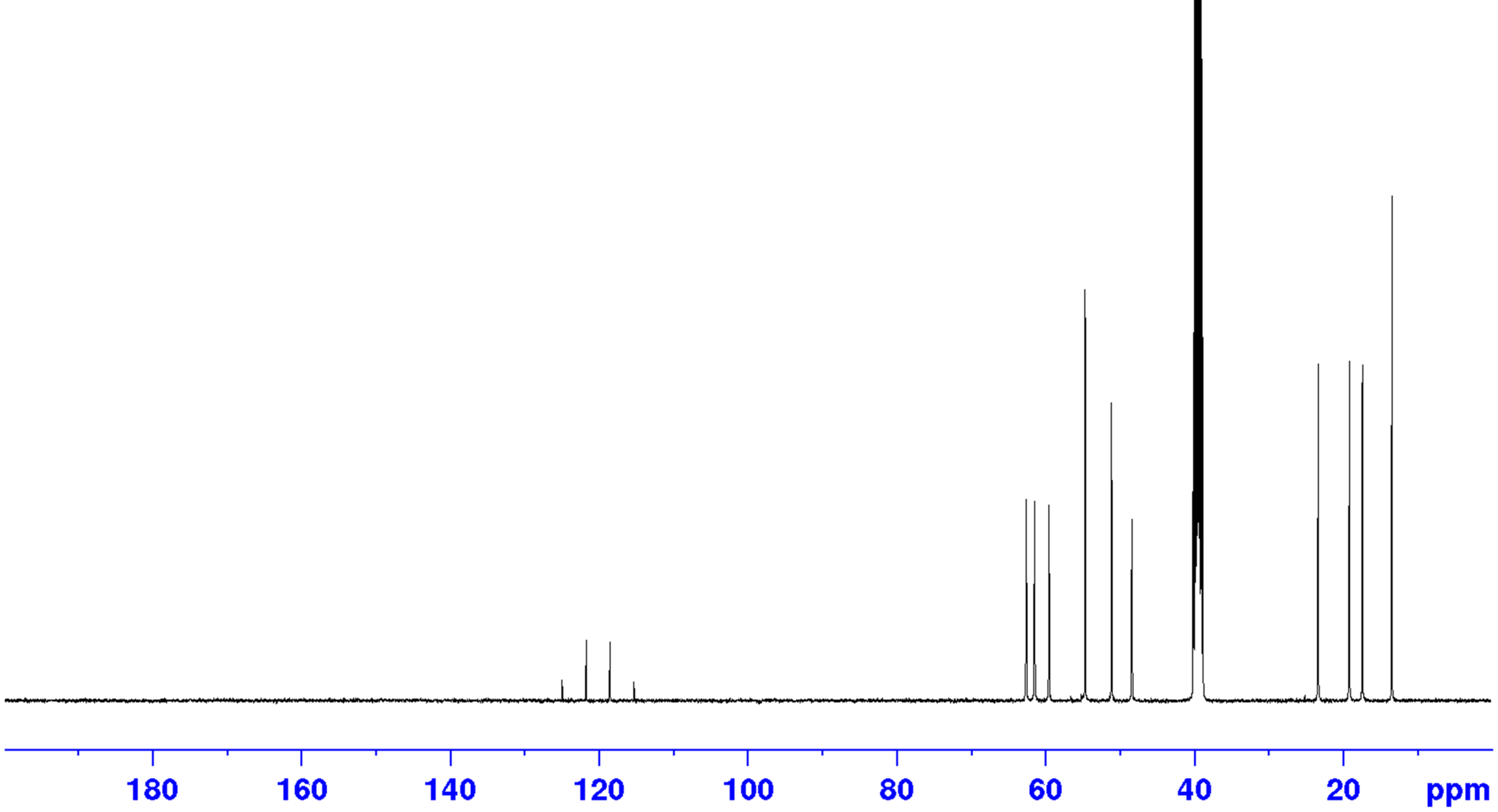

Mass spectrum of **ZIL 3d**

Spectrum

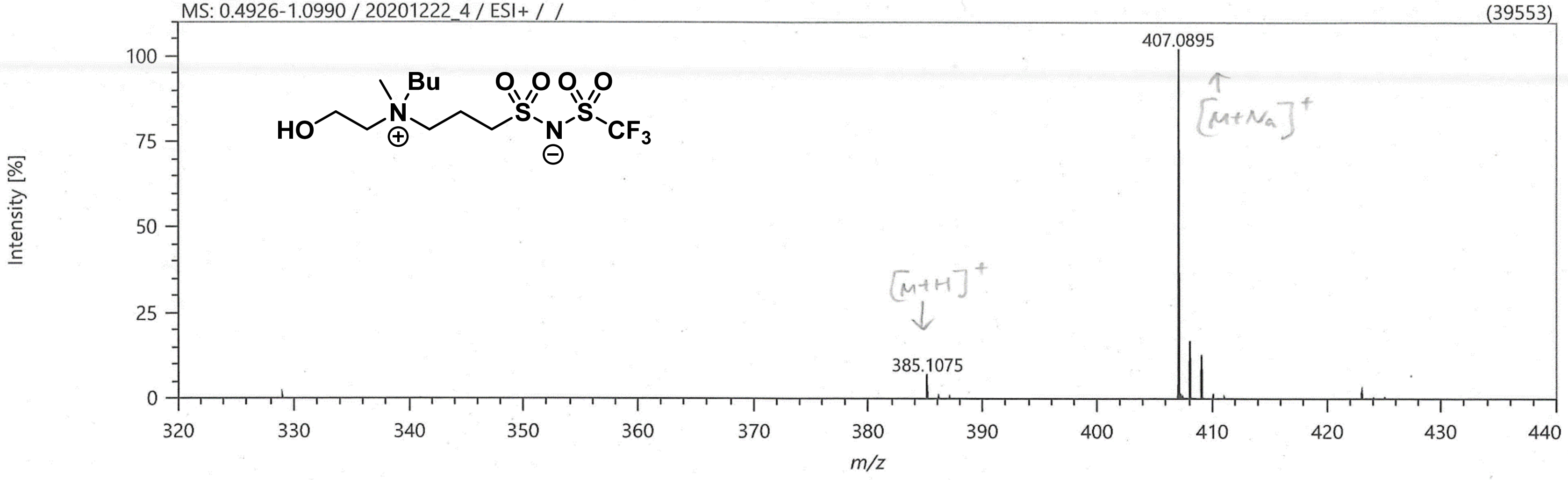

Elemental Composition

Parameters		Elements Set 1:							
		Symbol	C	H	F	N	O	S	Na
Tolerance:	±2.00 ppm								
Electron:	Odd/Even	Min	0	0	3	2	5	2	0
Charge:	+1	Max	400	1000	3	2	5	2	1
DBE:	-99.0 - 999.0								

Results

Mass	Formula	Calculated Mass	Mass Difference [mDa]	Mass Difference [ppm]	DBE
385.10747	C11 H24 N2 O5 F3 S2	385.10732	0.14	0.38	-0.5
407.08946	C11 H23 N2 O5 F3 Na S2	407.08927	0.19	0.46	-0.5

^{1}H NMR spectrum of **ZIL 3d-ene**

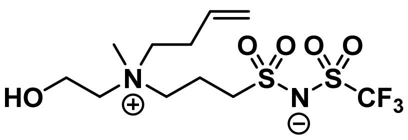

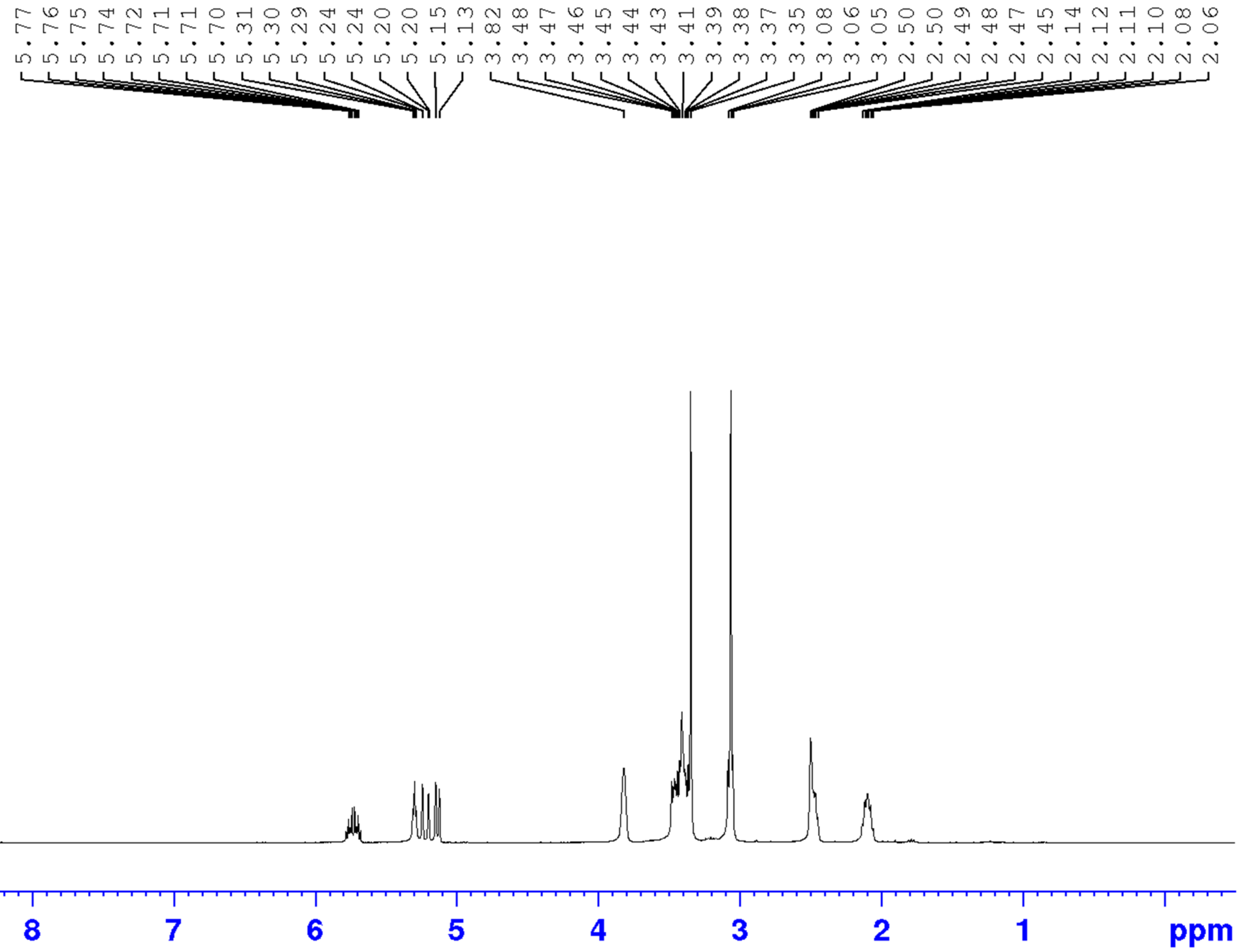

^{19}F NMR spectrum of **ZIL 3d-ene**

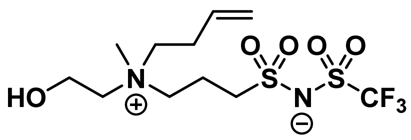

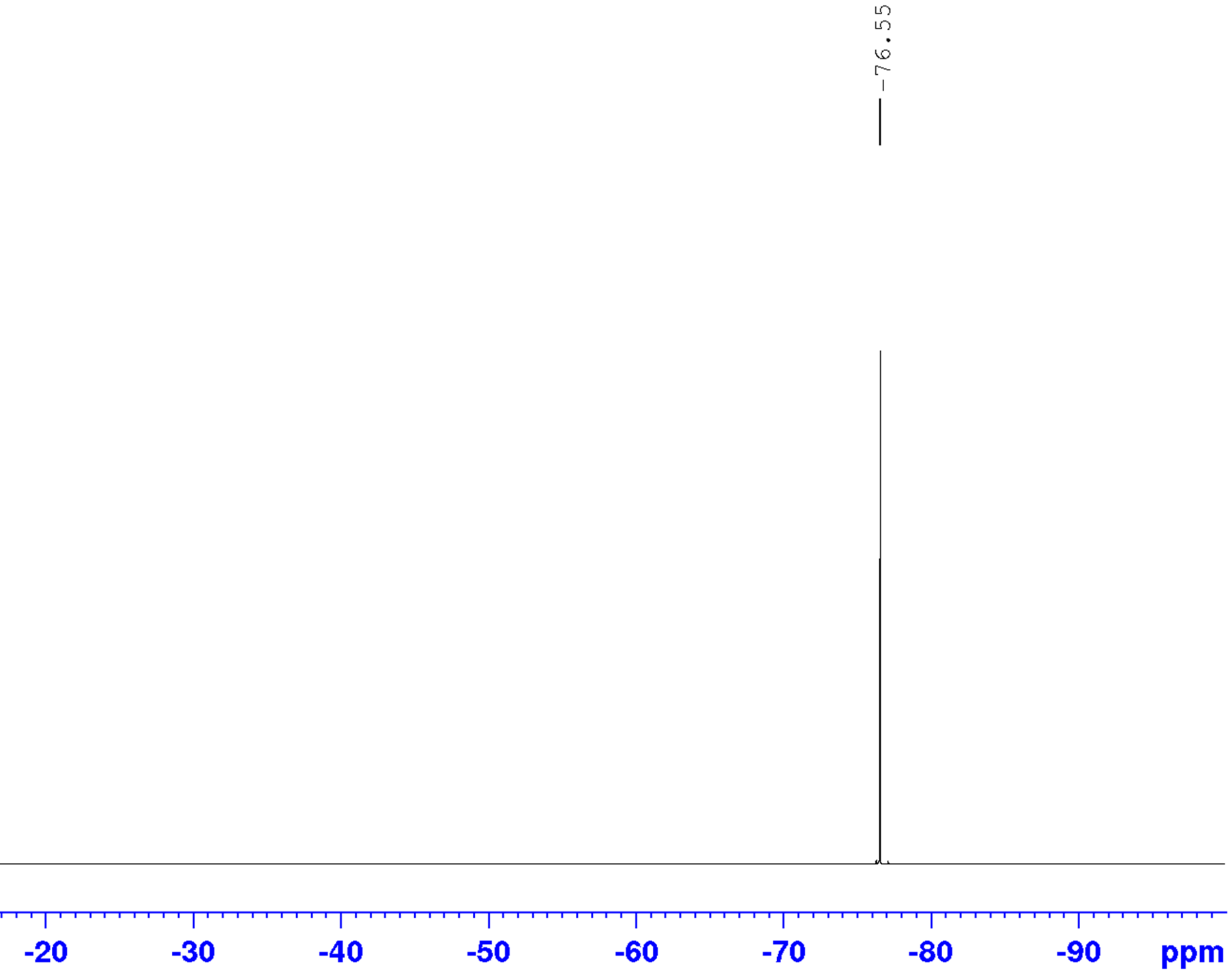

^{13}C NMR spectrum of **ZIL 3d-ene**

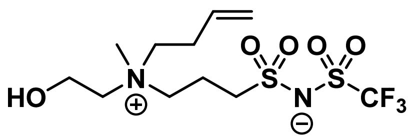

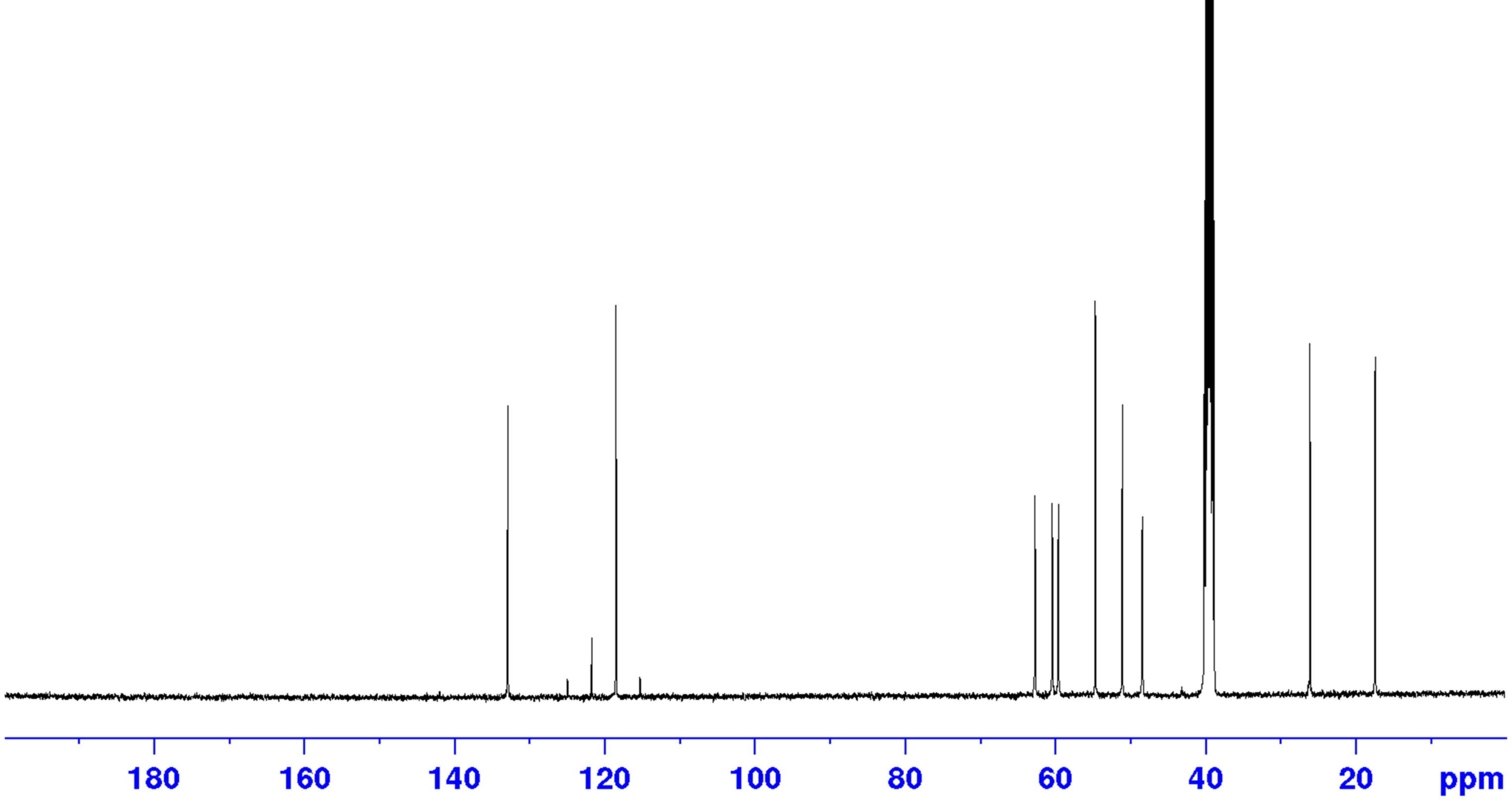

Mass spectrum of **ZIL 3d-ene**

Spectrum

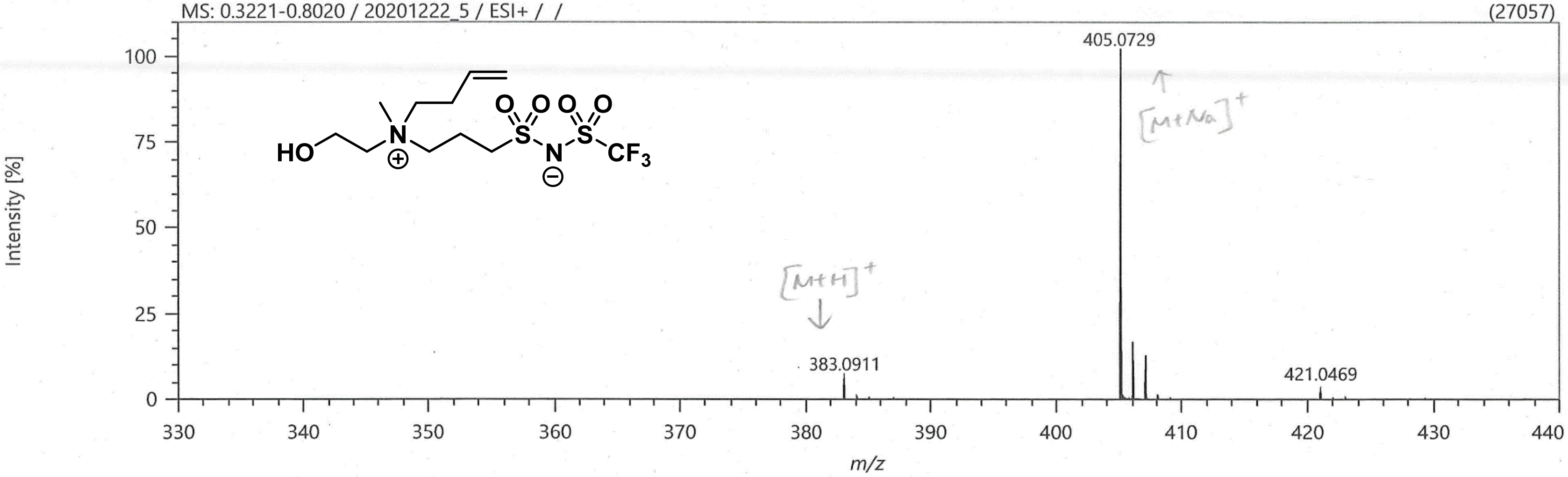

Elemental Composition

Parameters

		Elements Set 1:							
Tolerance:	±2.00 ppm	Symbol	C	H	F	N	O	S	Na
Electron:	Odd/Even	Min	0	0	3	2	5	2	0
Charge:	+1	Max	400	1000	3	2	5	2	1
DBE:	-99.0 - 999.0								

Results

Mass	Formula	Calculated Mass	Mass Difference [mDa]	Mass Difference [ppm]	DBE
383.09109	C11 H22 N2 O5 F3 S2	383.09167	-0.59	-1.53	0.5
405.07295	C11 H21 N2 O5 F3 Na S2	405.07362	-0.67	-1.66	0.5

^{1}H NMR spectrum of **ZIL 3d-yne**

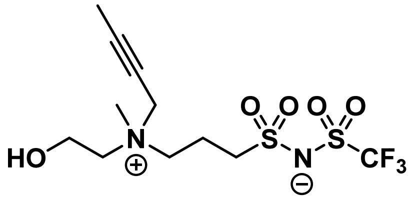

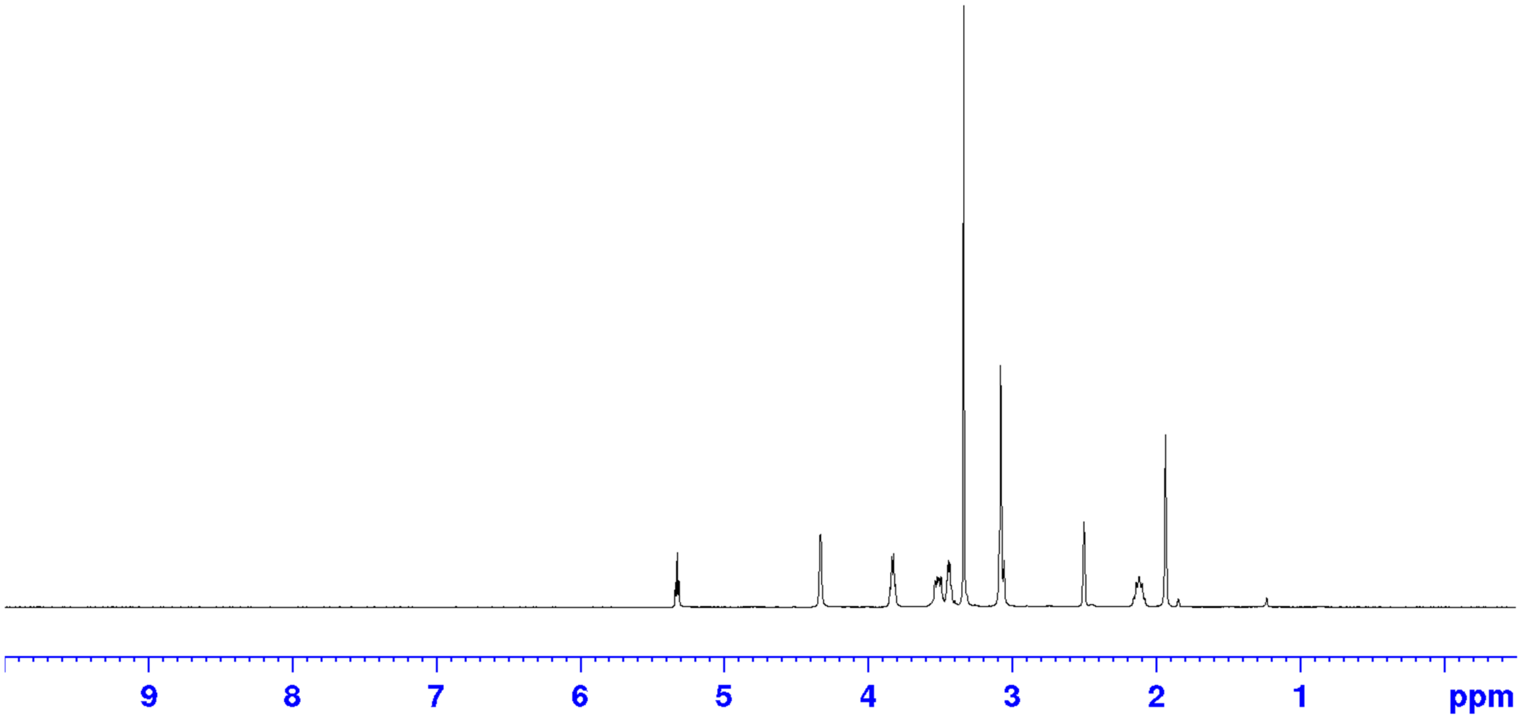

^{19}F NMR spectrum of **ZIL 3d-yne**

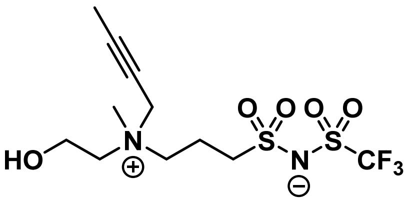

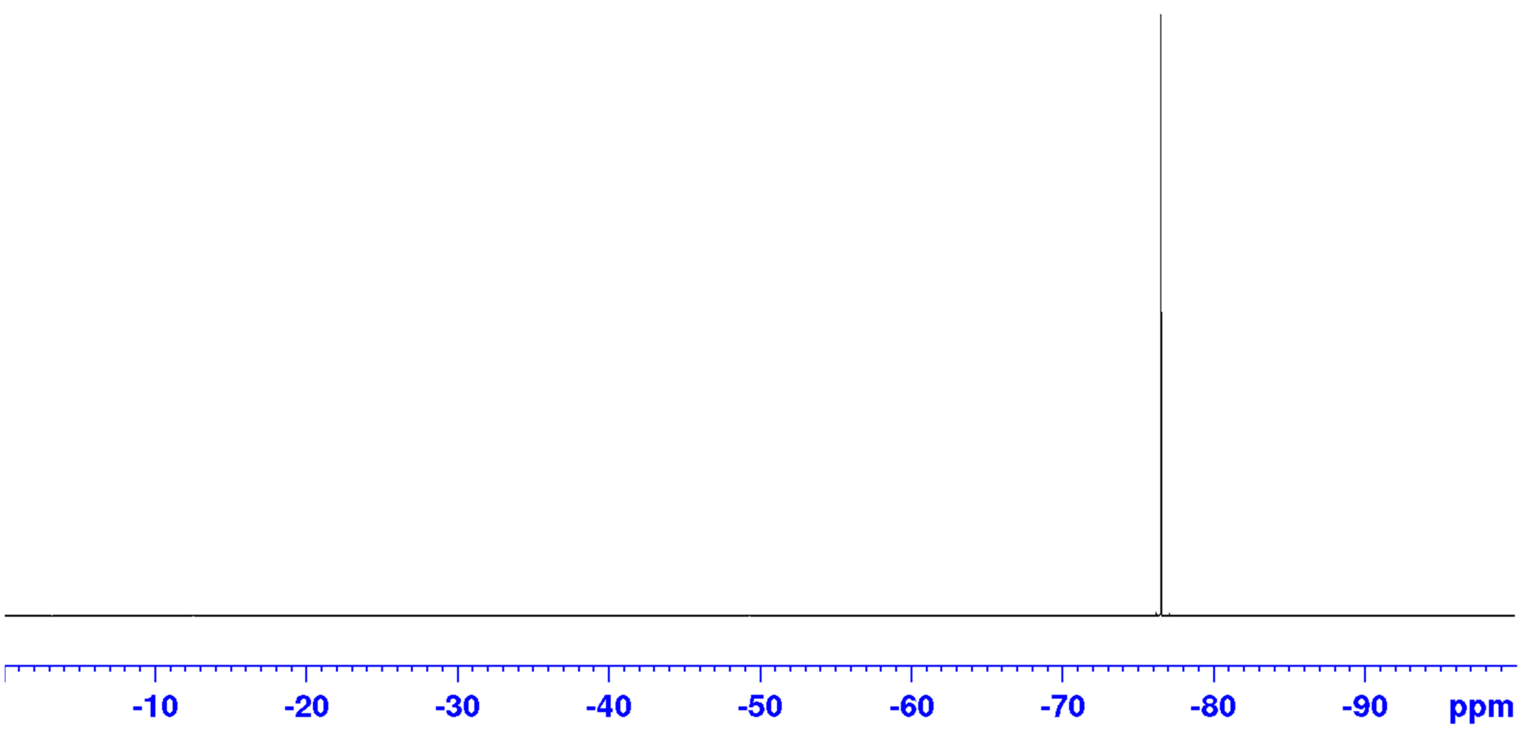

^{13}C NMR spectrum of **ZIL 3d-yne**

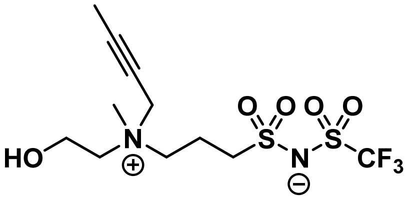

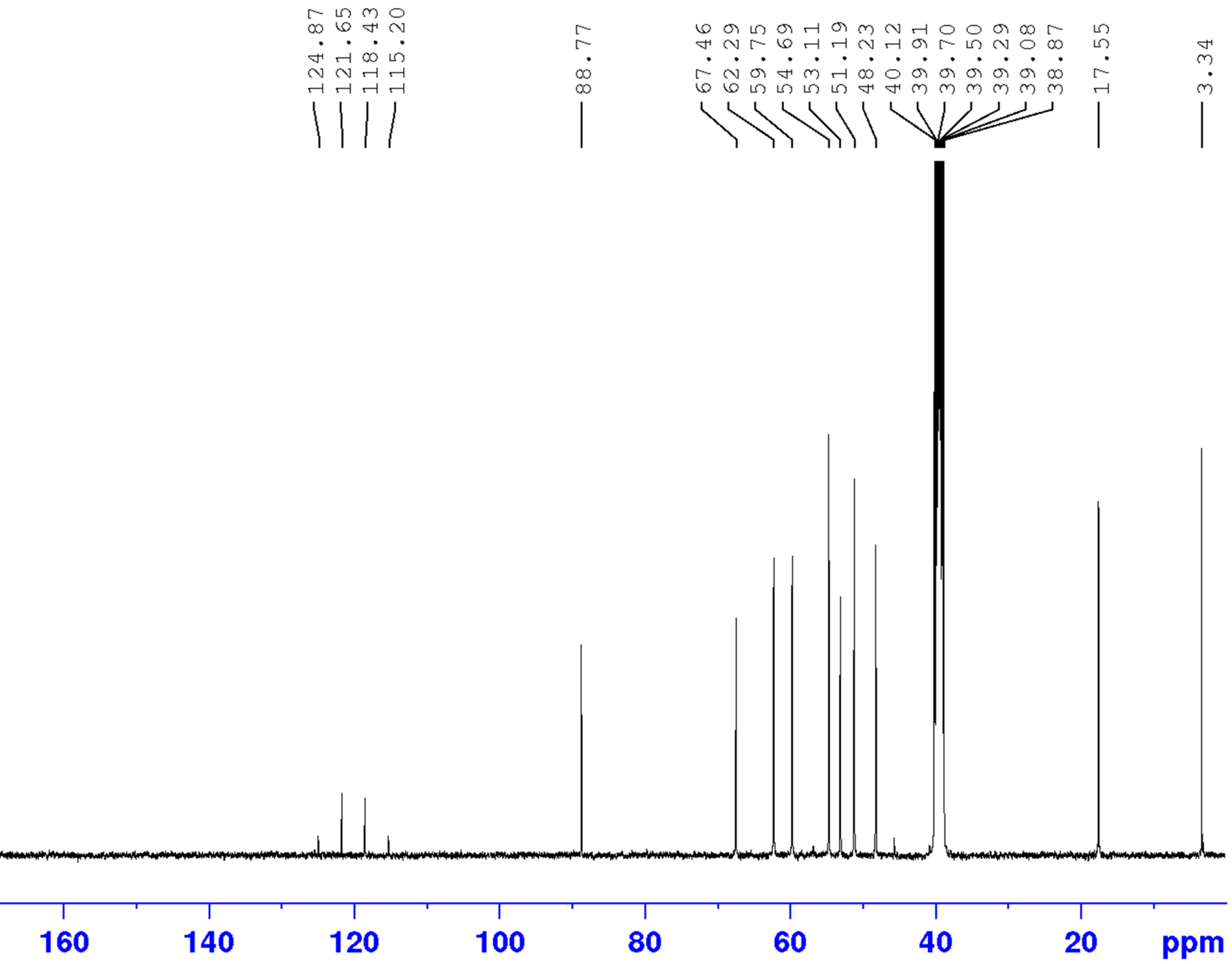

Mass spectrum of **ZIL 3d-yne**

Spectrum

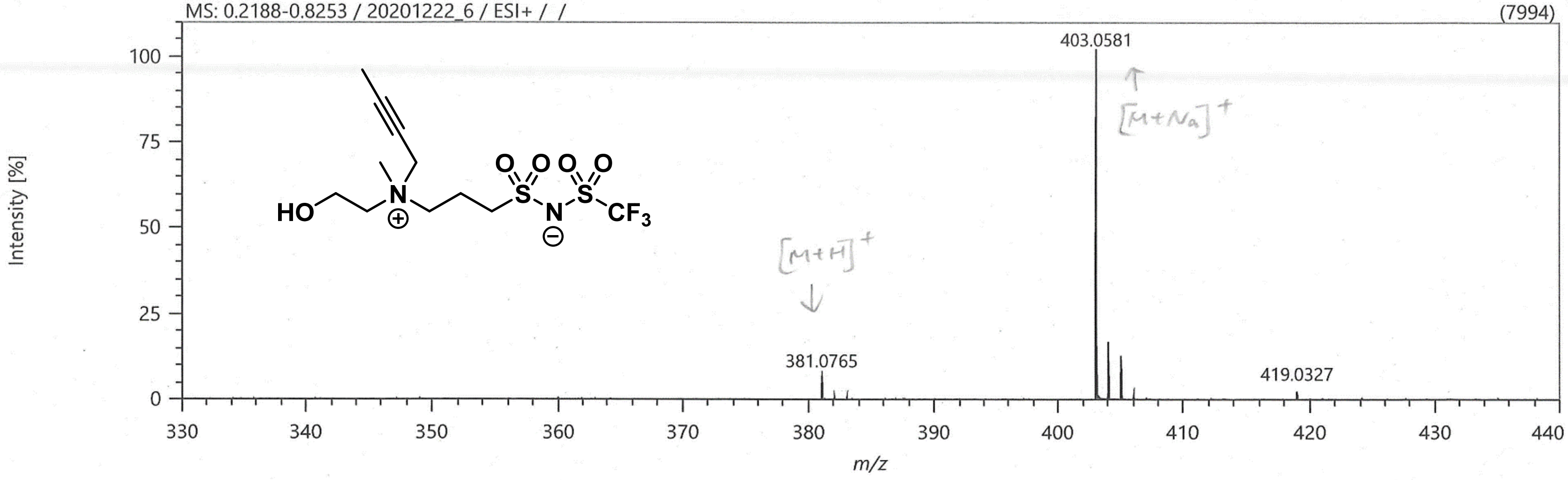

Elemental Composition

Parameters		Elements Set 1:							
Tolerance:	±2.00 ppm	Symbol	C	H	F	N	O	S	Na
Electron:	Odd/Even	Min	0	0	3	2	5	2	0
Charge:	+1	Max	400	1000	3	2	5	2	1
DBE:	-99.0 - 999.0								

Results

Mass	Formula	Calculated Mass	Mass Difference [mDa]	Mass Difference [ppm]	DBE
381.07652	C11 H20 N2 O5 F3 S2	381.07602	0.50	1.30	1.5
403.05810	C11 H19 N2 O5 F3 Na S2	403.05797	0.13	0.32	1.5

^{1}H NMR spectrum of **ZIL 3e**

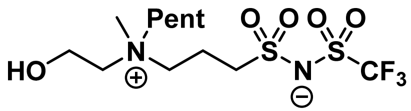

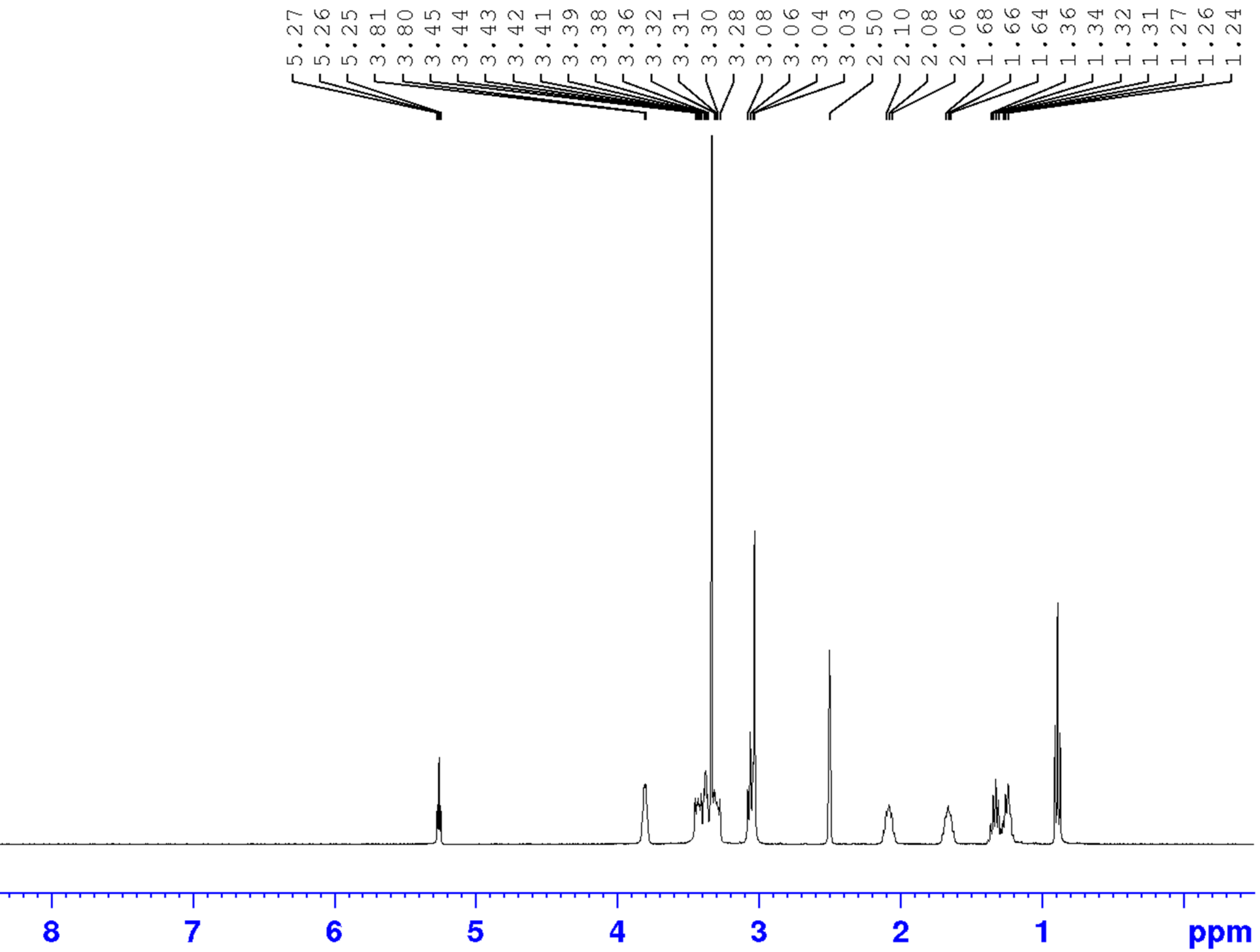

^{19}F NMR spectrum of **ZIL 3e**

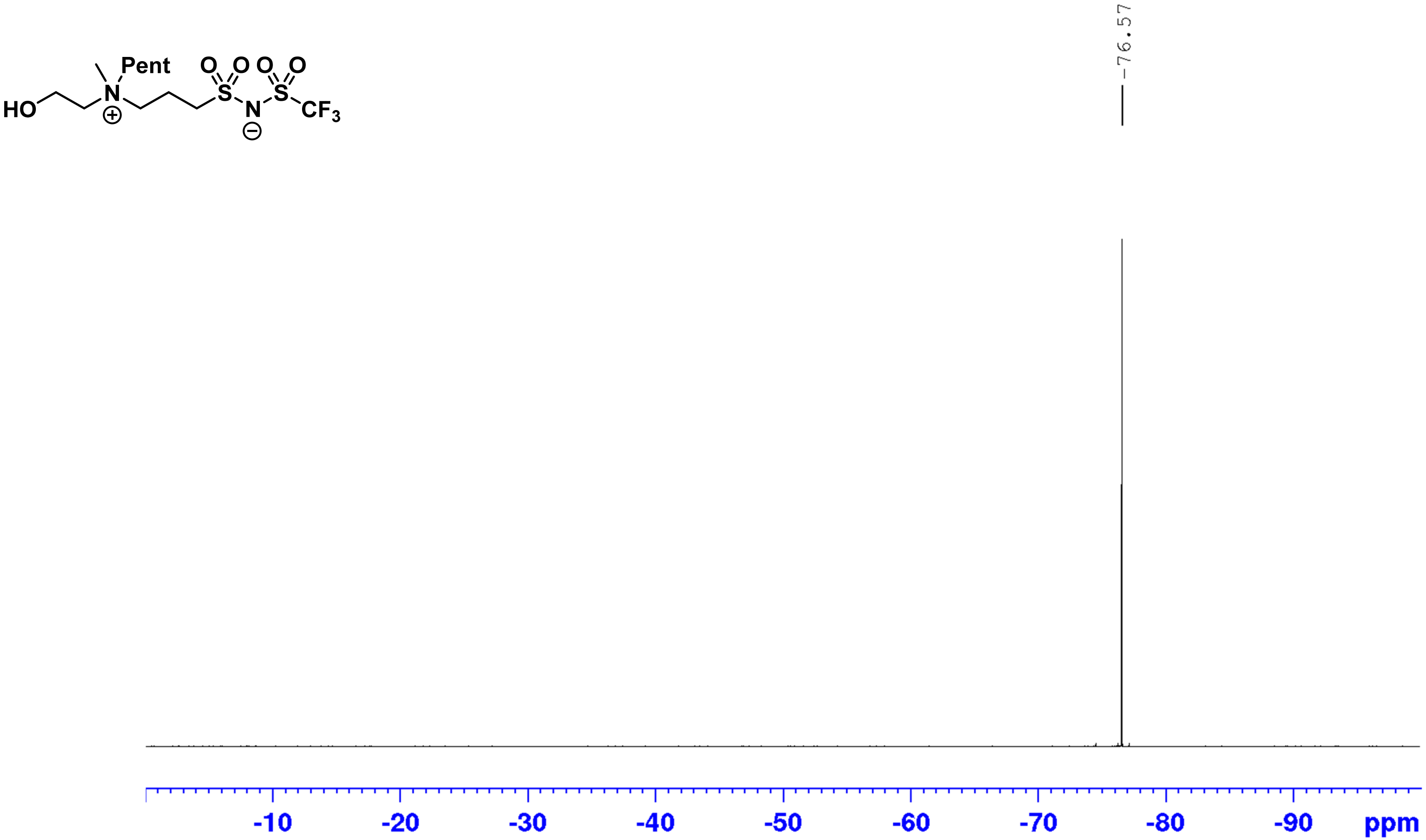

^{13}C NMR spectrum of **ZIL 3e**

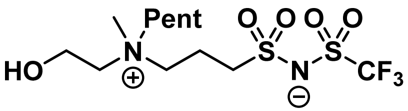

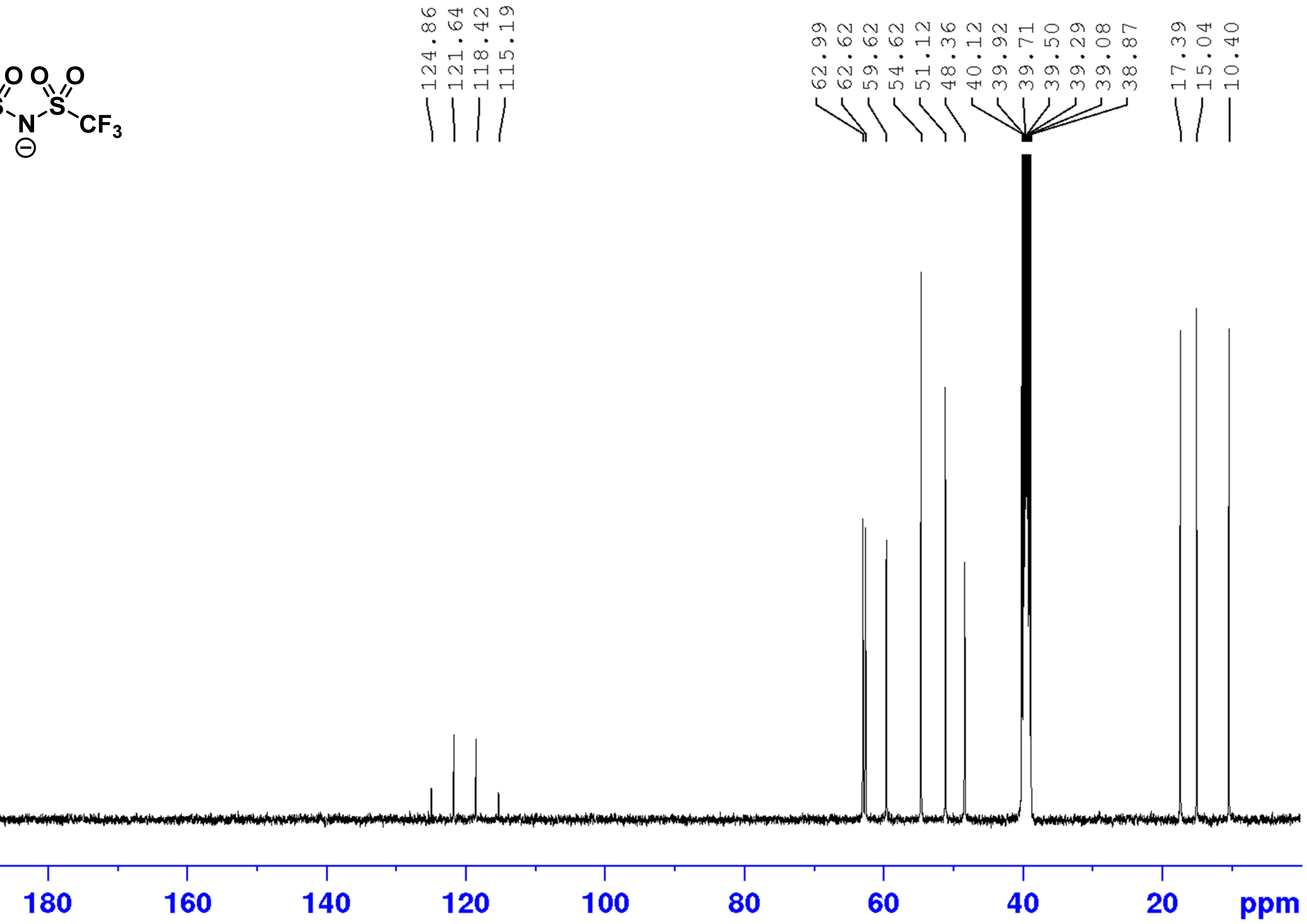

Mass spectrum of **ZIL 3e**

Spectrum

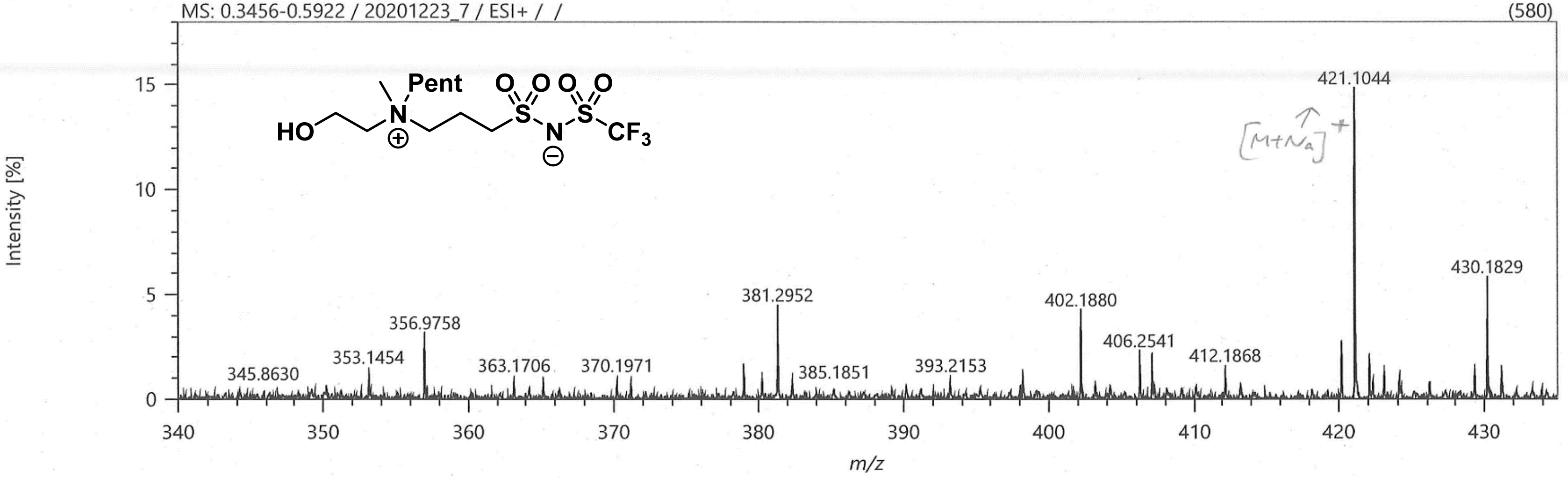

Elemental Composition

Parameters	
Tolerance:	±2.00 ppm
Electron:	Odd/Even
Charge:	+1
DBE:	-99.0 - 999.0

Elements Set 1:

Symbol	C	H	F	N	O	S	Na
Min	0	0	3	2	5	2	0
Max	400	1000	3	2	5	2	1

Results

Mass	Formula	Calculated Mass	Mass Difference [mDa]	Mass Difference [ppm]	DBE
398.17931					
421.10436	C12 H25 N2 O5 F3 Na S2	421.10492	-0.55	-1.32	-0.5

^{1}H NMR spectrum of **ZIL 3f**

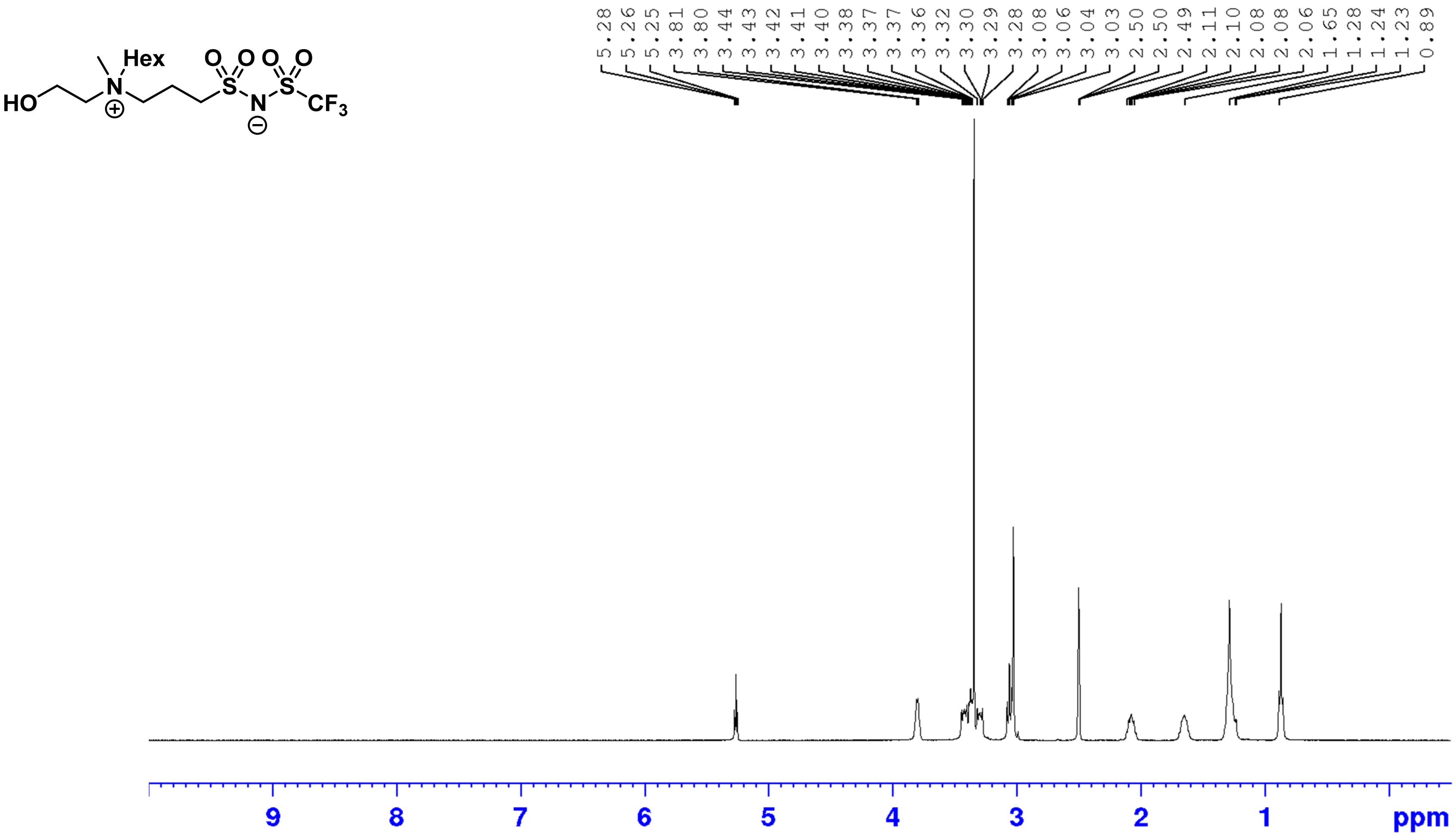

^{19}F NMR spectrum of **ZIL 3f**

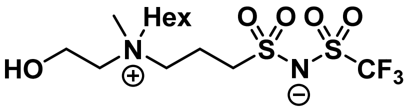

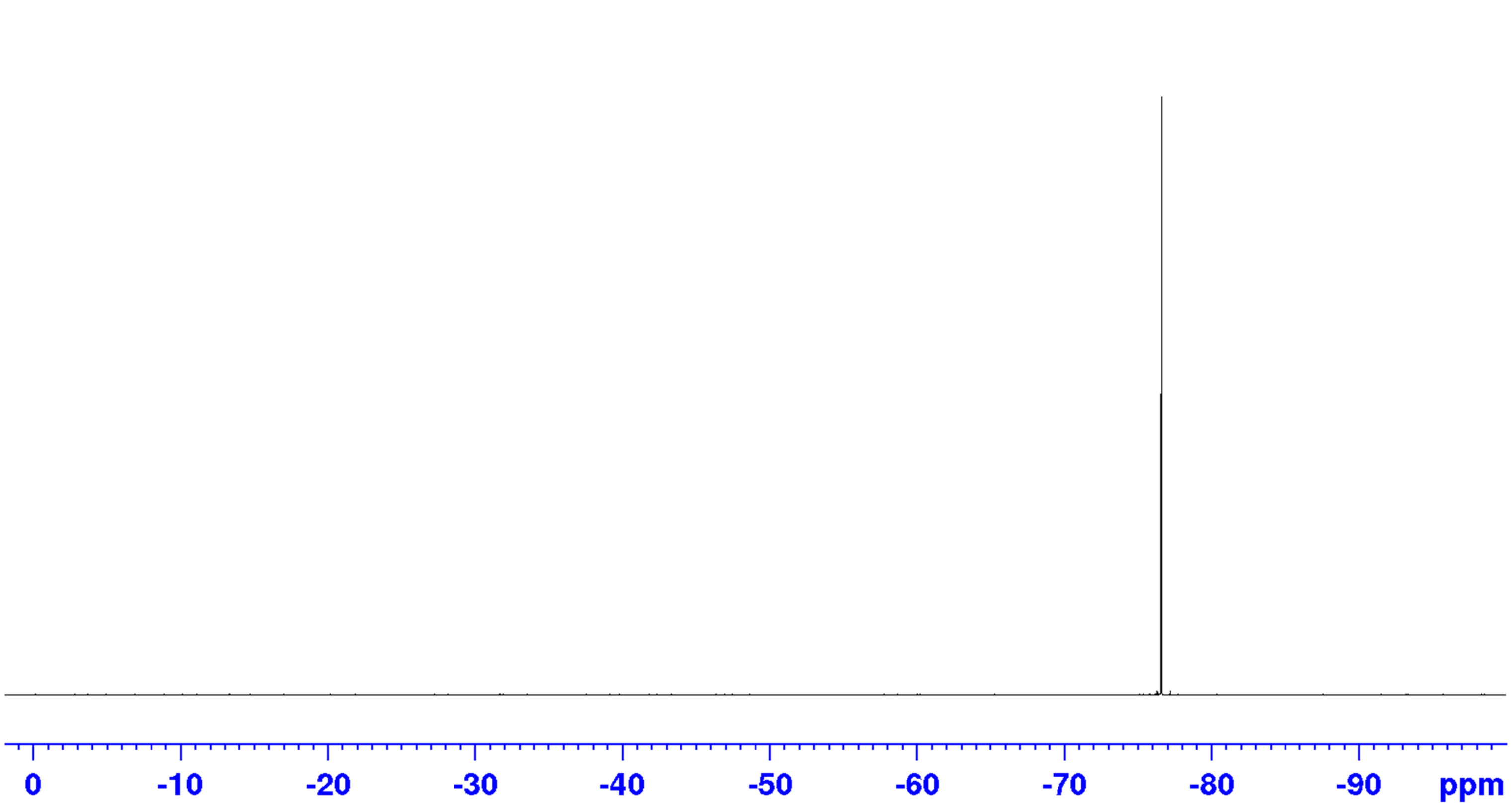

^{13}C NMR spectrum of **ZIL 3f**

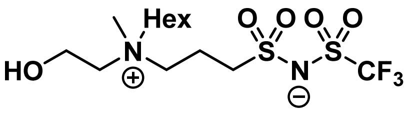

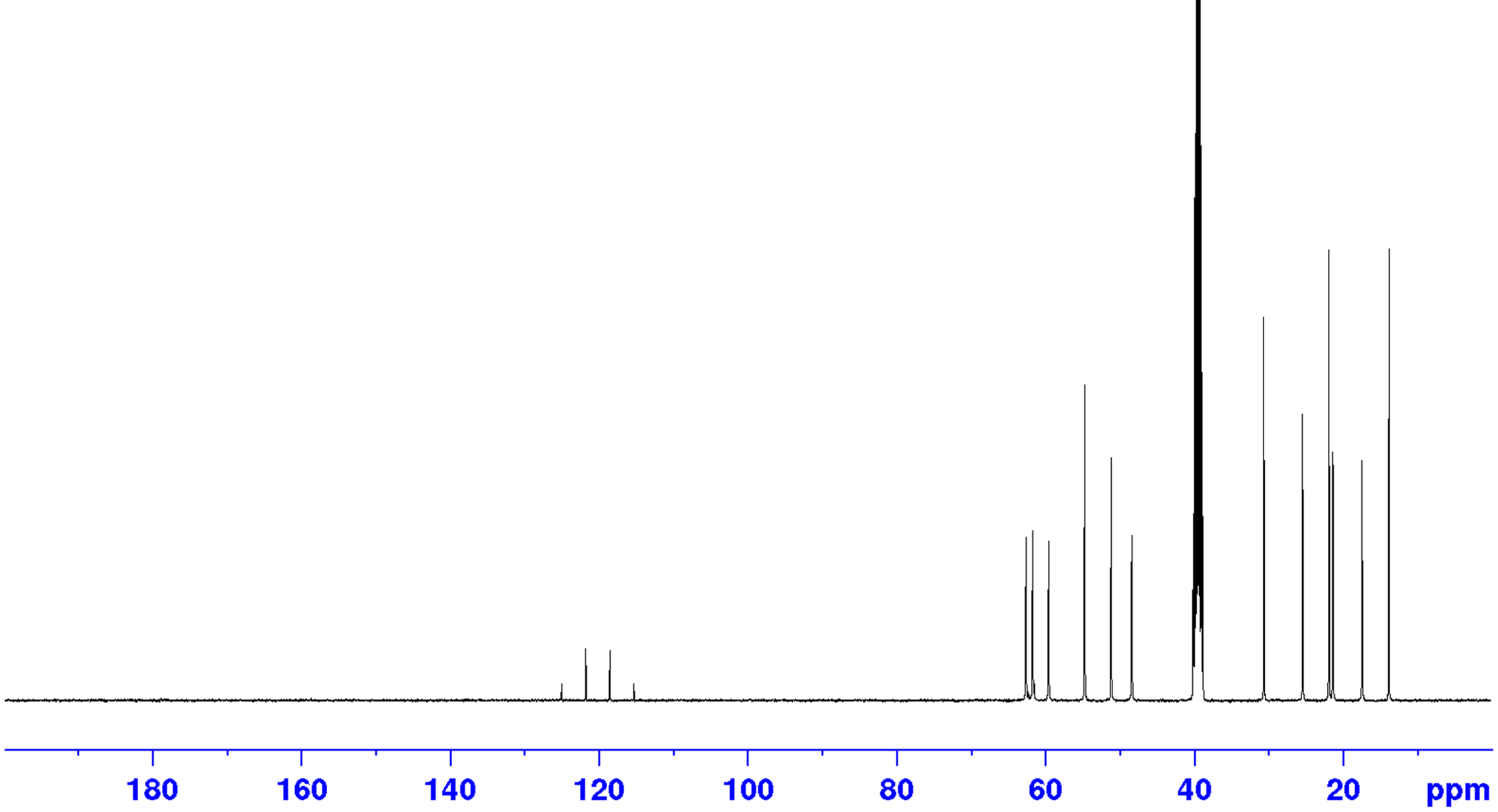

Mass spectrum of **ZIL 3f**

Spectrum

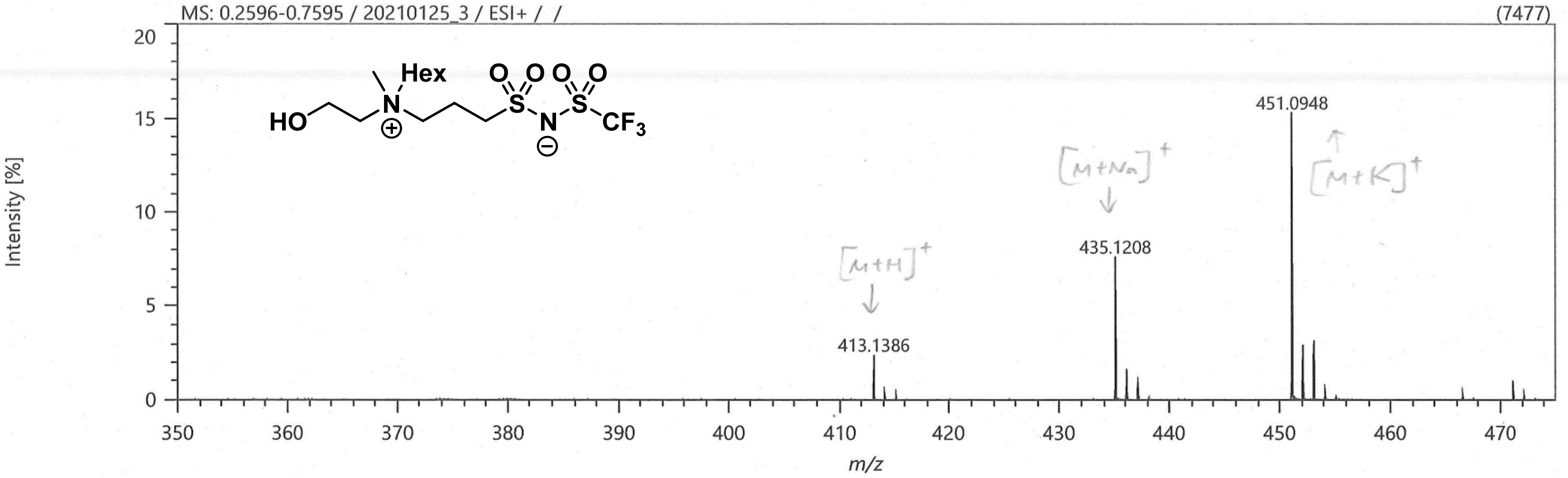

Elemental Composition

Parameters

Tolerance:	±3.00 ppm
Electron:	Odd/Even
Charge:	+1
DBE:	-99.0 - 999.0

Elements Set 1:

Symbol	C	H	N	O	S	F	Na	K
Min	0	0	2	5	2	3	0	0
Max	400	1000	2	5	2	3	1	1

Results

Mass	Formula	Calculated Mass	Mass Difference [mDa]	Mass Difference [ppm]	DBE
413.13861	C13 H28 N2 O5 F3 S2	413.13862	-0.01	-0.02	-0.5
435.12075	C13 H27 N2 O5 F3 Na S2	435.12057	0.19	0.43	-0.5
451.09483	C13 H27 N2 O5 F3 S2 K	451.09451	0.33	0.73	-0.5

^{1}H NMR spectrum of **ZIL 4a**

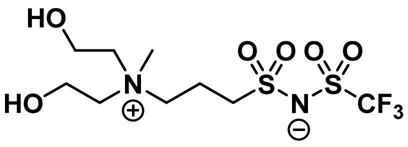

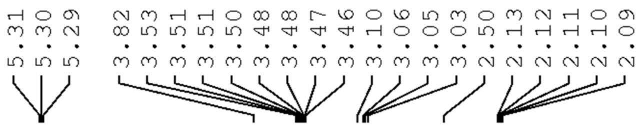

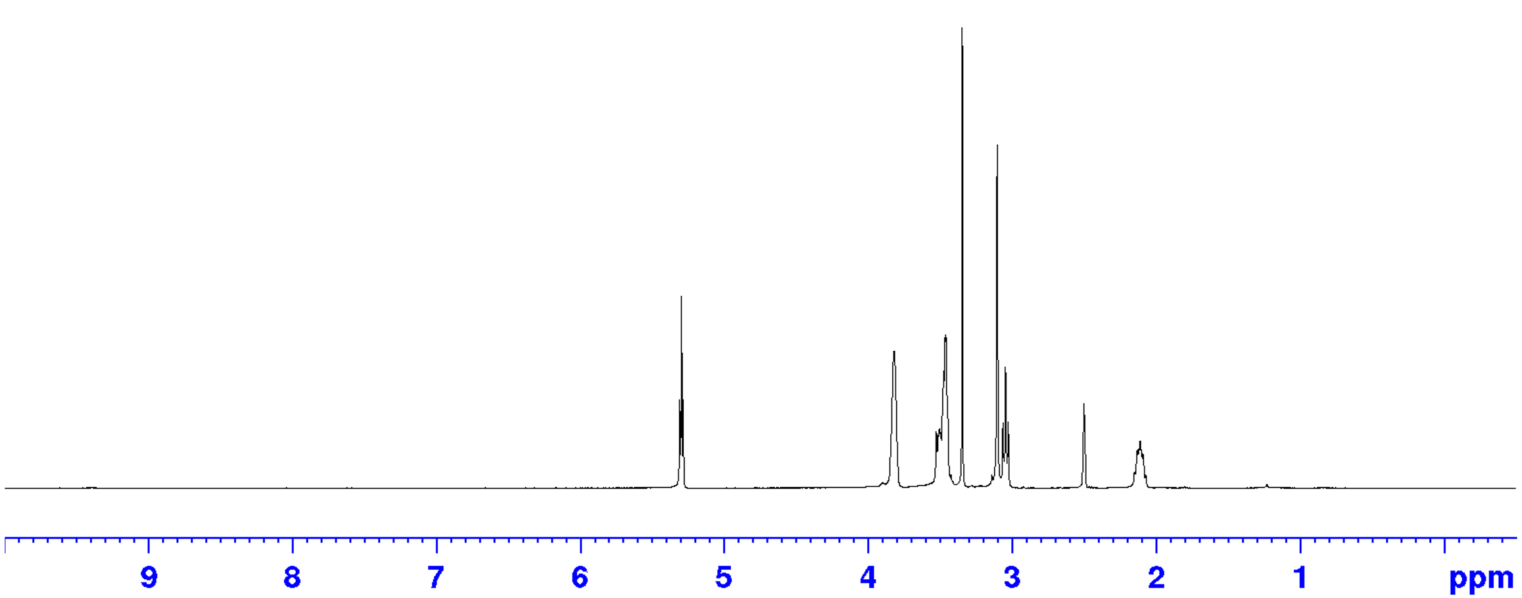

^{19}F NMR spectrum of **ZIL 4a**

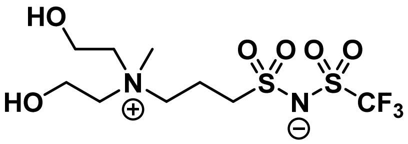

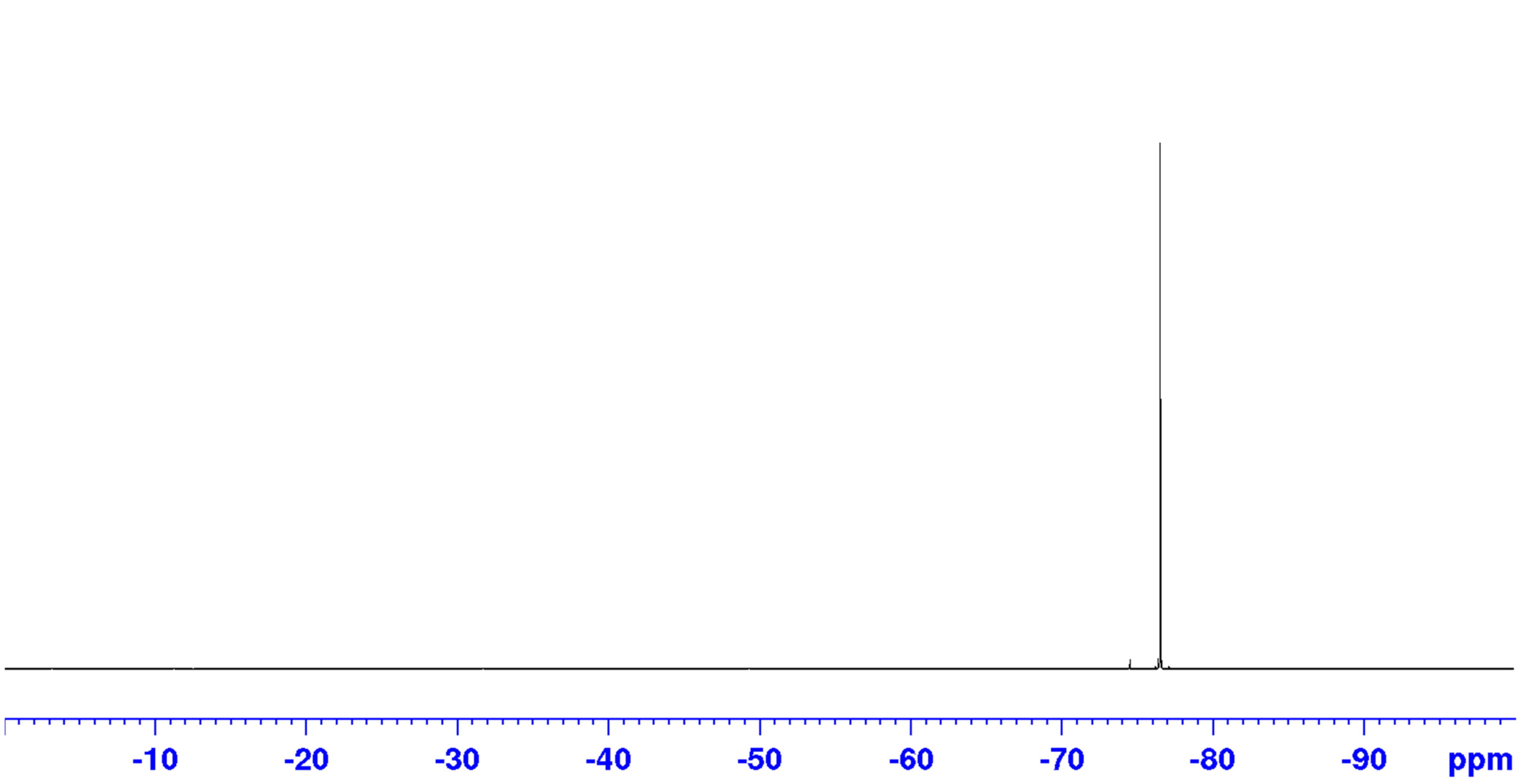

^{13}C NMR spectrum of **ZIL 4a**

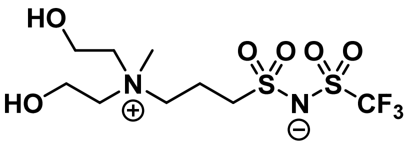

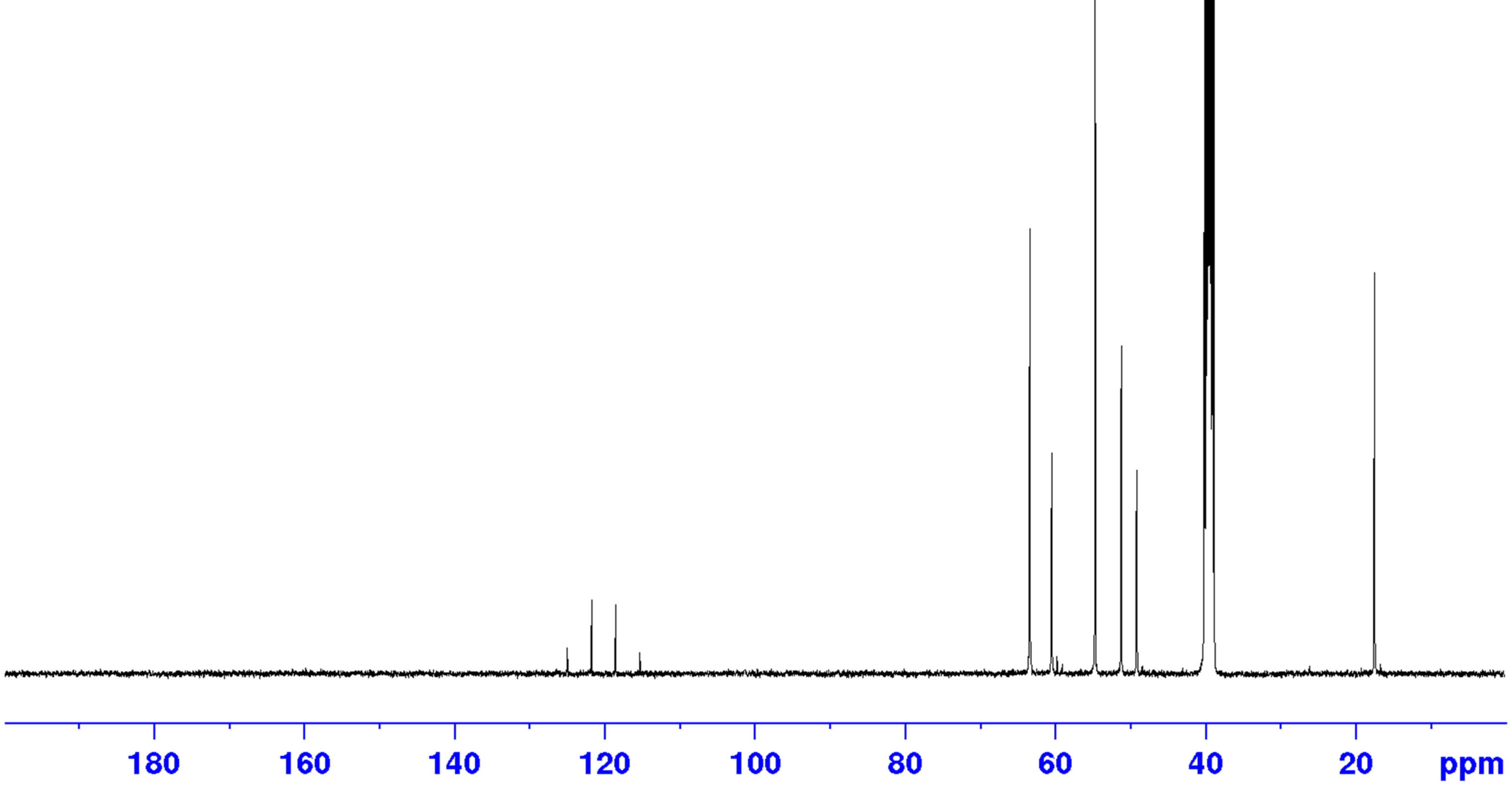

Mass spectrum of **ZIL 4a**

Spectrum

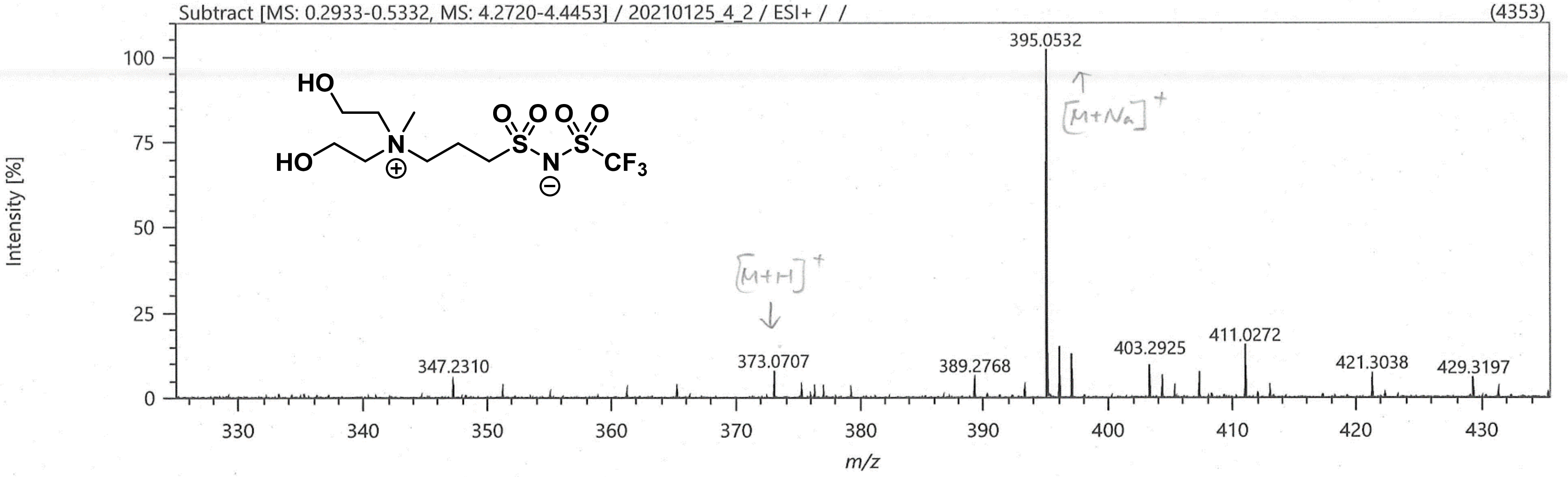

Elemental Composition

Parameters		Elements Set 1:							
Tolerance:	±2.00 ppm	Symbol	C	H	N	O	S	F	Na
Electron:	Odd/Even	Min	0	0	2	6	2	3	0
Charge:	+1	Max	400	1000	2	6	2	3	1
DBE:	-99.0 - 999.0								

Results

Mass	Formula	Calculated Mass	Mass Difference [mDa]	Mass Difference [ppm]	DBE
373.07066	C9 H20 N2 O6 F3 S2	373.07094	-0.27	-0.74	-0.5
395.05320	C9 H19 N2 O6 F3 Na S2	395.05288	0.32	0.81	-0.5

^{1}H NMR spectrum of **ZIL 4b**

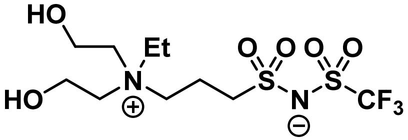

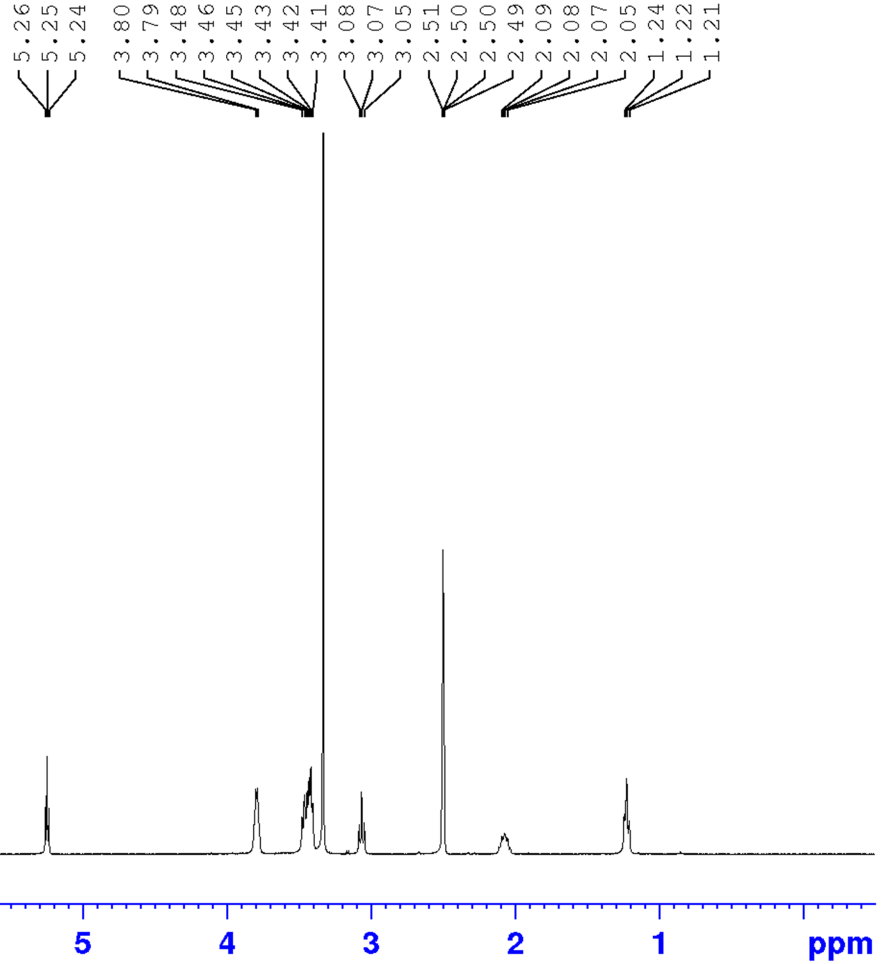

^{19}F NMR spectrum of **ZIL 4b**

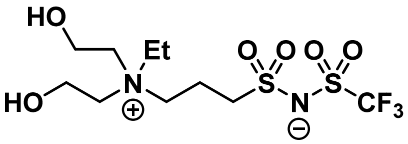

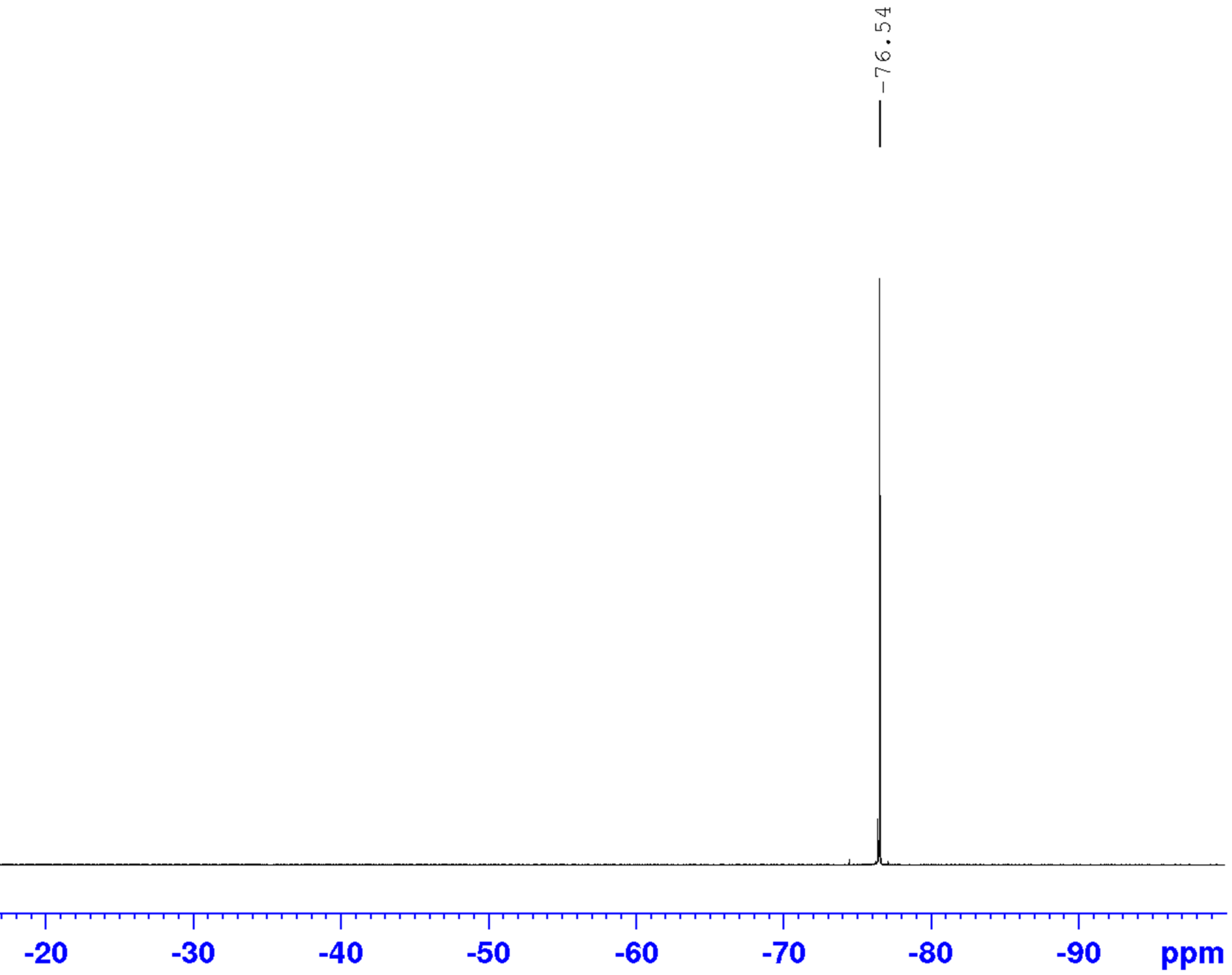

^{13}C NMR spectrum of **ZIL 4b**

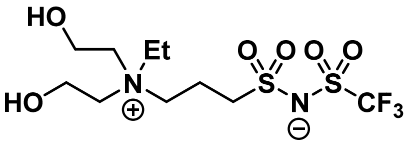

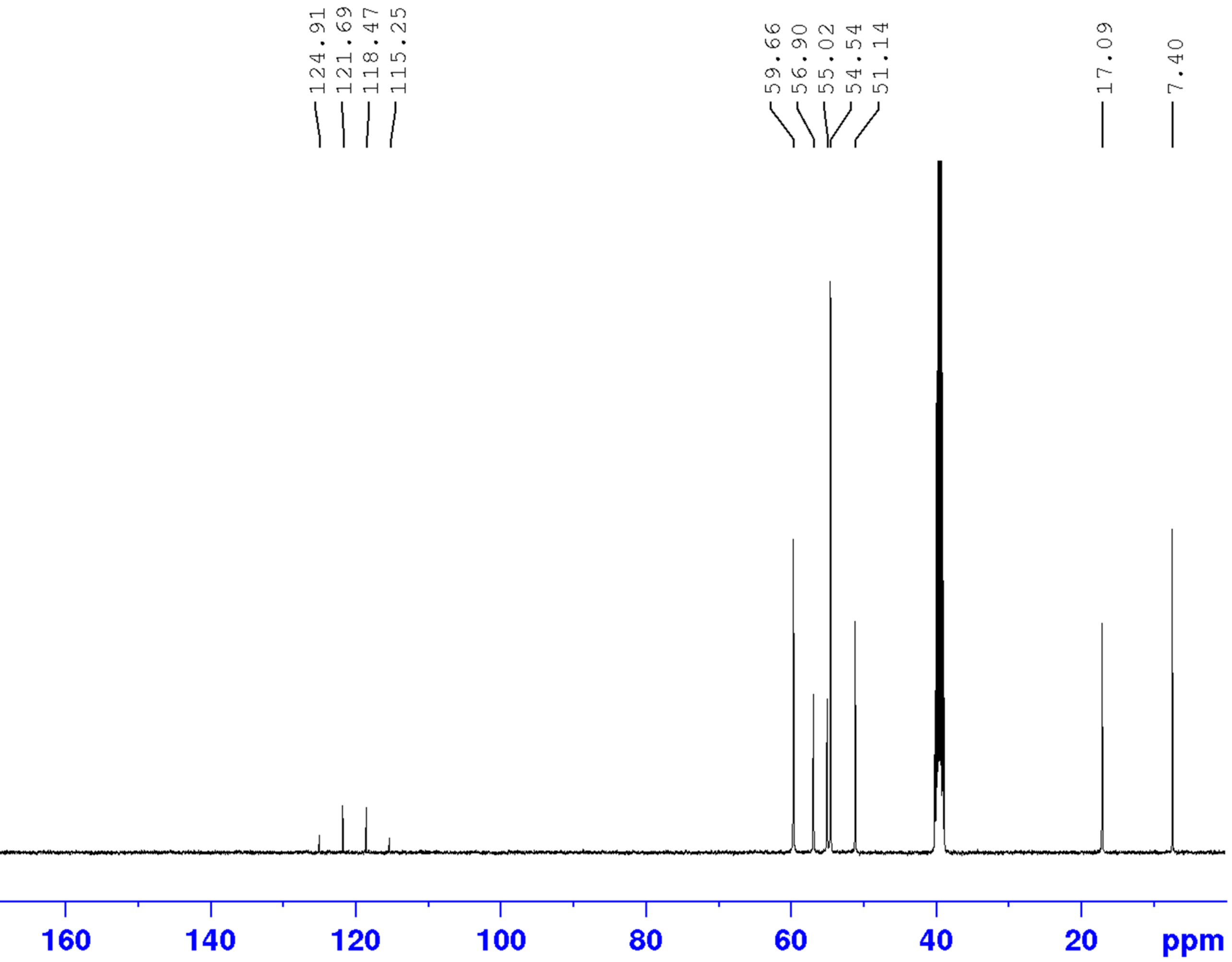

Mass spectrum of **ZIL 4b**

Spectrum

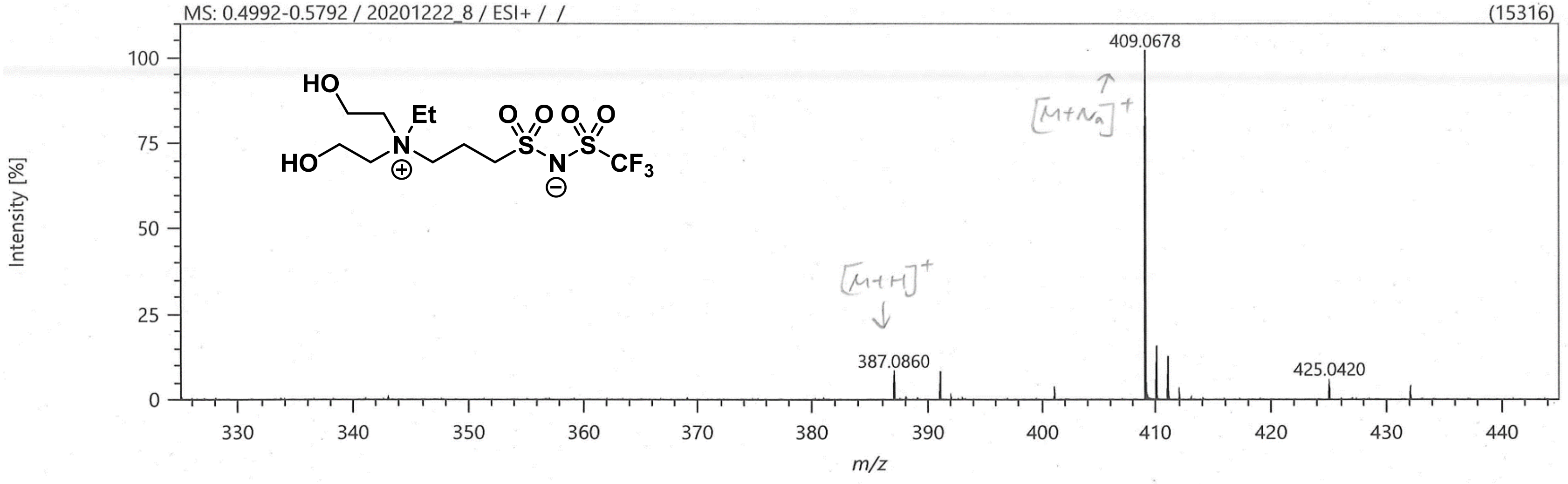

Elemental Composition

Parameters		Elements Set 1:							
Tolerance:	±2.00 ppm	Symbol	C	H	F	N	O	S	Na
Electron:	Odd/Even	Min	0	0	3	2	6	2	0
Charge:	+1	Max	400	1000	3	2	6	2	1
DBE:	-99.0 - 999.0								

Results

Mass	Formula	Calculated Mass	Mass Difference [mDa]	Mass Difference [ppm]	DBE
387.08598	C10 H22 N2 O6 F3 S2	387.08659	-0.61	-1.57	-0.5
409.06784	C10 H21 N2 O6 F3 Na S2	409.06853	-0.69	-1.69	-0.5

^{1}H NMR spectrum of **ZIL 4c**

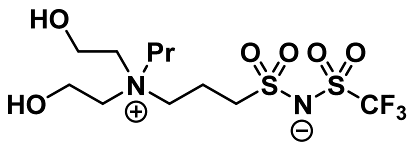

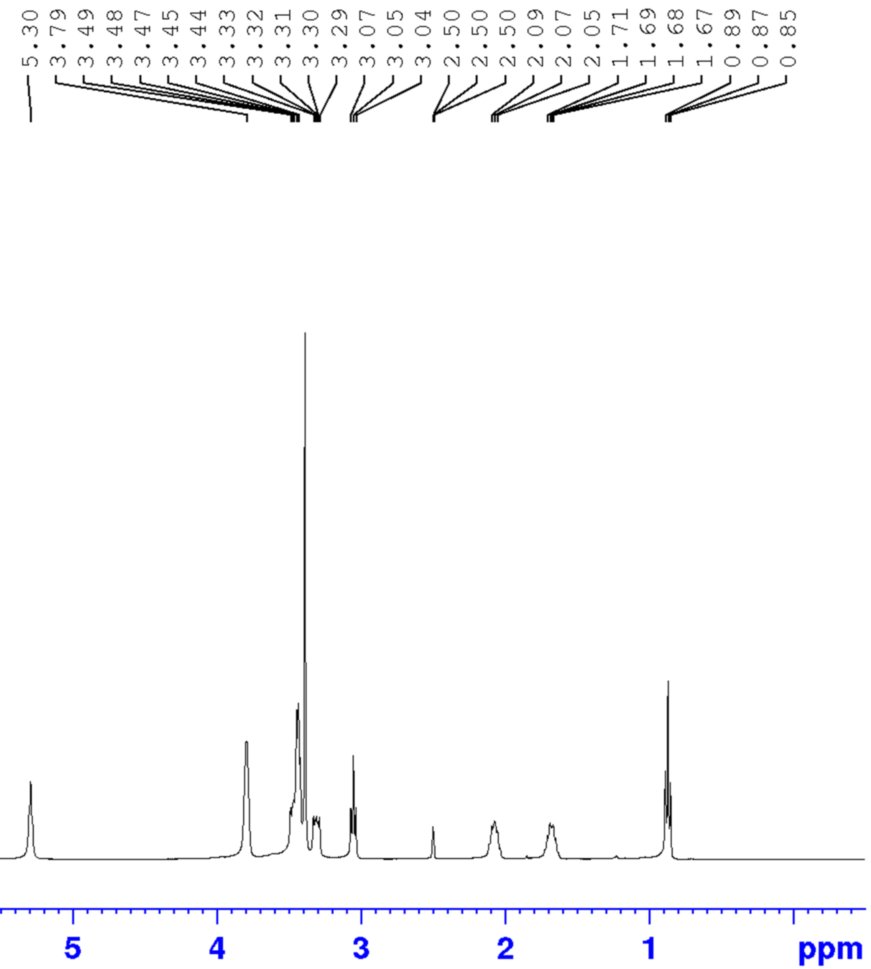

^{19}F NMR spectrum of **ZIL 4c**

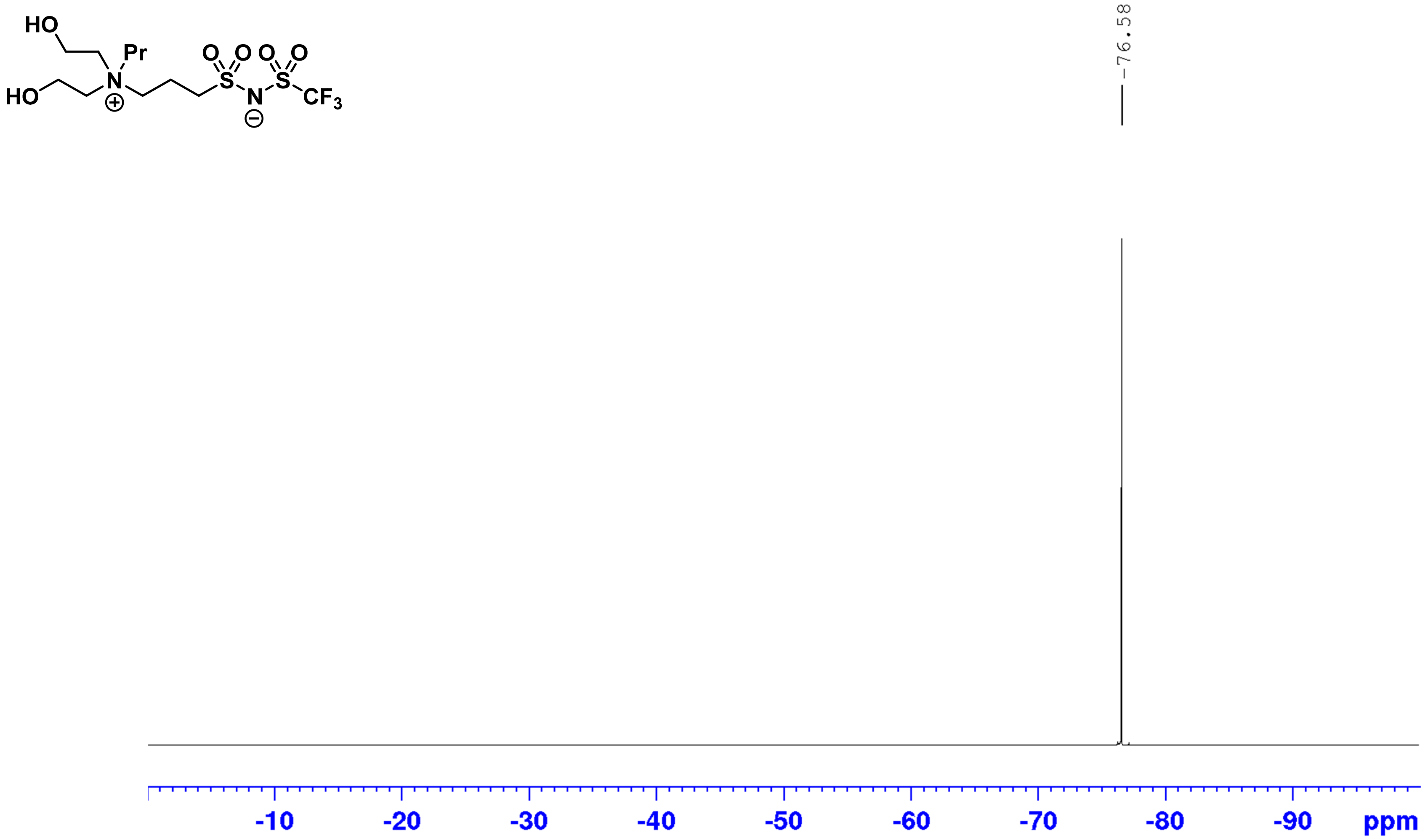

^{13}C NMR spectrum of **ZIL 4c**

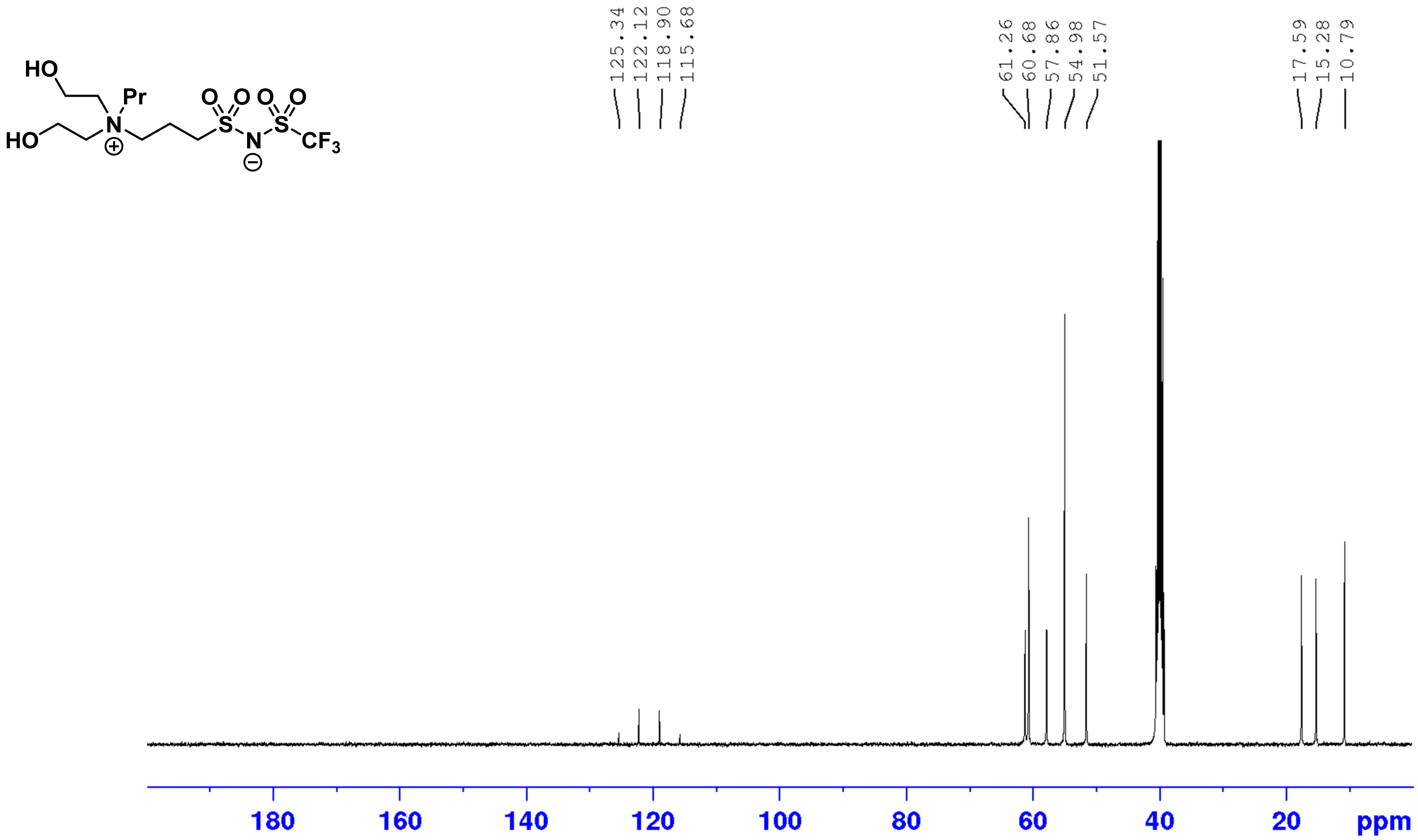

Mass spectrum of **ZIL 4c**

Spectrum

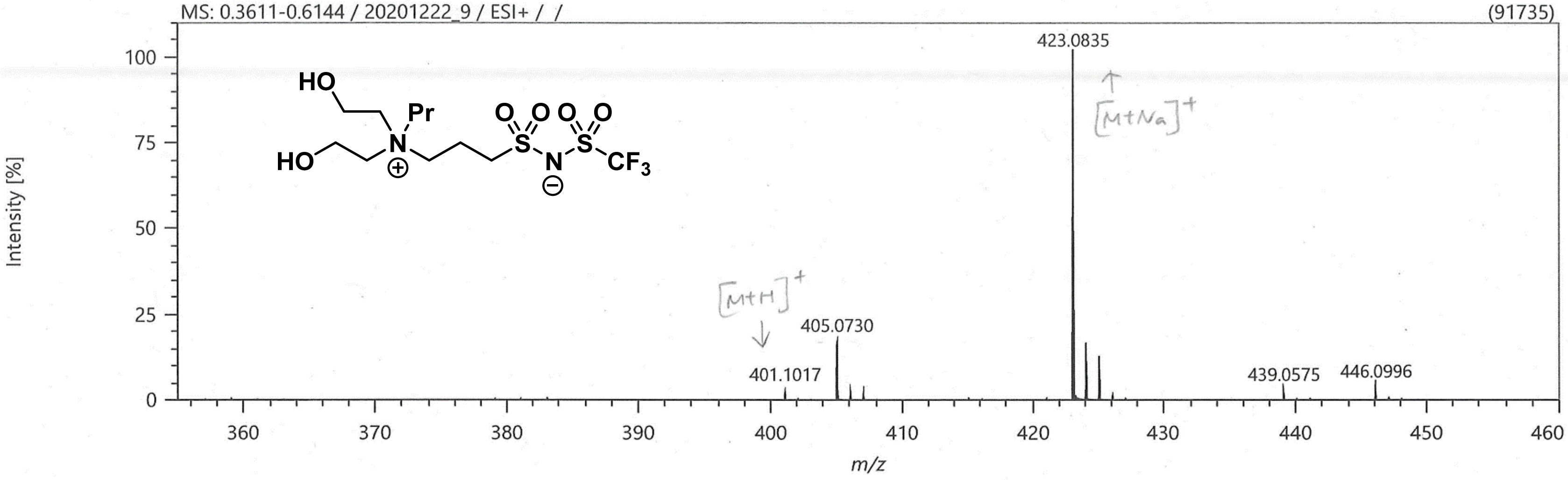

Elemental Composition

Parameters		Elements Set 1:							
Tolerance:	±2.00 ppm	Symbol	C	H	F	N	O	S	Na
Electron:	Odd/Even	Min	0	0	3	2	6	2	0
Charge:	+1	Max	400	1000	3	2	6	2	1
DBE:	-99.0 - 999.0								

Results

Mass	Formula	Calculated Mass	Mass Difference [mDa]	Mass Difference [ppm]	DBE
401.10167	C11 H24 N2 O6 F3 S2	401.10224	-0.57	-1.43	-0.5
423.08348	C11 H23 N2 O6 F3 Na S2	423.08418	-0.70	-1.66	-0.5

^{1}H NMR spectrum of **ZIL 4d**

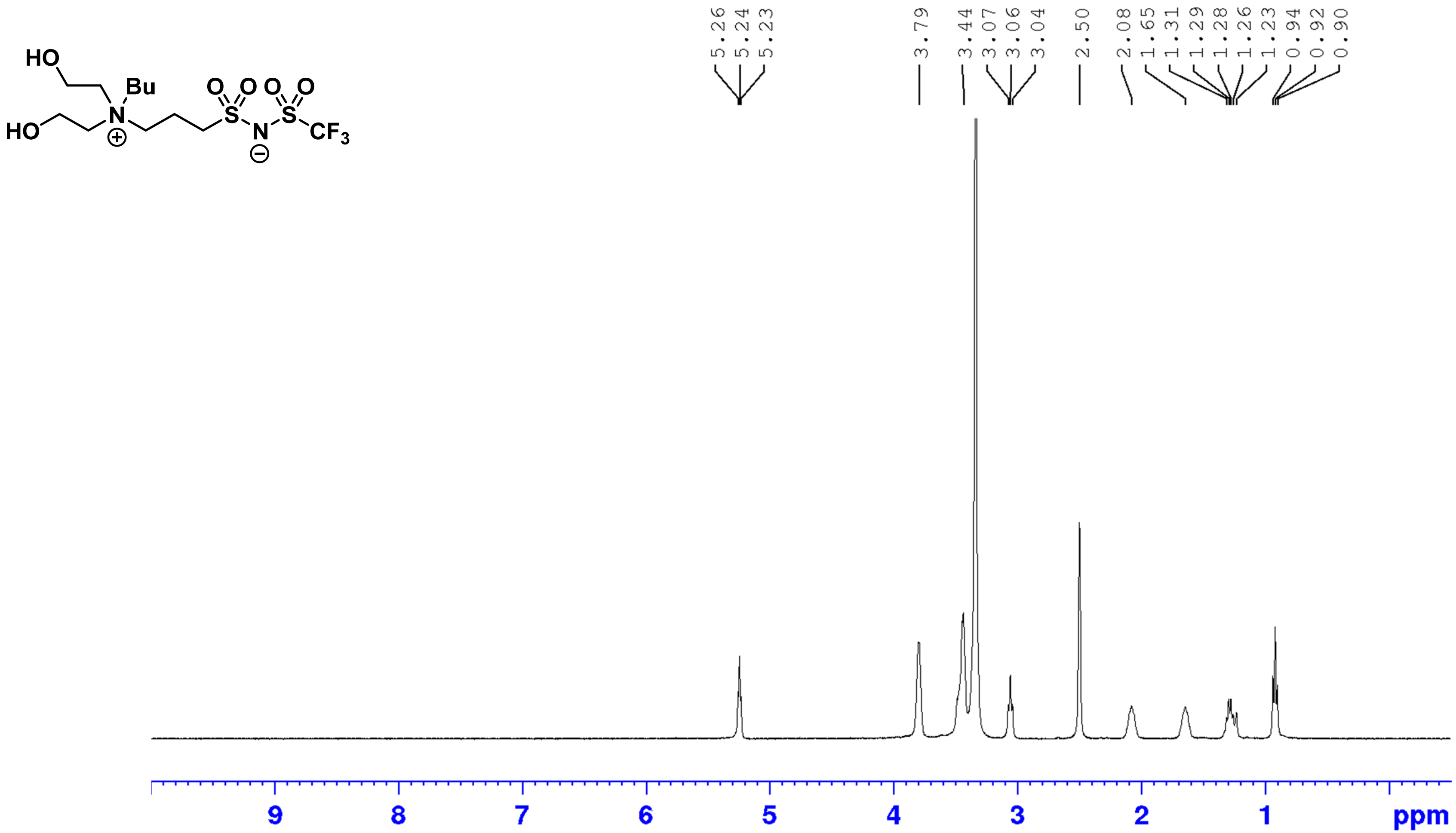

^{19}F NMR spectrum of **ZIL 4d**

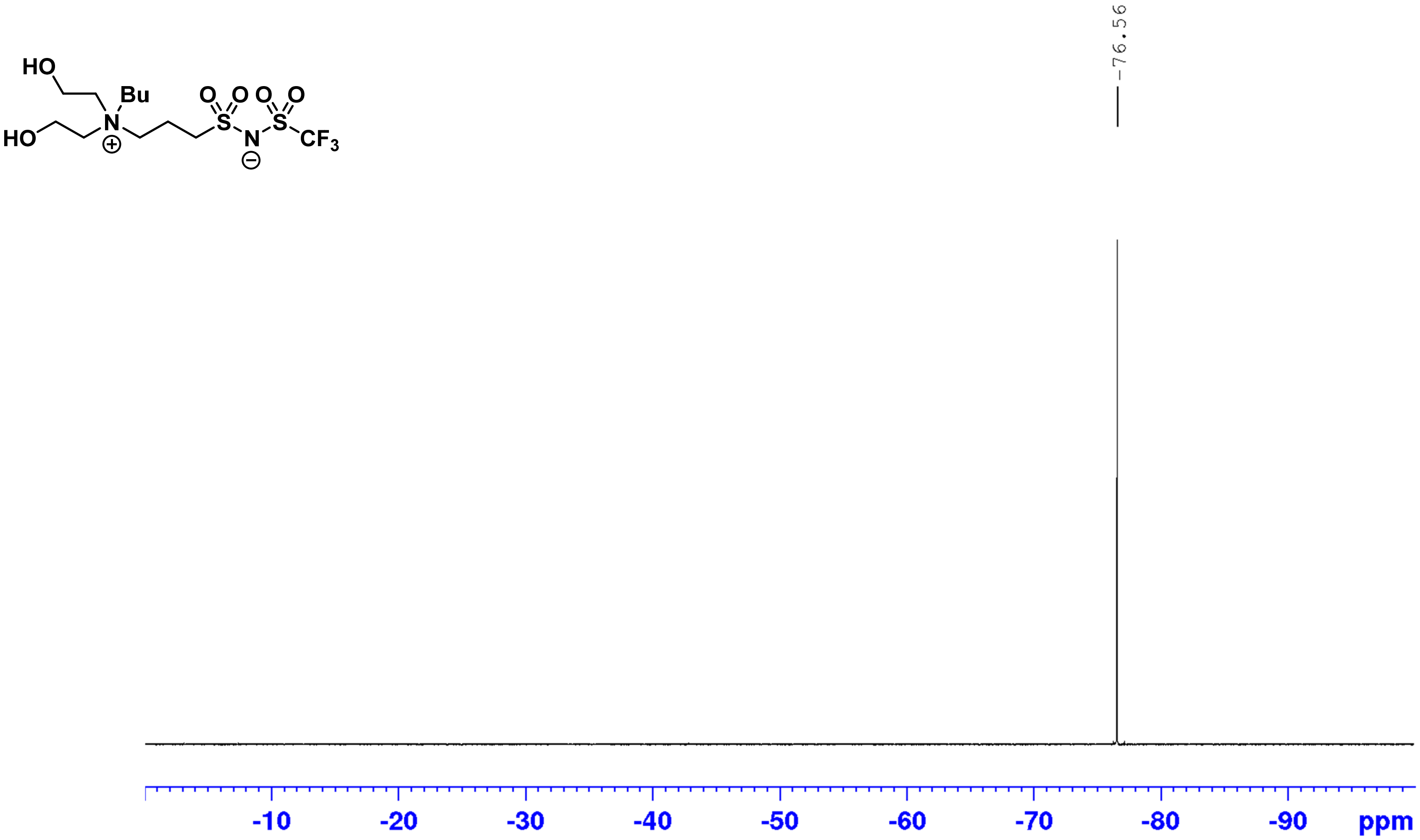

^{13}C NMR spectrum of **ZIL 4d**

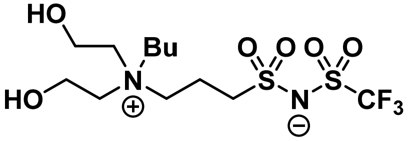

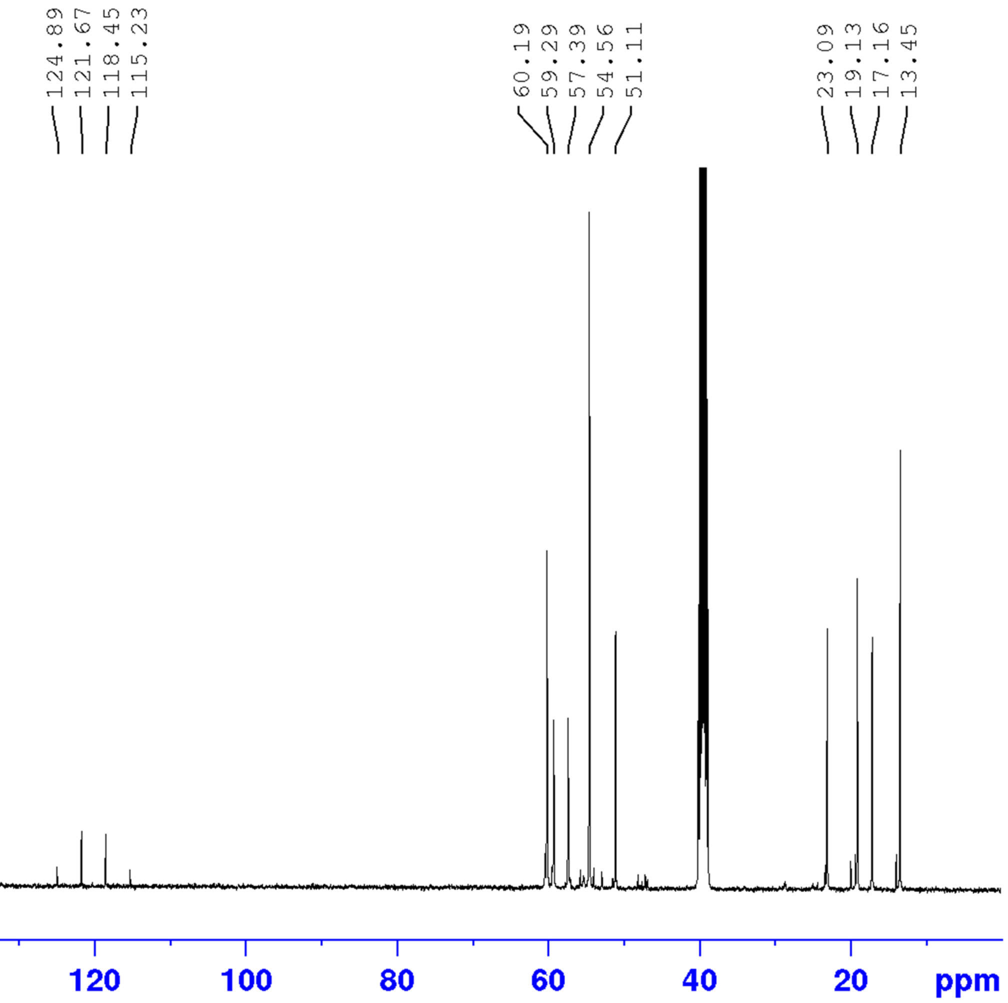

Mass spectrum of **ZIL 4d**

Spectrum

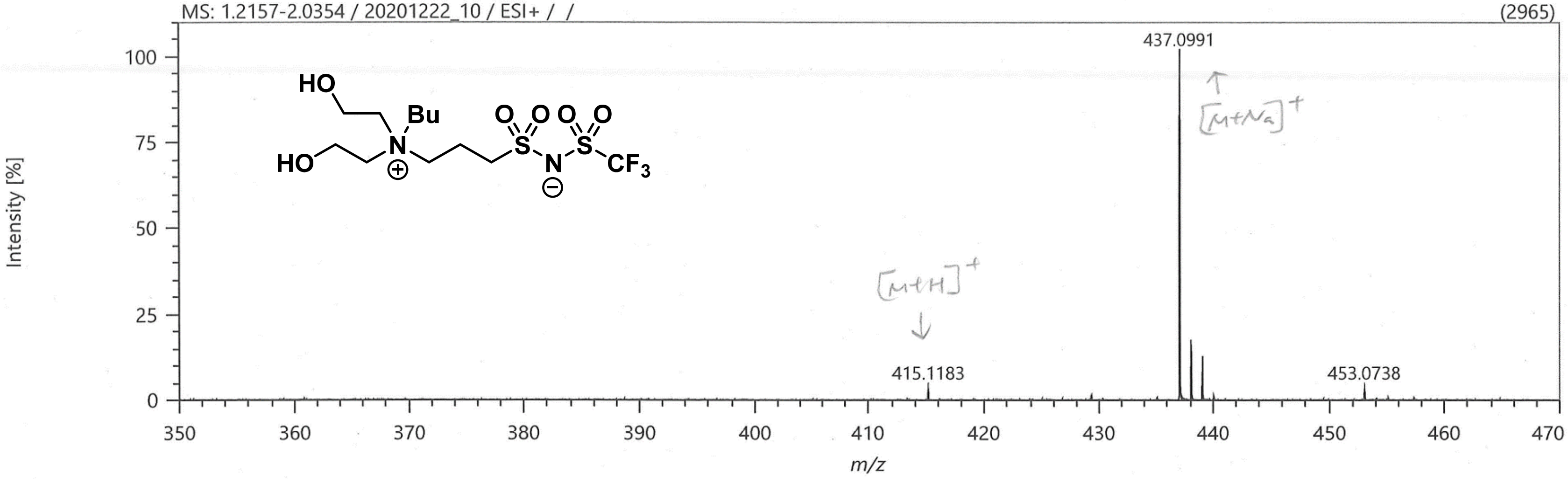

Elemental Composition

Parameters

Tolerance:	±2.00 ppm
Electron:	Odd/Even
Charge:	+1
DBE:	-99.0 - 999.0

Elements Set 1:

Symbol	C	H	F	N	O	S	Na
Min	0	0	3	2	6	2	0
Max	400	1000	3	2	6	2	1

Results

Mass	Formula	Calculated Mass	Mass Difference [mDa]	Mass Difference [ppm]	DBE
415.11831	C12 H26 N2 O6 F3 S2	415.11789	0.42	1.02	-0.5
437.09914	C12 H25 N2 O6 F3 Na S2	437.09983	-0.70	-1.59	-0.5

^{1}H NMR spectrum of **ZIL 4d-ene**

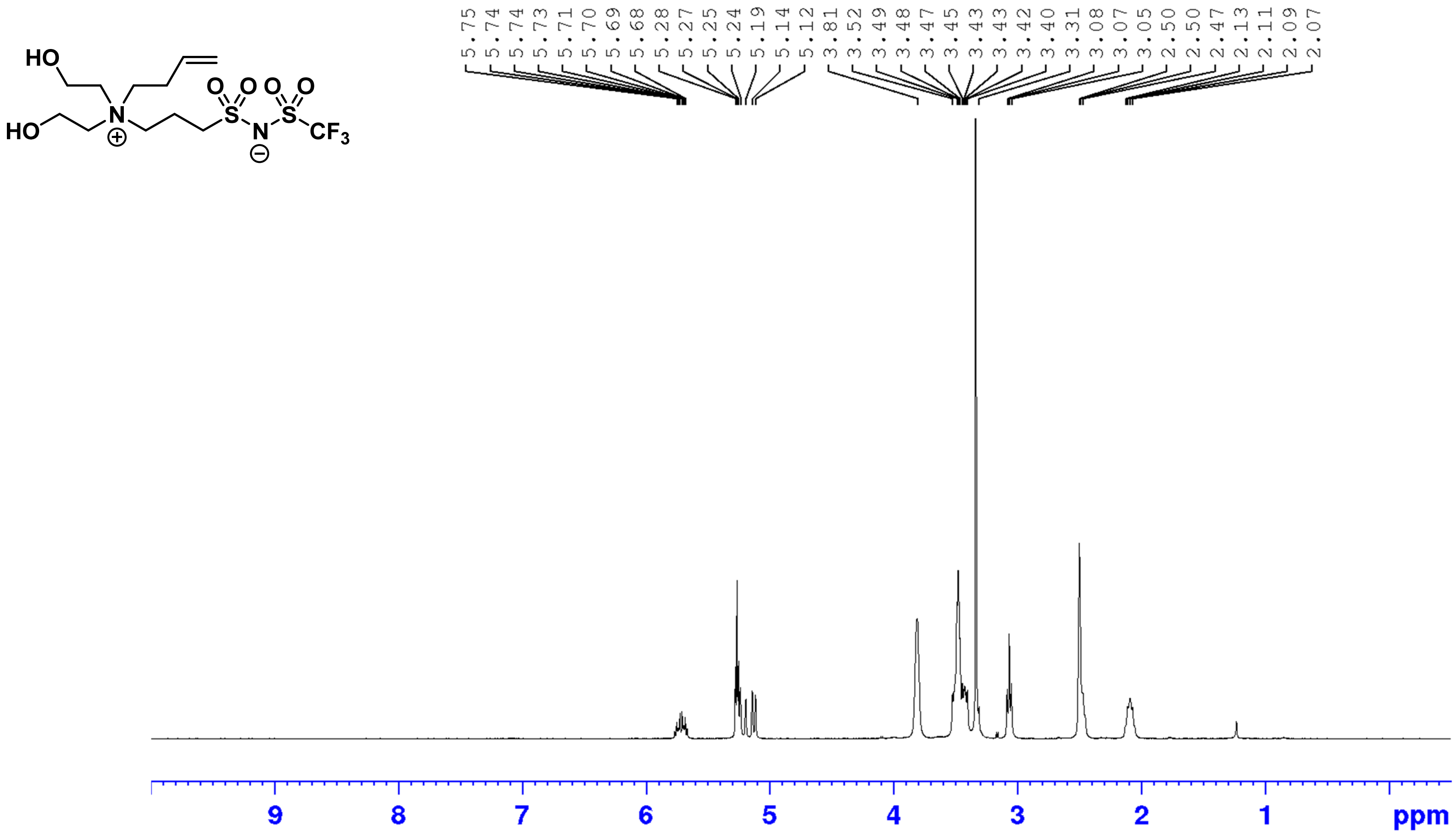

^{19}F NMR spectrum of **ZIL 4d-ene**

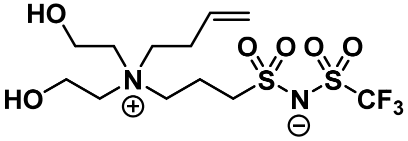

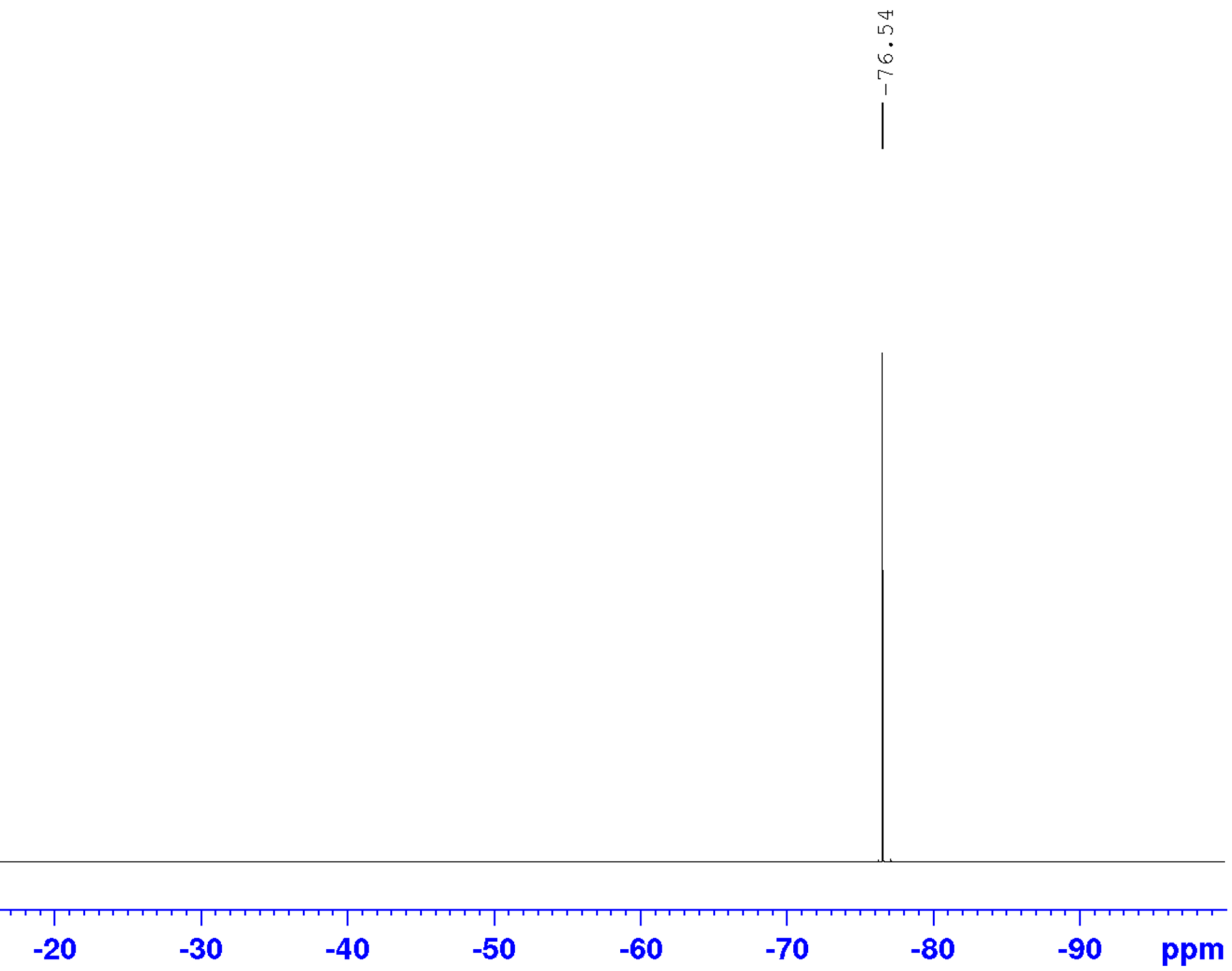

^{13}C NMR spectrum of **ZIL 4d-ene**

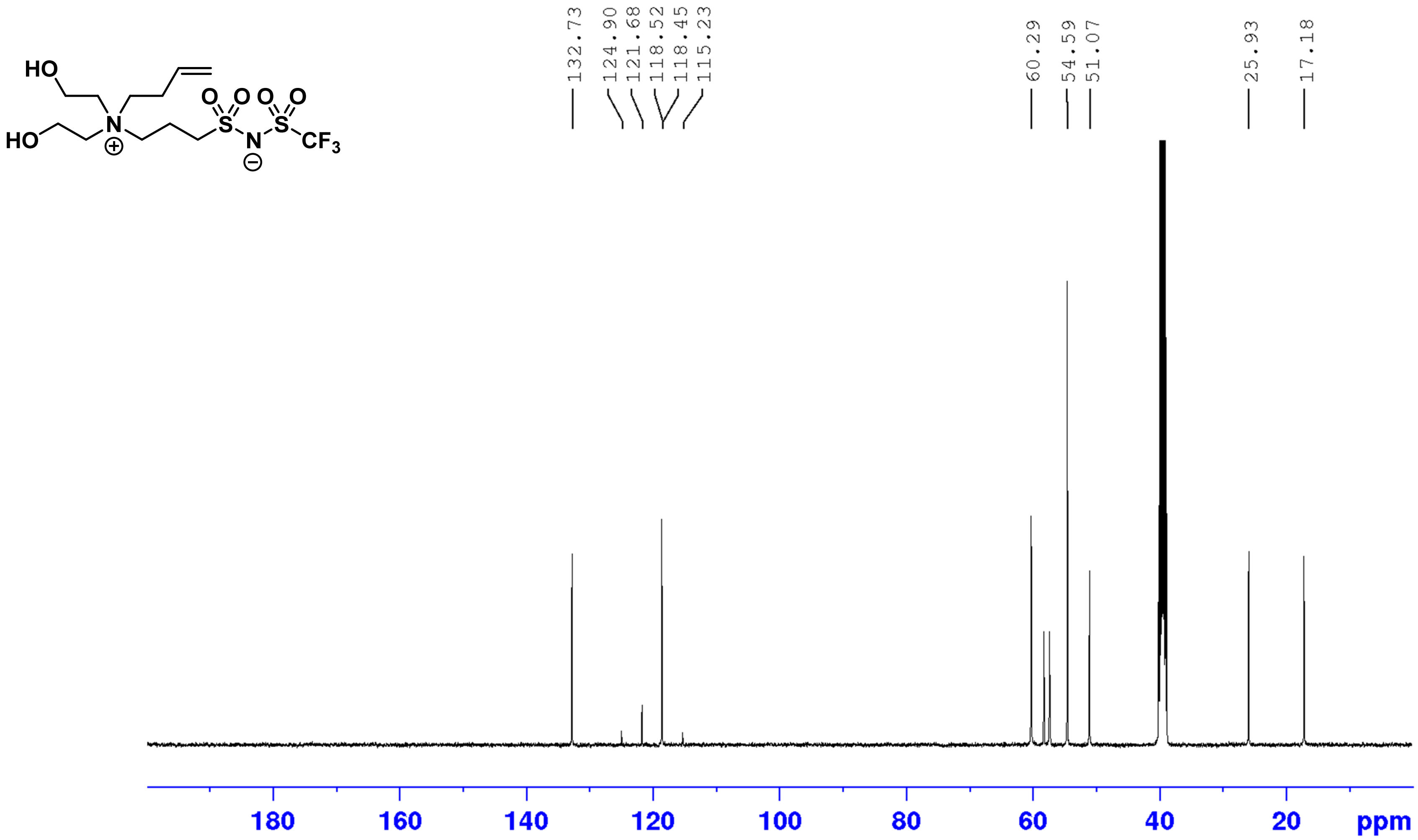

Mass spectrum of **ZIL 4d-ene**

Spectrum

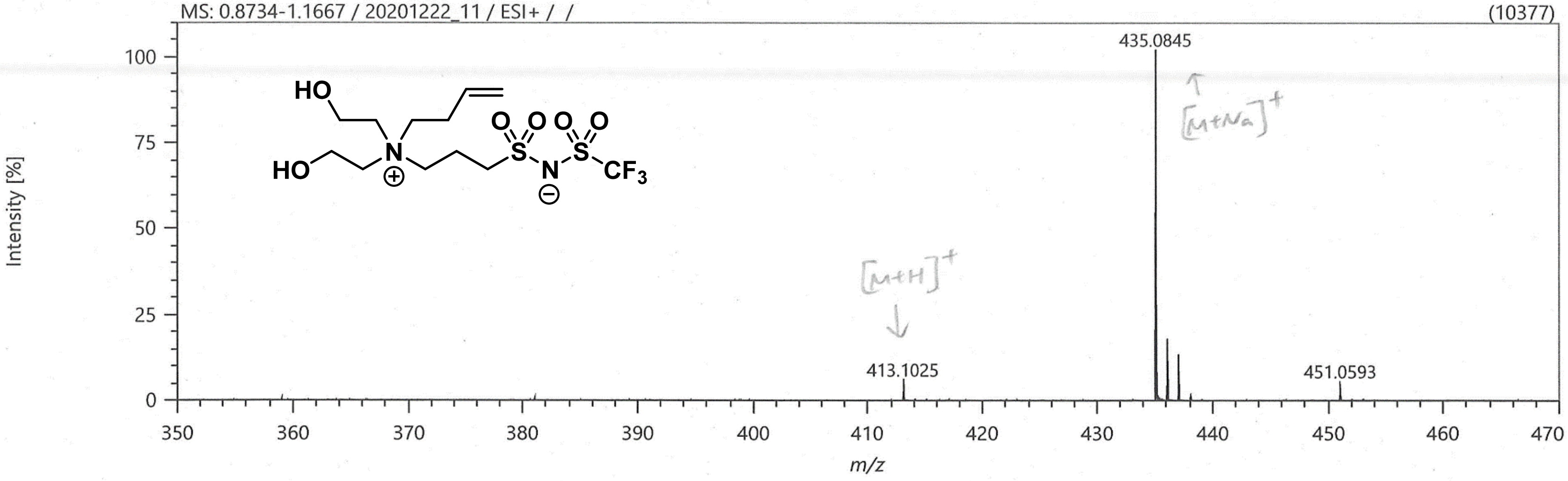

Elemental Composition

Parameters		Elements Set 1:							
Tolerance:	±2.00 ppm	Symbol	C	H	F	N	O	S	Na
Electron:	Odd/Even	Min	0	0	3	2	6	2	0
Charge:	+1	Max	400	1000	3	2	6	2	1
DBE:	-99.0 - 999.0								

Results

Mass	Formula	Calculated Mass	Mass Difference [mDa]	Mass Difference [ppm]	DBE
413.10250	C12 H24 N2 O6 F3 S2	413.10224	0.26	0.62	0.5
435.08454	C12 H23 N2 O6 F3 Na S2	435.08418	0.35	0.81	0.5

^{1}H NMR spectrum of **ZIL 4d-yne**

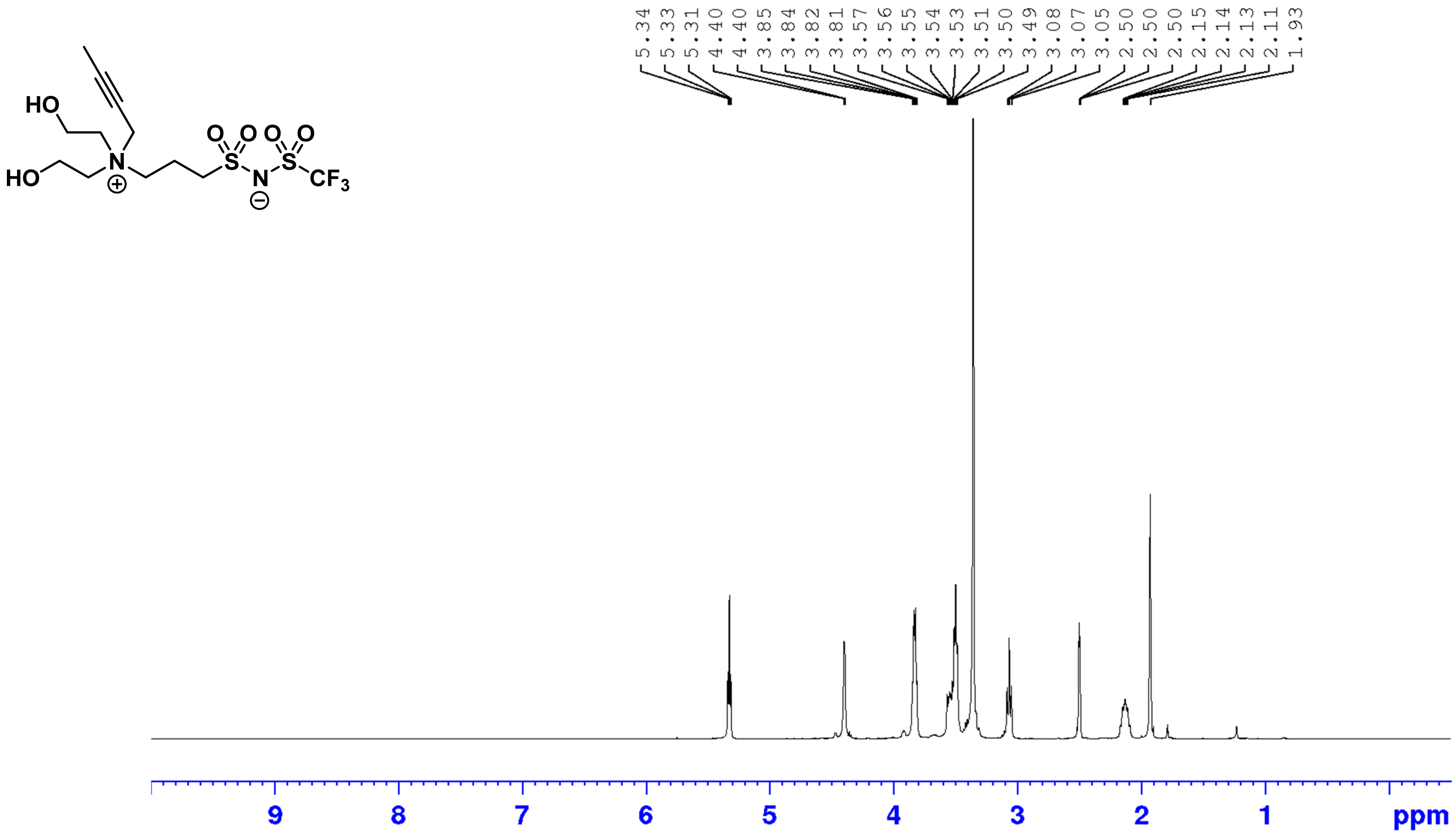

^{19}F NMR spectrum of **ZIL 4d-yne**

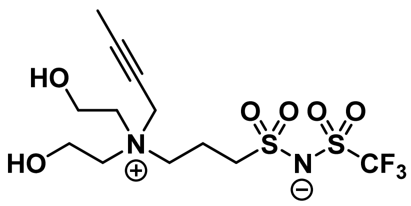

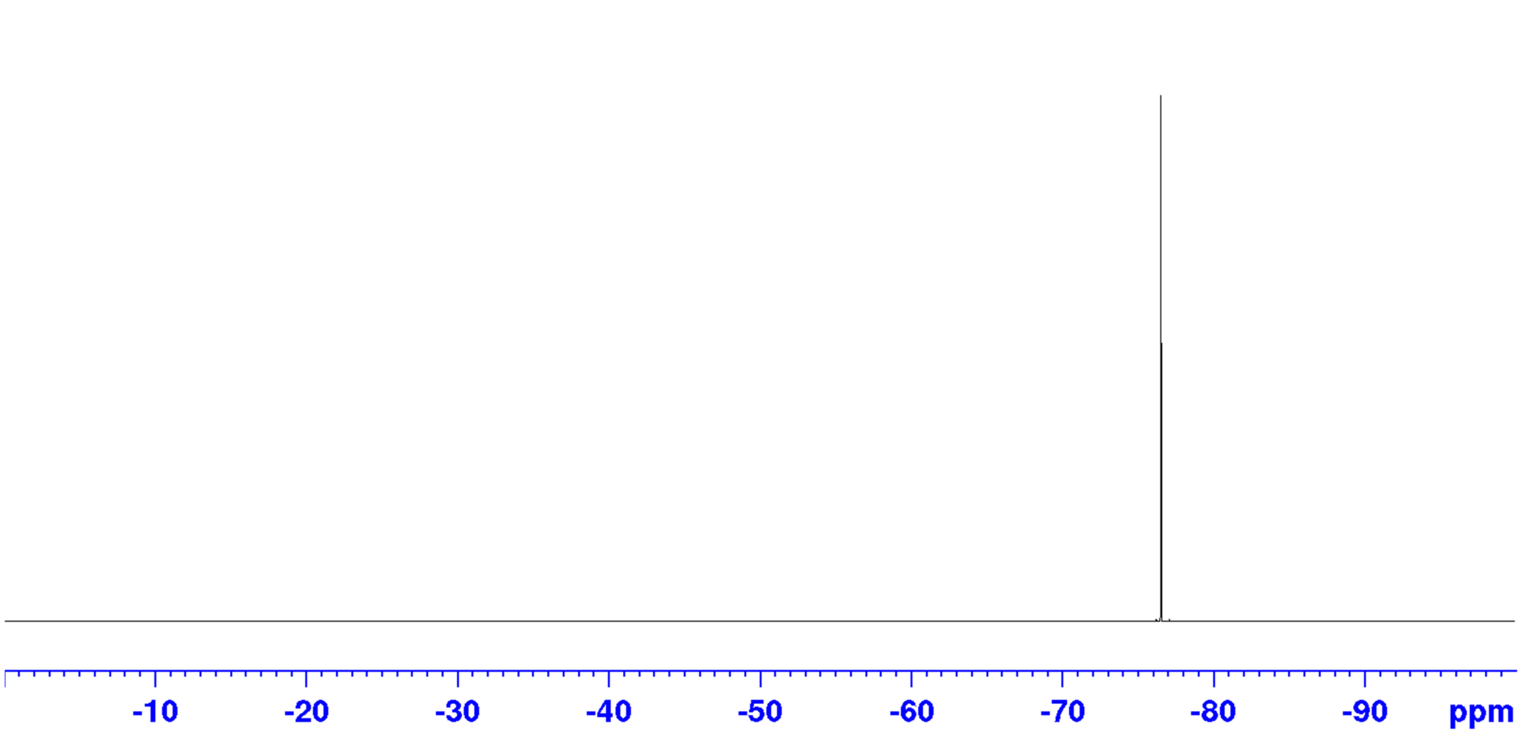

^{13}C NMR spectrum of **ZIL 4d-yne**

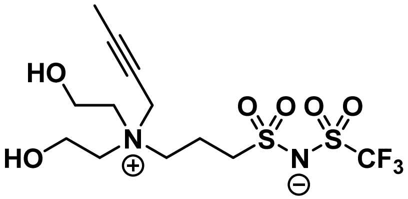

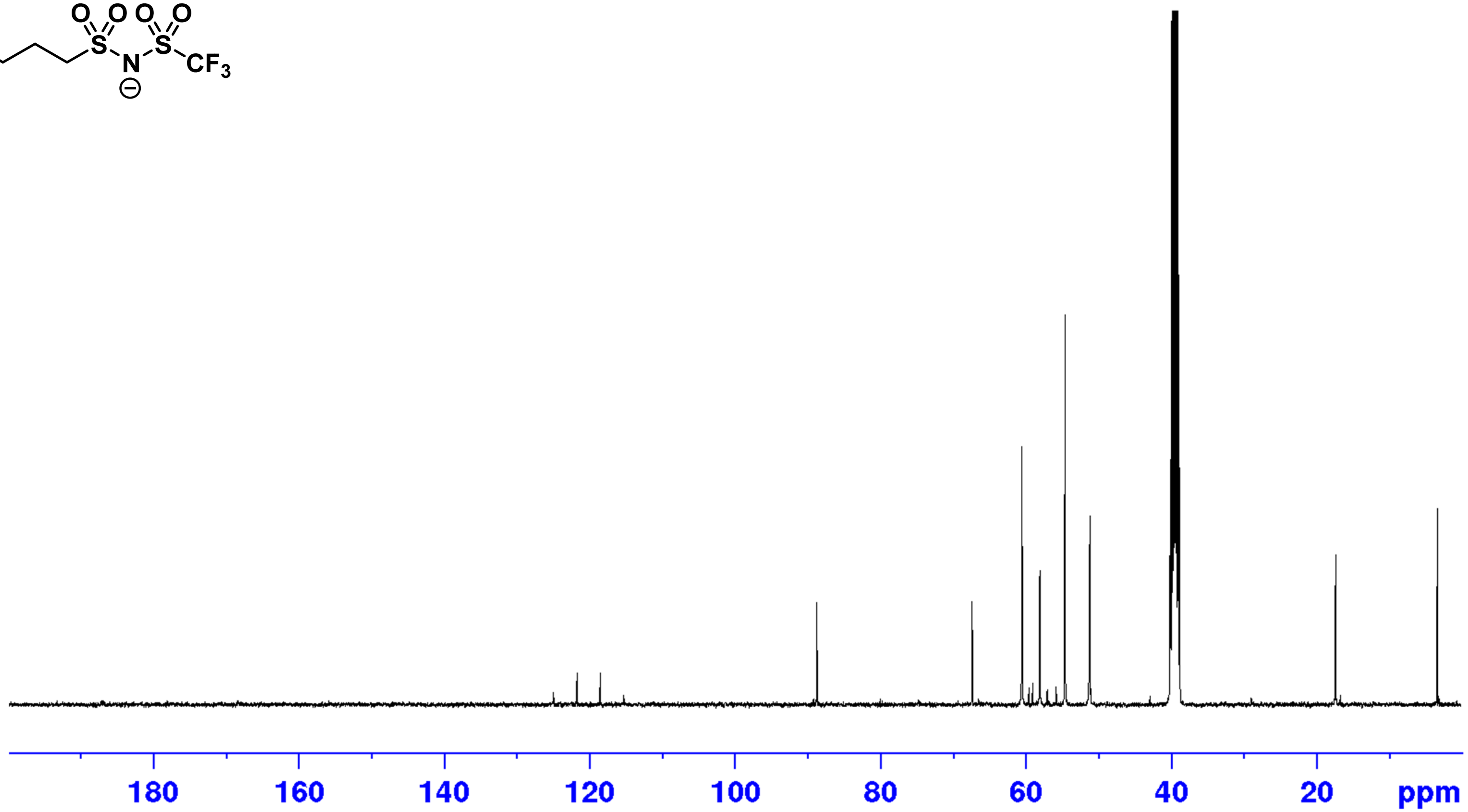

Mass spectrum of **ZIL 4d-yne**

Spectrum

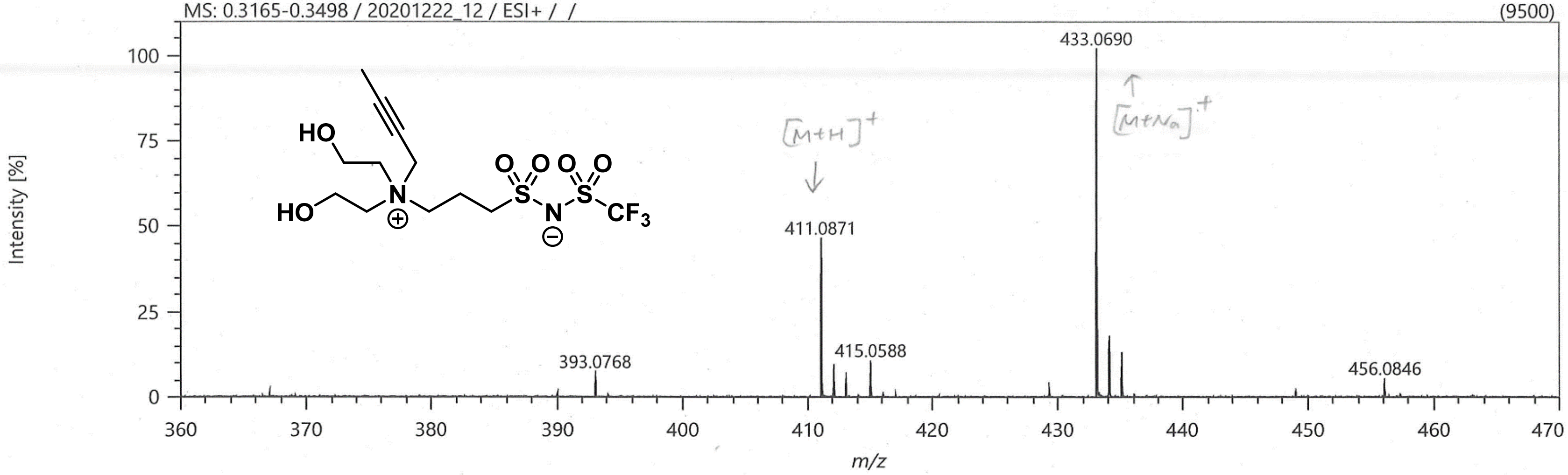

Elemental Composition

Parameters	
Tolerance:	±2.00 ppm
Electron:	Odd/Even
Charge:	+1
DBE:	-99.0 - 999.0

Elements Set 1:

Symbol	C	H	F	N	O	S	Na
Min	0	0	3	2	6	2	0
Max	400	1000	3	2	6	2	1

Results

Mass	Formula	Calculated Mass	Mass Difference [mDa]	Mass Difference [ppm]	DBE
411.08708	C12 H22 N2 O6 F3 S2	411.08659	0.49	1.19	1.5
433.06901	C12 H21 N2 O6 F3 Na S2	433.06853	0.48	1.11	1.5

^{1}H NMR spectrum of **ZIL 4e**

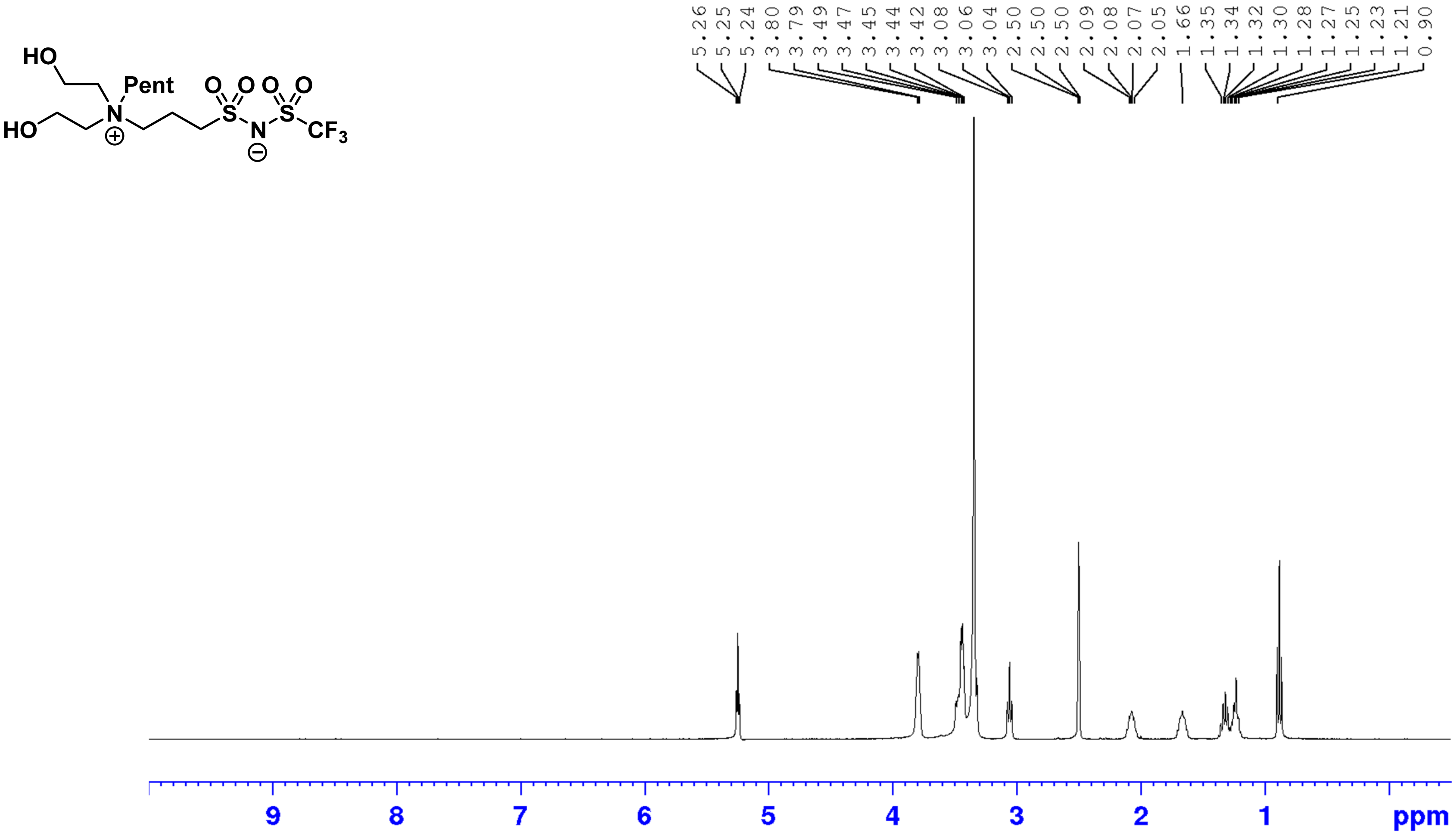

^{19}F NMR spectrum of **ZIL 4e**

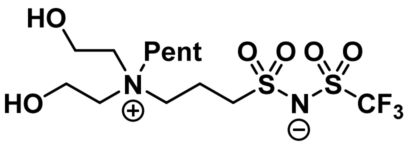

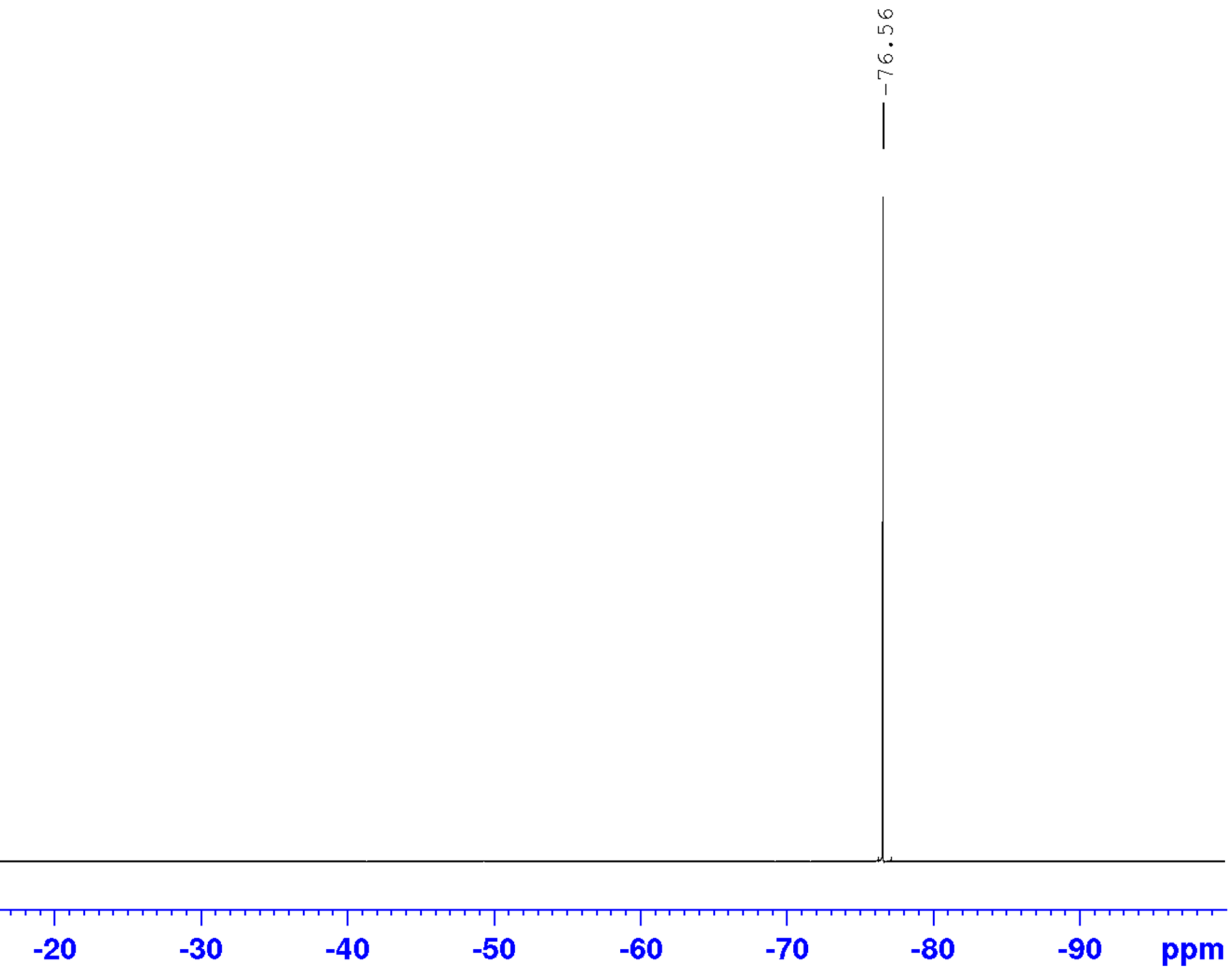

^{13}C NMR spectrum of **ZIL 4e**

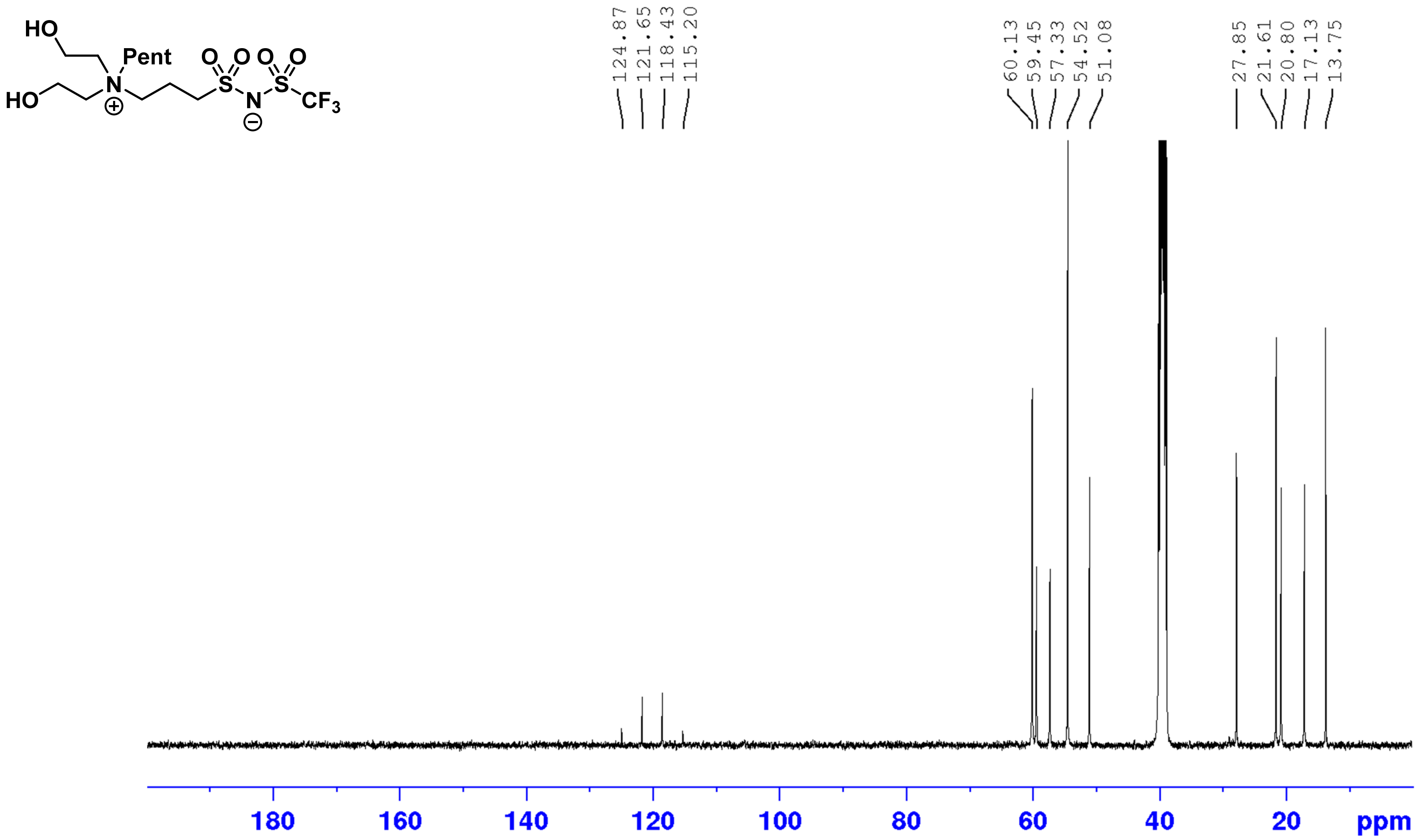

Mass spectrum of **ZIL 4e**

Spectrum

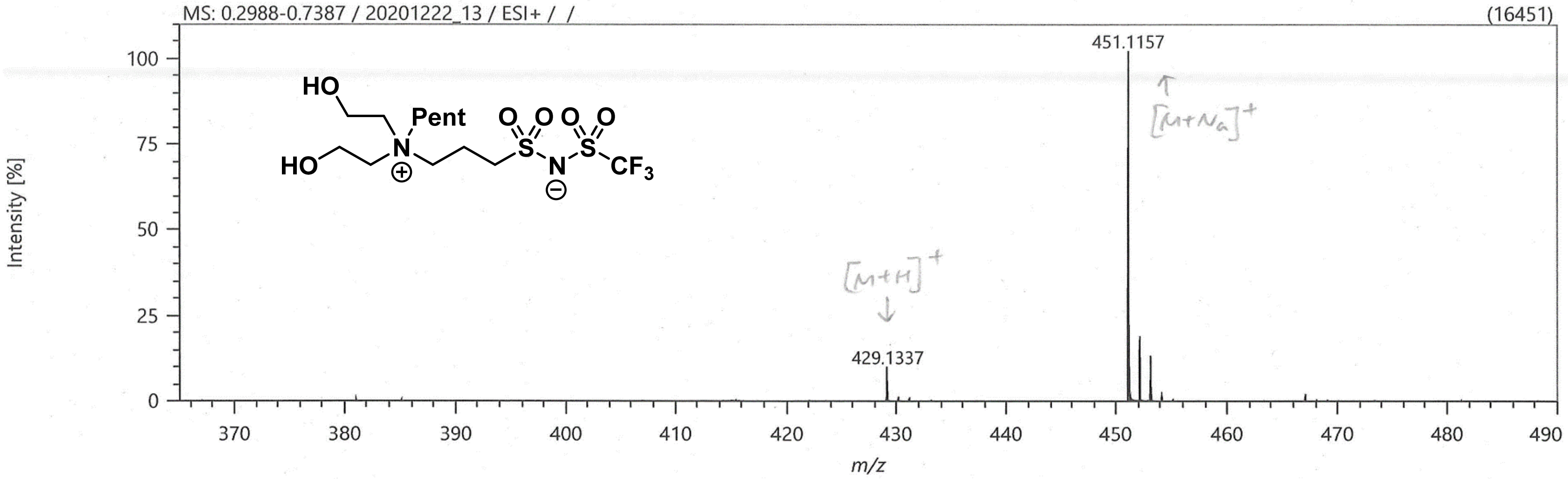

Elemental Composition

Parameters		Elements Set 1:							
Tolerance:	±2.00 ppm	Symbol	C	H	F	N	O	S	Na
Electron:	Odd/Even	Min	0	0	3	2	6	2	0
Charge:	+1	Max	400	1000	3	2	6	2	1
DBE:	-99.0 - 999.0								

Results

Mass	Formula	Calculated Mass	Mass Difference [mDa]	Mass Difference [ppm]	DBE
429.13368	C13 H28 N2 O6 F3 S2	429.13354	0.14	0.34	-0.5
451.11574	C13 H27 N2 O6 F3 Na S2	451.11548	0.26	0.57	-0.5

^{1}H NMR spectrum of **ZIL 4f**

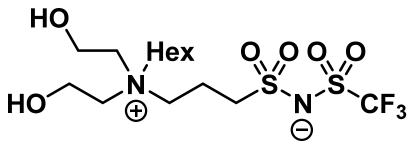

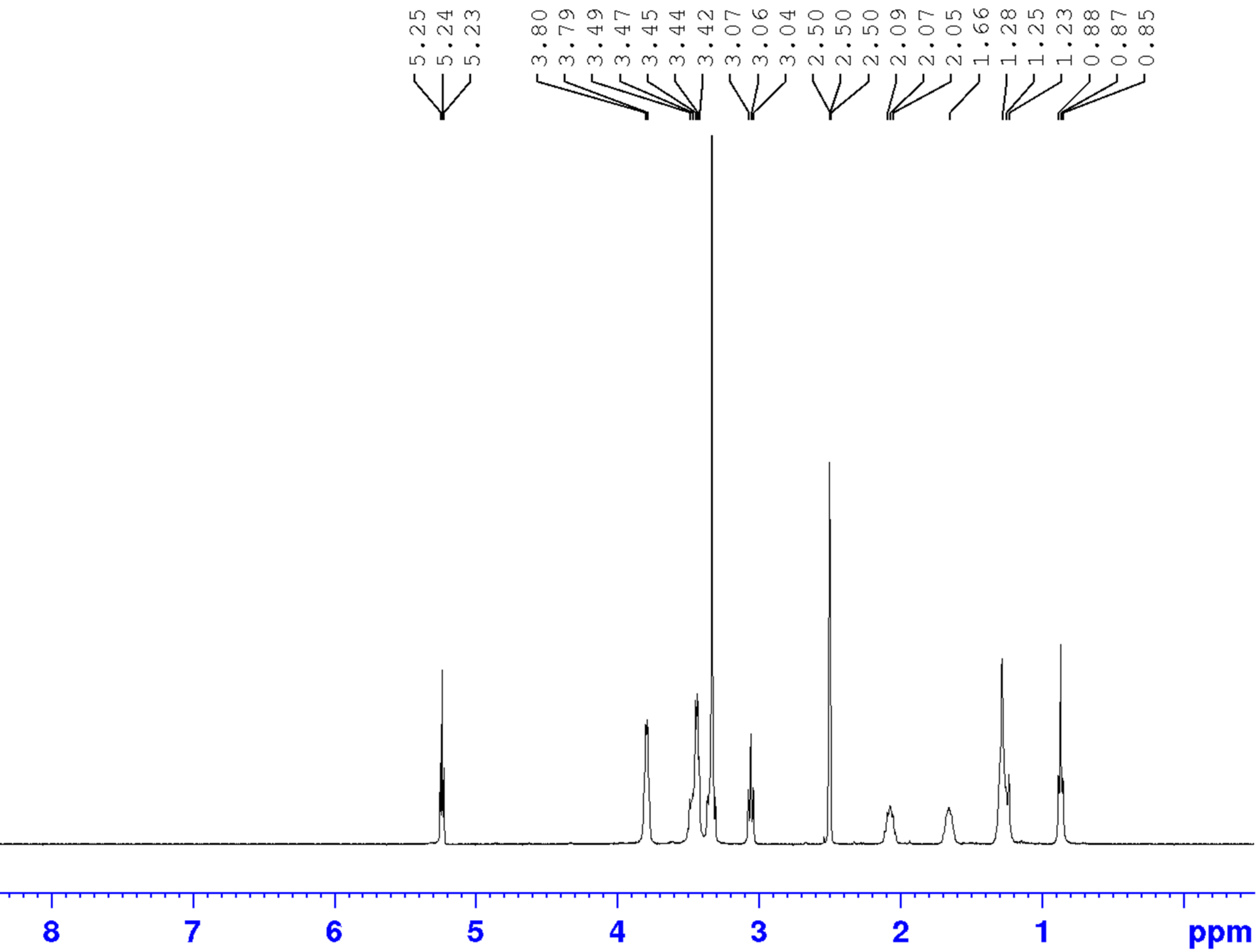

^{19}F NMR spectrum of **ZIL 4f**

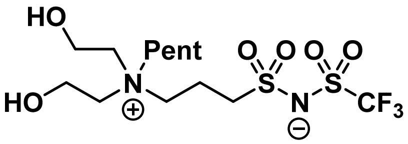

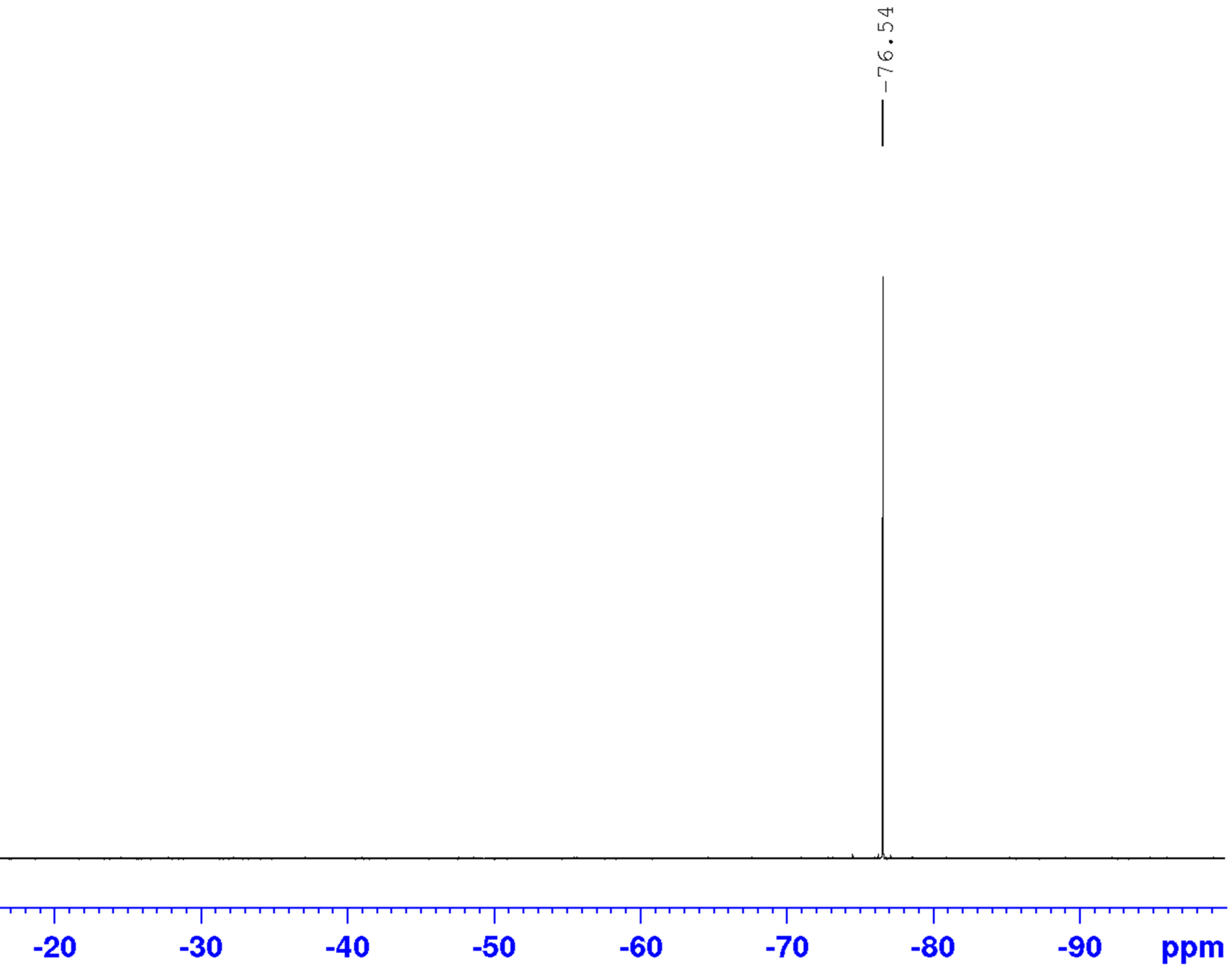

^{13}C NMR spectrum of **ZIL 4f**

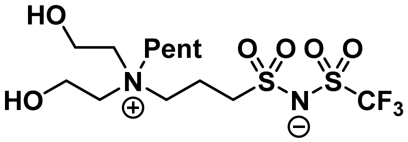

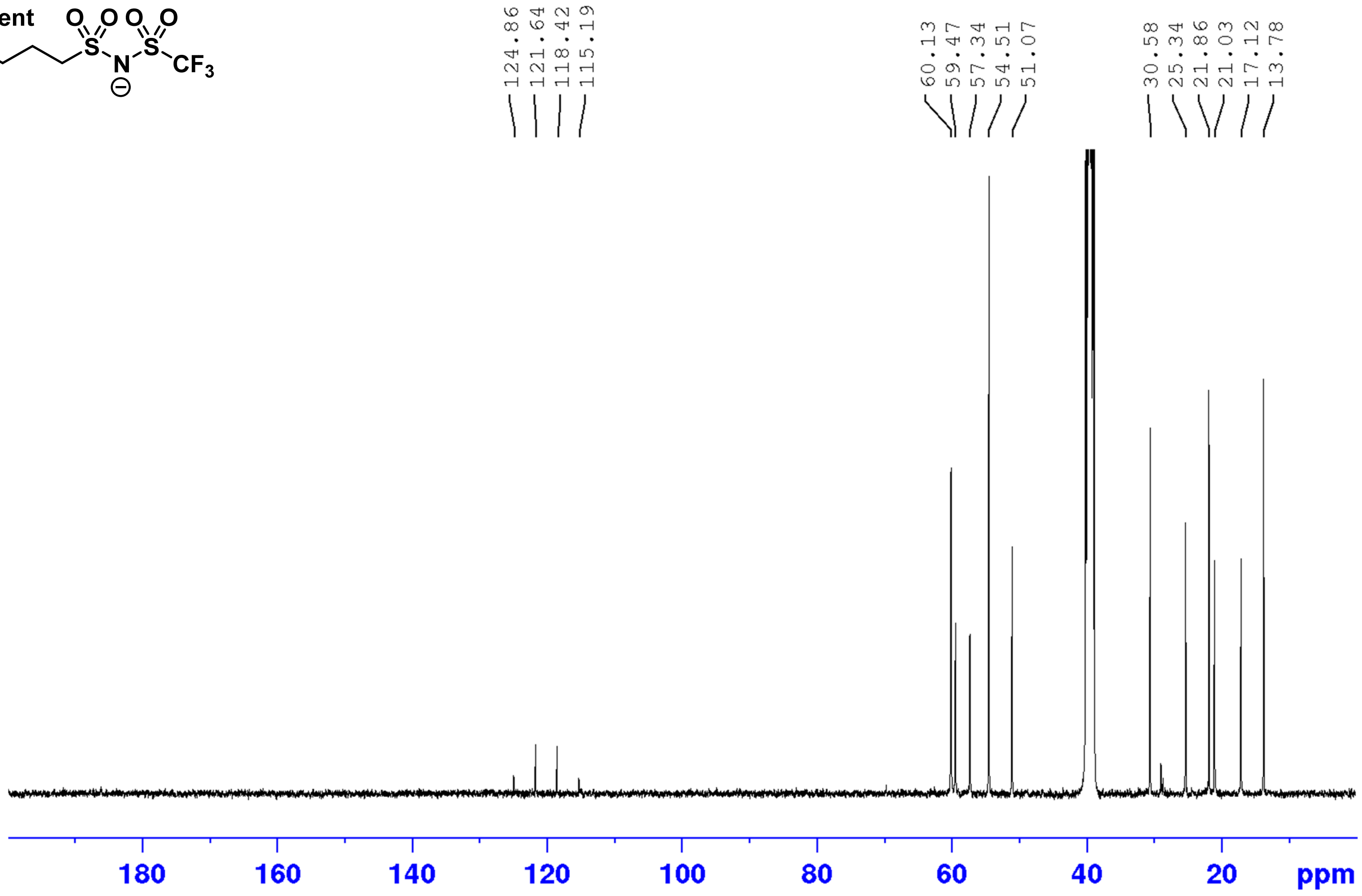

Mass spectrum of **ZIL 4f**

Spectrum

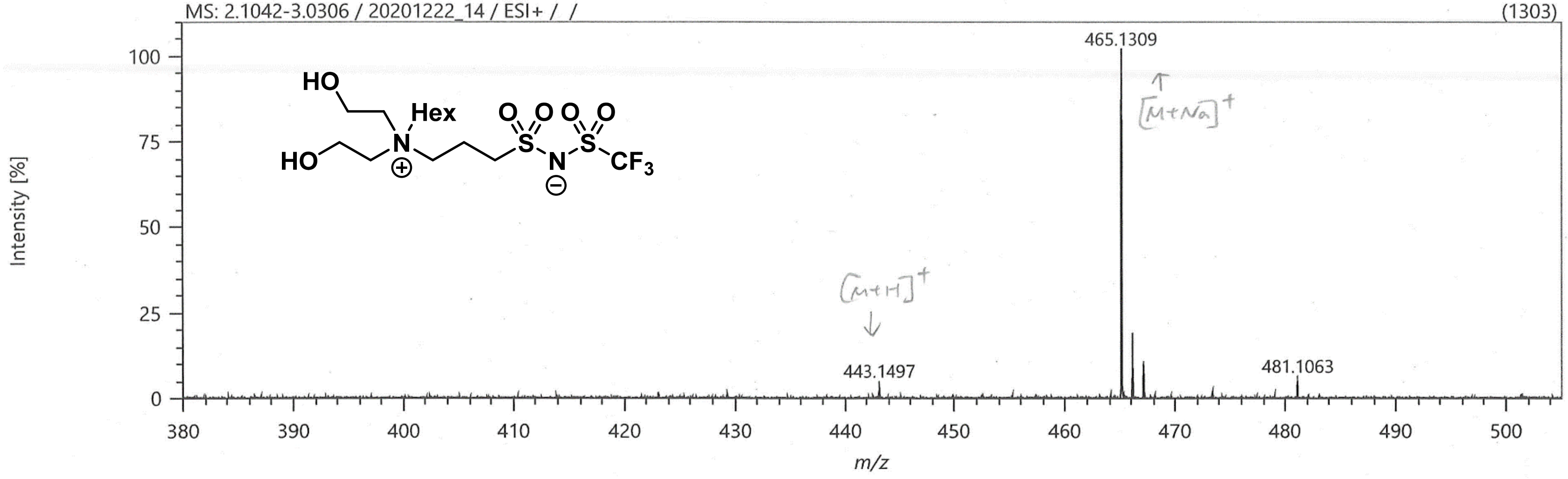

Elemental Composition

Parameters		Elements Set 1:							
Tolerance:	±2.00 ppm	Symbol	C	H	F	N	O	S	Na
Electron:	Odd/Even	Min	0	0	3	2	6	2	0
Charge:	+1	Max	400	1000	3	2	6	2	1
DBE:	-99.0 - 999.0								

Results

Mass	Formula	Calculated Mass	Mass Difference [mDa]	Mass Difference [ppm]	DBE
443.14969	C14 H30 N2 O6 F3 S2	443.14919	0.50	1.13	-0.5
465.13089	C14 H29 N2 O6 F3 Na S2	465.13113	-0.24	-0.53	-0.5
